# Efficacy of biomarkers in the endochondral phase of fracture repair and healing in long bones: A clinical observational studys

**DOI:** 10.1371/journal.pmed.1004640

**Published:** 2025-08-29

**Authors:** Zachary M. Working, Molly E. Czachor, Anna Laura Nelson, Kelsey M. O’Hara, Karalynn Lancaster, Justin E. Hellwinkel, Kaitlyn Whitney, Lucas S. Marchand, Sofia Bzovskys, Graham J. DeKeyser, Darin M. Friess, Theodore Miclau, William A. Horton, Sheila A. Sprague, Nathan N. O’Hara, Brian Johnstone, Lauren A. Pierpoint, Gerard P. Slobogean, Chelsea S. Bahney, On behalf of the Vita-Shock Investigators

**Affiliations:** 1 Oregon Health & Science University, Portland, Oregon United States of America; 2 Steadman Philippon Research Institute, Vail, Colorado United States of America; 3 University of Utah, Salt Lake City, Utah, United States of America; 4 McMaster University, Hamilton, Ontario, Canada; 5 University of California, San Francisco (UCSF) Orthopaedic Trauma Institute, San Francisco, California United States of America; 6 Shriners Hospital for ChildrenPortland, Oregon United States of America; 7 University of Maryland School of Medicine, Baltimore, Maryland United States of America; Université Paris Cité UFR de Médecine: Universite Paris Cite UFR de Medecine, FRANCE

## Abstract

**Background:**

Clinical determination of bone fracture healing remains qualitative, typically determined through the combination of plain film radiographs, clinical assessment, and patient-reported pain. Importantly, standard radiographs can only detect bone formation in the fracture site after sufficient tissue mineralization has occurred, restricting utility to the later stages of bone repair. A more rigorous method for determining fracture healing progression could significantly improve patient care. Quantitative biomarkers have gained diagnostic value in many clinical indications. Multiple bone turnover biomarkers have been successfully utilized for diagnosis and clinical management of osteoporosis. However, there remains limited evidence for the correlation and diagnostic efficacy of these biomarkers when applied to fracture repair.

**Methods and findings:**

Here we present data from a large cohort of participants without (*n* = 111) or with (*n* = 153) fracture with the primary outcome of interest our blood-based biomarker which detects the degradation product of the trimeric collagen X protein (CXM). Collagen X is a transient extracellular matrix protein synthesized by hypertrophic chondrocytes during the soft callus phase of endochondral fracture repair. Our data show that healthy patients with an age range of 21−85 years without fracture (enrolled 2018−2020) have low circulating levels of CXM (median = 563.4 pg/mL; interquartile range (IQR) [483.2, 771.1]) that do not vary independently with age (*r* = 0.04, *p* = 0.26) or sex (*p* = 0.42). Fracture data includes patients with an acute closed or low grade open (Gustilo type I or II) tibial or femoral shaft fracture from a secondary analysis of the VitaShock study (NCT02786498, *n* = 102: enrolled through the University of Maryland 2016−2019), complemented by a new prospectively enrolled observational study that recruited from the Oregon Health & Science University level 1 academic trauma center (*n* = 51: enrolled 2019−2023) that were followed until the patient was deemed clinically healed or until they failed to return for follow-up. We show that in serum CXM positively correlates to the bone biomarkers N-terminal propeptide of type I procollagen (P1NP, *r* = 0.50, *p* < 0.0001), C-terminal telopeptide of type I collagen (CTX, *r* = 0.40, *p* < 0.0001), and osteocalcin (*r* = 0.26, *p* = 0.0411); with a negative correlation to the obesity biomarker leptin (*r* = −0.31, *p* = 0.0138). Further, patients with early healing exhibited a peak in CXM at 6 weeks of 1,092 pg/mL (95% confidence interval (CI) [804.8, 1,379]), which was significantly higher than patients with normal healing of 630.8 pg/mL (95% CI [399.9, 861.8]; *p* = 0.016). We then show that we can reliably transfer this assay from serum collection through venipuncture to a dried blood spot (DBS) collected by finger prick (*r* = 0.75, Serum = 0.4217*DBS + 181.9, *p* < 0.0001). Using DBS, the prospective clinical observational study finally suggests that median time-to-peak CXM is at 25.5 days following the elimination of outliers (*n* = 6) using Robust Regression and Outlier Removal (ROUT, Q = 1%). This study did not find significant differences in CXM expression according to age, sex, or bone that are likely due to the main limitation of an observational study.

**Conclusion:**

This rigorous data set supports the future prospective use of the CXM biomarker collected by finger prick in interventional fracture studies and/or in observational studies requiring quantitative assessments of long-bone healing.

## Author summary

**Why was this study done?:**

- Standard of care to evaluate fracture healing relies on qualitative assessment of bone formation on radiographs (X-ray).
- There are no established quantitative methods to diagnose the progression of fracture healing.
- This study is a large-scale clinical assessment of a blood-based biomarker to collagen X (CXM), which specifically measures the conversion of cartilage to bone during long bone healing.

**What did the researchers do and find?:**

- CXM biomarker is expressed at very low levels in participants without a fracture and does not vary with age or sex.
- CXM can be reliably measured in both serum or dried blood spots in patients following fracture and expression patterns were validated against existing bone turnover markers.
- CXM expression peaks around 25 days post-fracture and therefore could provide an earlier, or complementary, indication of healing relative to X-ray.

**What do these findings mean?:**

- This study supports that CXM could be a valuable biomarker to prospectively investigate in future clinical trials and the data from this study serve as the foundation to power hypothesis-driven designs.
- CXM may ultimately help answer open questions related to the temporal rate at which different bones heal, how patient demographics may influence the early phase of healing, predict non-union, or provide patient-specific healing algorithms.
- This study was limited in sample size and potential uncontrolled confounding factors associated with enrollment in a trauma setting.

## Abstract 3

Zachary M. Working and colleagues investigate the clinical relevance of collagen X breakdown product (CXM) as a blood-based biomarker to long bone fracture repair.

## Introduction

Bone fractures are a common injury worldwide with significant economic and social impact. A comprehensive, collaborative systematic analysis of global fracture incidence reported 178 million new fractures, 445 million prevalent fractures, and 25.88 million years lived with disability in 2019 [1,2]. Lower extremity long bone fractures, including femur and tibia/fibula fractures, accounted for 14.6 and 32.7 million of the total annual injuries, respectively [2].

Normal fracture repair is a dynamic, regenerative process that proceeds through four distinct, but overlapping stages of healing: *first*, a hematoma forms to contain debris and initiate a pro-inflammatory response that triggers repair [3–6], *second*, intramembranous bone forms along the periosteal surfaces of the bone adjacent to the fracture and a cartilage callus forms within the bone break to bridge the fracture gap [7], *third*, the cartilage callus is transformed to bone through the process of endochondral ossification (as recently reviewed [8,9]), and *fourth*, the bone remodels to regenerate the native cortical bone [10]. While significant progress has been made recently in defining the molecular and cellular events that coordinate fracture repair, the clinical diagnosis of healing remains rudimentary.

Clinical diagnosis of facture healing relies predominantly on radiographic evidence of bone repair. The Radiographic Union Score for Tibia (RUST) and modified RUST (mRUST) remain the only common clinical tools for scoring bone healing. These systems generate a semi-quantitative determination of bone bridging across the fracture using plain film radiographs [11–13].

A substantial subset of patients will experience delayed healing or nonunion, which are qualitative clinical diagnoses based on the persistence of a fracture line in longitudinal radiographs and pain/ instability with weight bearing. There remains no census on clinical criteria defining delayed or nonunion in fracture care. Nonunion are generally fractures that will not heal without further intervention, with a published window of diagnosis ranging from 3–12 months [14,15]. There is even less guidance on the clinical classification for delayed union. While robust estimates for the global incidence of delayed and nonunion are not available, current studies report that 8.1% of fractures were readmitted for healing complications within 2 years of injury, with higher rates found in femoral (13.6%) and tibial (11.7%) shaft fractures [16], and that 7%–10% of surgically treated long bone fractures in the US go on to nonunion [17].

Compromised fracture healing has a large societal burden, significantly increasing treatment costs and decreasing patient quality of life [18–20]. Tibial nonunion [21], femoral nonunion [22], and humeral nonunion [23] proved to be diagnoses more impactful than congestive heart failure and myocardial infarction in health-related quality-of-life measures. Hypotheses exist regarding which medical factors are associated with nonunion formation, but there remain no conclusive studies delineating the contribution of factors such as age, sex, concomitant medical diagnoses, and patient behavior [24].

Reliable biomarkers are an attractive option to address the limitation of qualitative radiographic measurements of fracture healing yet no clinical biomarker to date has demonstrated adequate efficacy or sensitivity [25]. The largest class of bone biomarkers is a category of bone turnover markers developed for the diagnosis and clinical management of osteoporosis [26]. There remains limited consensus on their utility in fracture healing since most studies include under 30 patients [27,28]. Recently, the VitaShock trial consisting of 102 patients presenting with a fracture confirmed that the concentration of N-terminal propeptide of type I procollagen (P1NP, a bone formation marker) and C-terminal telopeptide of type I collagen (CTX, a bone resorption marker bone marker) increase during acute fracture healing with elevated levels at 6 weeks post-injury associated with early radiological healing at 12 weeks [29].

Unlike the classic bone turnover markers studied by others, we have reported on a degradation product of collagen X (CXM) as an investigational biomarker associated with the endochondral remodeling of cartilage to bone [30]. Collagen X is the hallmark extracellular matrix protein synthesized by the hypertrophic chondrocyte during the conversion of cartilage to bone during long bone fracture repair [31,32]. In preclinical models, CXM expression peaks during the soft callus phase of healing and is subsequently resolved during bone remodeling [33]. This biomarker to the cartilaginous phase of fracture repair is biologically distinct from bone remodeling, detecting a stage of fracture healing not distinguishable on standard radiographs.

The goal of this exploratory study was to characterize CXM response to lower extremity long bone fracture in a large group of patients and establish the foundational data to support definitive studies for clinical adoption. We aimed to contextualize the fracture response against healthy individuals without fracture to understand baseline variations of the biomarker with age and sex. With fracture, we then determined to degree of correlation between CXM and other biomarkers of bone turnover, age, and obesity. By segregating data according to patient demographics (age, sex, mRUST score, fracture location), we hypothesize that CXM will correlate with established bone biomarkers but peak in expression earlier during healing.

## Materials and methods

### Ethics statement

Formal written informed consent was obtained from adult (≥18 years of age) study participants according to the following study protocols:

1. Uninjured Participants: Institutional Review Board (IRB) approval obtained from Vail Health under protocol #2018-48.
2. Secondary Analysis of VitaShock Trial: Approval for the study was obtained by the Hamilton Integrated Research Ethics Board (2017-1952) and the University of Maryland IRB (HP-00069705).
3. Prospective fracture observational study: This study enrolled patients under independent IRB protocols from the Oregon Health & Science University (OHSU, #00019234) and Vail Health (#2018-48).

### Study design

The objective of this study was to evaluate the expression patterns of the CXM biomarker following tibia and femur fracture and compare expression patterns to existing metrics of fracture healing and to study participants without a fracture. Fractures included in this analysis were limited to diaphyseal tibia and femur as lower limb fractures have a higher incidence of nonunion [16], are both weight-bearing bones, and heal through the same molecular pathways (endochondral ossification). This multicenter collaborative study contained three distinct cohorts of patients: (i) healthy, uninjured volunteers, (ii) a secondary analysis of the VitaShock clinical trial (identifier NCT02786498), and (iii) prospectively enrolled fracture patients at two academic centers. Together, these studies allowed us to significantly expand the total patient population for increased study rigor and adjust the timeline of sample collection from 6 weeks in the retrospective to 3 weeks in the prospective study in response to preliminary data analysis.

### Participant eligibility criteria and sample collection

#### Uninjured participants.

Healthy, uninjured volunteers were recruited from the community through the Steadman Philippon Research Institute (SPRI) according to the IRB Approval obtained from Vail Health under protocol #2018-48. These participants serve as a control/baseline. Written informed consent was obtained from all participants. Following enrollment, participants completed a medical questionnaire and gave a single blood sample by venipuncture and/or finger prick. Patients were over the age of 18, male and female, smokers and non-smokers, could be taking medications, and could have underlying endocrine or metabolic bone disorders. Participants who met any of the following criteria were excluded from the study:

1. Pregnant individuals
2. Participants with genetic diseases affecting chondrogenesis, including chondroplasia (Defects in collagen X have been related to Schmid-type metaphyseal chondrodysplasia)
3. Participants with malignancies known to produce high levels of serum collagen 10A1 (specifically adenoma, colon cancer, and several solid tumors)
4. Participants with a previous fracture or osteotomy procedure within the last 12 months
5. History of blood-borne transmissible disease, including human immunodeficiency virus (HIV), acquired immunodeficiency syndrome (AIDs), and Hepatitis C
6. Participants that have any condition, including laboratory findings and findings in the medical history or the pre-study assessments, that, in the opinion of the Investigator constitutes a risk or contraindication for participation in the study or that could interfere with the study objectives, conduct, or evaluation or prevent the patient from fully participating in all aspects of the study.

#### Secondary analysis of VitaShock trial.

A secondary analysis of serum samples from 102 patients with a fracture was collected as part of the VitaShock phase II exploratory randomized clinical trial comparing the effect of multiple vitamin D_3_ dosing strategies on fracture healing in patients with isolated lower extremity long bone fractures (NCT02786498). Approval for the study was obtained by the Hamilton Integrated Research Ethics Board (2017-1952) and the University of Maryland IRB (HP-00069705). A Material Transfer Agreement (MTA) was established for sample transfer between the University of Maryland and SPRI. All metrics of the study, including patient exclusion criteria, inclusion criteria, and overall study design, have previously been published [34]. Briefly, eligible patients included tibia and femur fracture patients within 7 days of injury, aged 18–50 years, with a closed or low-grade open (Gustilo-Anderson class I or II) fracture treated by reamed intramedullary nailing at the R Adams Cowley Shock Trauma Center at the University of Maryland. Patients with osteoporosis, stress fractures, serum calcium >10.5 mg/dl, atypical femur fractures, pathological fractures secondary to bone lesions, underlying disorders of bone metabolism, hyper-homocysteinemia, or vitamin D allergies were excluded. Written informed consent was obtained from all eligible participants. Patients followed up at 6 weeks and 12 weeks. Primary study analysis and outcomes have also been reported [29]*.* A secondary analysis of protein biomarkers was performed on serum collected during the primary VitaShock trial, as detailed below.

#### Prospective observational study.

Patients with a fracture were recruited from the Oregon Health & Science University (OHSU) level 1 academic trauma center and The Steadman Clinic under independent, but aligned, IRB-approved protocols (OHSU #00019234: TSC #2018-48), STROBE reporting for this study (S1 STROBE Checklist). Male and female patients over the age of 18 with isolated open or closed femur or tibia fractures (AO/OTA type 42) treated either surgically or non-surgically within 14 days were screened for eligibility. Patients who met any of the following criteria were excluded from the study:

1. Pregnant patients
2. Pathological fractures due to tumors, radiation, or connective tissue disorders
3. Genetic diseases affecting chondrogenesis, including chondroplasia
4. A critical-size defect requiring bone grafting
5. Type II or III open fractures in the Gustilo-Classification
6. Malignancies known to produce high levels of serum collagen 10A1
7. Previous fracture or osteotomy procedure within the last 12 months
8. History of blood-borne transmissible disease, including HIV, AIDs, and Hepatitis C
9. Any condition, including laboratory findings and findings in the medical history or in the pre-study assessments, that in the opinion of the Investigator constitutes a risk or contraindication for participation in the study or that could interfere with the study objectives, conduct or evaluation, or prevent the patient from fully participating in all aspects of the study
10. Patients sustaining multiple fractures.

Eligible patients were offered enrollment and written informed consent was obtained. In this prospective observational study, enrollment in the study was independent of patient care and did not impact patient treatment. Longitudinal blood samples were collected at all standard-of-care clinical visits (average ± standard deviation (SD): 3 weeks ± 1.9 weeks, 8 weeks ± 5.4 weeks, 15 weeks ± 7.3 weeks, 24 weeks ± 10.1 weeks, 40 weeks ± 19.0 weeks, and 48 weeks ± 18.6 weeks) using a microtainer contact-activated lancet to prick the finger and obtain five drops of blood collected onto a Whatman 903 Protein saver card. Patients were followed until they were deemed clinically healed or until they failed to return for follow-up care.

The descriptive observational study was designed to collect longitudinal fracture healing data for the CXM biomarker against standard of care radiographic outcome measures (mRUST) with the goal of characterizing expression patterns relative to specific demographic populations (age, sex, fracture location) and healing patterns (normal versus delayed). This exploratory study investigated associations, providing pilot data for future studies, and was not associated *a priori* with a formal hypothesis. Analyses in this study were data-driven. This study is reported as per the Strengthening the Reporting of Observational Studies in Epidemiology (STROBE) guideline (S1 STROBE Checklist).

### Protein biomarker assessments

Serum and DBS samples were stored at −80 °C and −20 °C, respectively, until bulk assays were performed. All patient samples were run with a minimum of technical duplicates. Individual data points for the patients represent the average of the technical replicates.

#### Collagen X biomarker.

The CXM bioassay reliably measures circulating levels of the trimeric non-collagenous 1 domain of collagen X, first published as a real-time marker for bone growth velocity in children [30]. The method for performing the CXM enzyme-linked immunosorbent assay (ELISA) utilizing the SOMAmer capture reagent from SomaLogic has been published in detail for human [30] and murine [33] analysis from either serum or DBS. Repeatability and reliability of the assay were validated prior to initial use [30], and in this study, we further correlate DBS to serum. Inter-assay variability is controlled by measuring values for each sample in triplicate, and we control for intra-assay variability by completing two independent runs per sample. Peak CXM, time-to-peak CXM, and the change in CXM value (ΔCXM = CXM_peak_ − CXM_baseline_) were calculated from the longitudinal CXM data for all patients who had three or more visits.

#### Bone turnover biomarkers.

Immunoassays for bone metabolism biomarkers were obtained from ImmunoDiagnostic Systems (UK). Serum levels of C-terminal telopeptide of type I collagen (CTX, a bone resorption marker bone marker) was determined using an ELISA that has a sensitivity of 20 pg/ml, an intra-assay coefficient of variance of 8.15%, and an inter-assay coefficient of variance of 10.31%. The N-terminal propeptide of type I procollagen (P1NP, a bone formation marker) was measured by a radioimmunoassay with a sensitivity of 2.0 ng/ml, an intra-assay coefficient of variance of 4.14%, and an inter-assay coefficient of variance of 2.74%. These values were collected and analyzed as part of the primary VitaShock analysis as published [35] but incorporated into this study for secondary analysis with additional protein analytes.

#### Multiplex and ELISA protein analysis.

Patient’s serum samples were analyzed for sclerostin (SOST), parathyroid hormone (PTH), osteocalcin (OC), fibroblast growth factor 23 (FGF23), and leptin using the Human Bone Magnetic Bead Panel (EMD Millipore, Cat# HBNMAG-51K) according to manufacturer’s protocol. Analytes were detected using a Luminex 200 Instrument, and the Belysa Analysis Software Version 1.0.19 was used to quantify protein concentration per the manufacturer’s instructions and quality control standards. Samples were excluded from analysis if they failed Belysa quality controls standards established *a priori*. These include a bead count of less than 35, >20% variance between replicates, or if percent recovery was outside 70%–130%. ELISAs were performed for SOST (R&D Systems, Cat # DSST00) and growth differentiation factor 15 (GDF15, R&D Systems, Cat# QK957) according to the manufacturer’s recommended protocol.

### Radiographic scoring of fracture healing

This study includes data from patients with either isolated femur or tibia shaft fracture, and imaging included standard-of-care anterior-posterior and lateral radiographs obtained at routine outpatient clinical follow-up visits. Radiological healing was assessed by an independent blinded trauma-fellowship-trained orthopedic surgeon using the mRUST. The mRUST assesses radiological healing based on callus formation on four bone cortices on a scale from 4 (no callus) to 16 (remodeled). Studies report that these scales have reasonable reliability with reported intraclass correlation coefficients of 0.68–0.86 [13,35]. mRUST has also been validated for use in femur fractures [36].

### Clinical categorization of fracture healing

For this study, we adopted a functional definition of radiological healing as a mRUST score ≥12 based on prior literature demonstrating this score was a reliable predictor of union [37,38]. We then categorized the rate of healing as early (≤12 weeks), normal (13–26 weeks), or delayed (≥27 weeks) based on our prior publication [29]. In the prospective fracture study, we also stratified patient healing according to those demonstrating a mRUST ≥9 at 12 weeks as normal healing compared to those less than 9 as patients at risk for delayed healing. This breakpoint is based on evidence that this is the earliest time when mRUST can discriminate healing outcomes in patients [39].

### Statistics

All statistics were performed using SAS V 9.4 (SAS Institute, 2022) and R V 4.2.1 (R Core Team, 2022) using the *tidyverse* package [36]. Graphs were generated using GraphPad Prism v9.3.0 with data points representing individual patient samples. Effect sizes and confidence intervals (CIs) are listed on graphs and/or in tables in Supporting information.

Prior to analysis, all data were evaluated for distributional assumptions and missingness. CXM, CTX, P1NP, GDF15, leptin, and sclerostin were right-skewed; therefore, unless otherwise explicitly stated, all statistical analyses involving specific biomarkers and proteins were performed using log-transformed values. The level of significance was set at alpha = 0.05, and no adjustments were made for multiple comparisons due to the exploratory nature of the analysis and in order to remain sensitive to emerging patterns. Interpretation of uncorrected results should be made with caution since the type I error rate may be inflated. Monochrome correlation plots were used for biomarker correlations, and color plots were used for protein correlations.

For descriptive statistics, means with 95% CIs were calculated for normally distributed continuous variables, and medians with interquartile ranges (IQRs) were calculated for non-normally distributed variables. Counts with proportions were reported for categorical variables. For bivariate analyses, Pearson or Spearman correlation coefficients were used to determine the correlation between two continuous variables with 95% CIs. Independent *t*-tests or Wilcoxon Rank Sum tests were used to determine differences between two groups.

For the VitaShock samples, we fit linear mixed-effects models with a random intercept for patients to determine if healer status (early, normal, delayed) was significantly associated with changes in CXM levels over time. Time was included as a categorical indicator (0, 6, and 12 weeks). For the OHSU sample cohort, we also fit linear mixed effects models with a random intercept, but used natural cubic splines to model the effect of time since the change in CXM over time was non-linear. We also used continuous mRUST scores since healer status could not be determined for several participants with long gaps between visits (e.g., if a participant did not return for a follow-up visit until after 12 weeks, we could not determine at what point during the 12 weeks they had healed).

For the validation sample, we used Pearson correlations and paired t-tests to determine the extent of agreement between dried blood spot (DBS) and blood serum samples, and their stability over time. Linear regression was used to determine if DBS CXM values could predict serum values.

## Results

This study analyzed blood samples from a total of 264 participants from three distinct groups: (1) healthy participants without a fracture to serve as a control, (2) patient with a tibia or femur fracture that were enrolled as part of the VitaShock Study (NCT02786498), or (3) patients with a tibia or femur fracture that were part of a prospective clinical observational study. Full demographic information for each group is found in Table 1.

**Table 1:** Patient demographics and enrollment distributions.

	Unfractured/healthy	VitaShock secondary analysis	Prospective fracture
	(*n* = 111)	(*n* = 102)	(*n* = 51)
	*N* (%)	*N* (%)	*N* (%)
Female	55 (49.5%)	32 (31.4%)	22 (42.3%)
Male	56 (50.5%)	70 (68.6%)	29 (57.7%)
Tibia	n/a	41 (40.2%)	31 (61.5%)
Femur	n/a	61 (59.8%)	20 (38.5%)
Average age (range)	45.6 (21–85)	29.6 (18–50)	40.8 (19–83)

### CXM biomarker is stable in participants without fracture across age and between sexes

**Fig 1:**
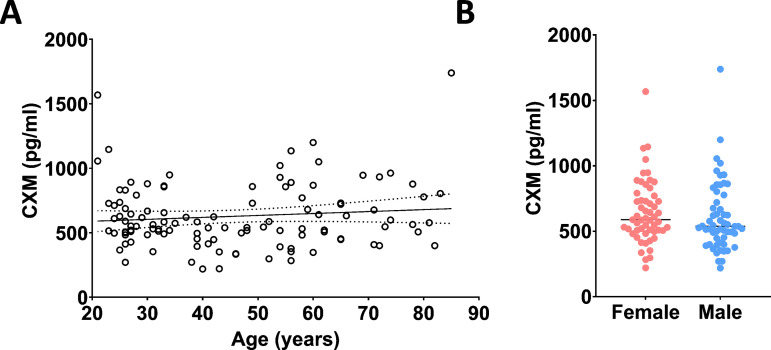
CXM levels do not correlate with age or sex in a non-fractured cohort. (A) The collagen X biomarker (CXM) concentration was plotted against ages ranging from 21 to 85 years with linear fit as a solid line and 95% confidence interval of best-fit line as a dotted line, no significant relationship was seen between age and CXM (CXM = 1.507 * age + 558.7, *r* = 0.04, *p* = 0.26). **(B)** Individual CXM concentrations for 55 females (red) and 56 males (blue) found no significant difference between sex and CXM level (*p* = 0.42) determined by a two-tailed *t* test. The line represents the median CXM value for female (median = 589.1 pg/mL, interquartile range [506.2, 771.1]) and male (median = 538.4 pg/mL, interquartile range [421.1, 863.6]).

Study participants without a fracture (*n* = 111) were recruited and enrolled between 2018 and 2020 as a control group to investigate the impact of age and sex on CXM concentration (S1 Data). Median expression across our age range of 21–85 years (mean 45.6 years; SD 18.1) was 563.4 pg/mL IQR [483.2, 771.1] and no significant relationship was discovered between CXM biomarker concentrations and age (*r* = 0.04, *p* = 0.26, Fig 1A). Within our uninjured control group, 55 were biologically female (median = 589.1 pg/mL, IQR [506.2, 771.1]) and 56 biologically male (median = 538.4 pg/mL, IQR [421.1, 863.6], Fig 1B). A two-tailed *t* test found no significant difference between sexes (*p* = 0.42).

### CXM biomarker has a strong association with canonical bone turnover markers

**Fig 2:**
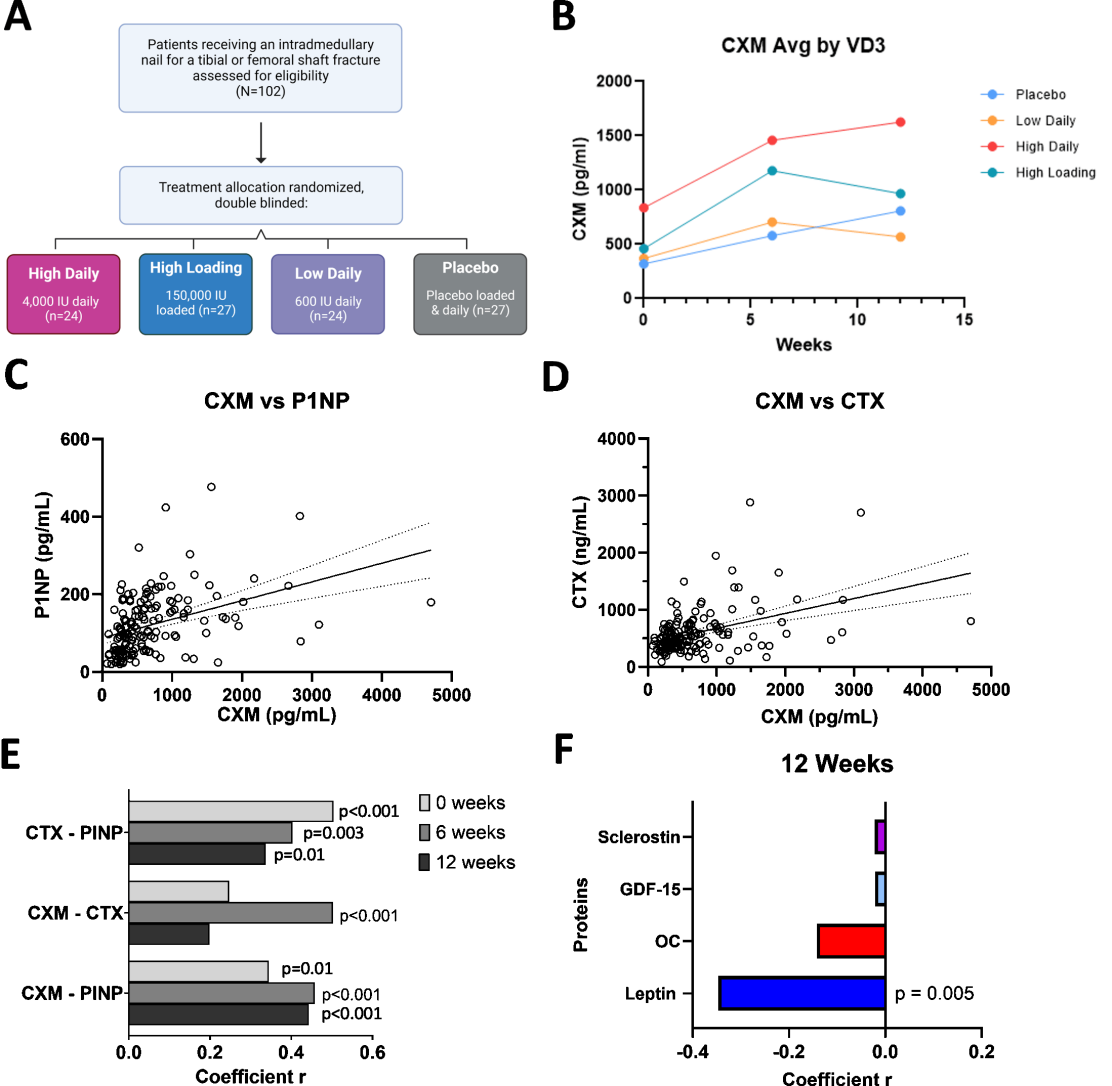
CXM Biomarker has a Strong Association with Canonical Bone Turnover Markers During Fracture Repair. (A) VitaShock (NCT02786498) participant flow diagram. IU, International Units (B) Collagen X biomarker (CXM) concentration plotted at 0, 6, and 12 weeks for the four different Vitamin D3 (VD3) treatment groups showed no significant difference in CXM between any treatment groups. **(C)** N-terminal propeptide of type I procollagen (P1NP) concentration plotted against CXM concentration with linear fit as a solid line and 95% confidence interval of best-fit line as a dotted line, significant relationship between P1NP and CXM (*r* = 0.50, *p* < 0.0001). **(D)** C-terminal telopeptide of type I collagen (CTX) concentration plotted against CXM concentration with linear fit as a solid line and 95% confidence interval of best-fit line as a dotted line, with a significant relationship between CTX and CXM (*r* = 0.40, *p* < 0.0001). Eighty-three percent of total data points collected shown in plots C and D, data points above 2,500 CXM pg/mL excluded to better show relationship, all data points at all timepoints were used in linear fit analysis (*n* = 159). **(E)** Pearson correlations between CTX, P1NP, and CXM at 0, 6, and 12 weeks (*n* = 53 points/biomarker/week; *p* values for each biomarker pairing according to week: CTX - PINP: 0 = <0.001, 6 = 0.003, 12 = 0.01; CXM - CTX: 0 = 0.08; 6 = <0.001; 12 = 0.15; CXM - PINP: 0 = 0.01; 6 = <0.001; 12 = < 0.001). **(F)** Pearson correlation coefficients between CXM and 4 proteins associated with bone formation at 12 weeks, significant negative correlation between CXM and Leptin (*p* = 0.005, *n* = 56–66 points/protein).

A secondary analysis was conducted on the serum saved from patients enrolled in the VitaShock study (NCT02786498, *n* = 102, Table 1 and S2 Data). CXM values at the time of injury confirmed no differences in CXM concentrations as a function of age (S1A Fig: age range 18−50, median age 28, *r* = −0.031, *p* = 0.44) or sex (S1B Fig: 31 female, 68 male, 3 missing CXM values at baseline; *p* = 0.16), and no statistical difference in the baseline CXM value between any of the study enrollment arms. Further, there was no significant difference in CXM response to Vitamin D3 treatment (VD3, Fig 2A and S1 Code) using a linear mixed model regression with a random intercept (Fig 2B). However, the ‘high loading’ (*p* = 0.06) and the ‘high daily’ (*p* = 0.09) groups approached a significant difference relative to placebo in the overall mixed model regression.

CXM did correlate with canonical bone turnover markers P1NP (*r* = 0.50, *p* < 0.0001, Figs 2C, S2A) and CTX (*r* = 0.40, *p* < 0.0001, Figs 2D, S2B). The strongest correlation occurred at 6 weeks for both P1NP (*r* = 0.46, *p* = 0.0006, Fig 2E) and CTX (*r* = 0.50, *p* < 0.0001, Fig 2E). We further assessed the relationship of CXM to additional proteins associated with fracture repair or aging: SOST, OC, GDF15, leptin, PTH, and FGF23. We found a significant negative correlation between leptin and CXM levels at 12 weeks after fracture (*r* = −0.34, *p* = 0.005, Fig 2F). No significant correlations were seen with the other proteins at 0 weeks, 6 weeks, or when all timepoints were clustered together (S3 Fig). We also measured PTH and FGF23, but both proteins were not detectable above our reliability threshold of 80%.

### CXM biomarker correlates with protein levels related to fracture healing

**Fig 3:**
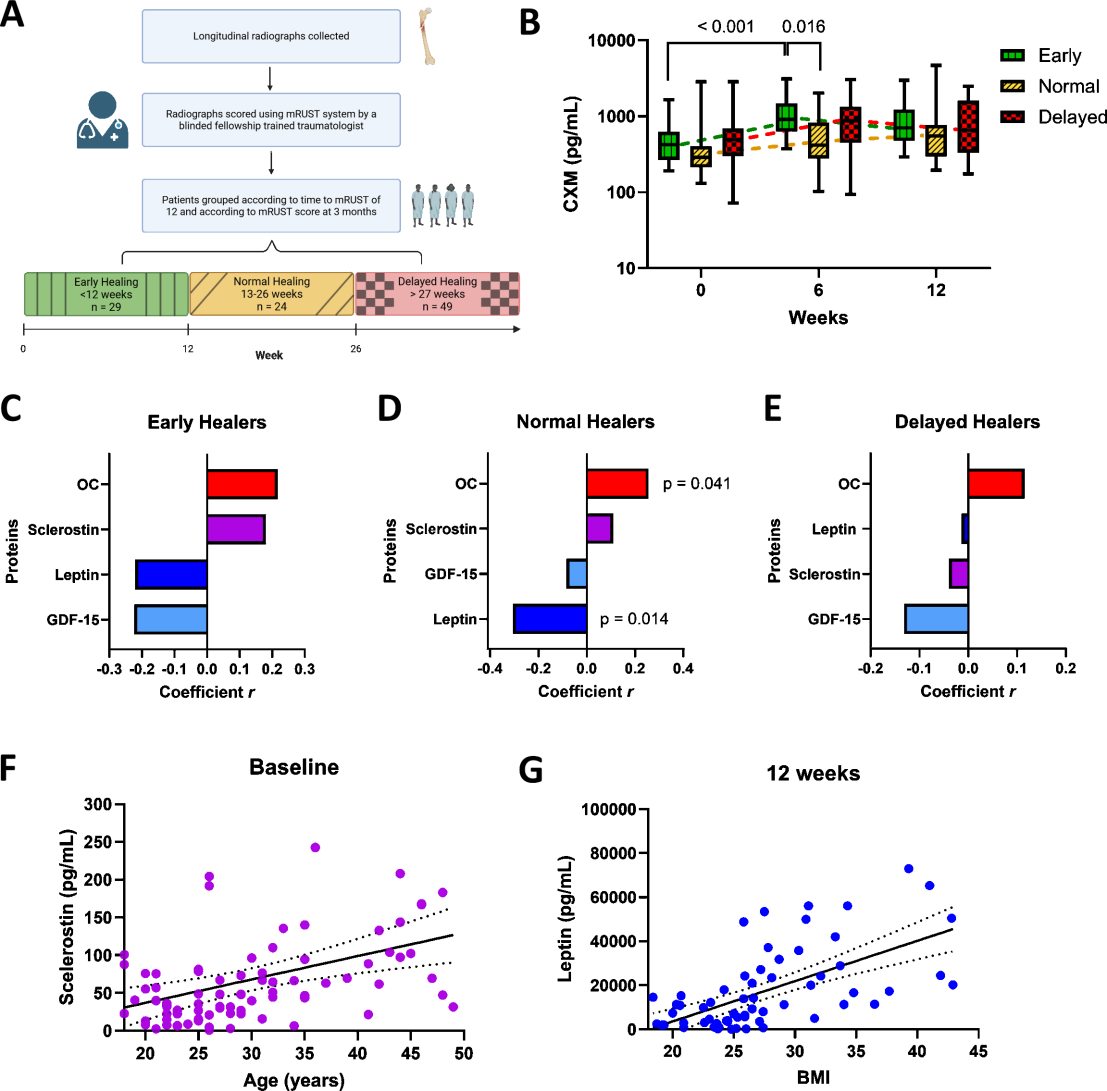
Protein analysis segregated by fracture healing performance. (A) Diagram of patient grouping according to time to radiological healing. mRUST, modified Radiographic Union Scale in Tibia. Created with BioRender.com. **(B)** Collegen X (CXM) concentration at 0, 6, and 12 weeks separated by early, normal, and delayed healers (early *n* = 27,22,25; normal *n* = 24,22,19; delayed *n* = 49, 34, 23 for 0, 6, 12 weeks respectively, sample size differences due to patient follow-up). The center line denotes the median value, the edges of the box denote the 25th and 75th quartile, and the upper and lower bounds go from min to max. **(C–E)** Pearson Correlations between CXM and 4 proteins associated with bone formation in (C) early healers (*n* = 72, 61, 73, and 74 for osteocalcin (OC), sclerostin, leptin, and growth and differentiation factor-15 (GDF-15), respectively), (D) normal healers (*n* = 64, 55, 65, and 64 for OC, sclerostin, GDF-15, and leptin, respectively), (E) and delayed healers (*n* = 99, 100, 89, and 98 for OC, leptin, sclerostin, and GDF-15, respectively) over 12 weeks. No significant correlations in early or delayed healers, a significant negative correlation in normal healers between CXM and leptin (*p* = 0.0138), and a significant positive correlation between CXM and OC (*p* = 0.0411). **(F)** Sclerostin concentration plotted against age, with a significant correlation between sclerostin and age (*r* = 0.46, *p* = 0.0007). **(G)** Leptin concentration plotted against body mass index (BMI), significant correlation seen between leptin and BMI (*r* = 0.63, *p* < 0.0001). Linear fit line in solid black and 95% confidence interval of best-fit line in dotted black line.

VitaShock patients were subsequently divided into functional healing categories as “early”, “normal”, or “delayed” based on time to reach a mRUST score ≥12 [29] (Fig 3A). Patients with early healing exhibited a peak in CXM at 6 weeks of 1,092 pg/mL (95% CI [804.8, 1,379]), which was significantly higher than both their baseline value of 506.6 pg/mL (95% CI [363.9, 649.3]; *p* < 0.001) and the peak value for patients with normal healing at 6 weeks (630.8 pg/mL, 95% CI [399.9, 861.8]; *p* = 0.016, Fig 3B). No significant difference was seen between CXM levels in the early healers compared to the delayed healers at any time point.

For patients with normal healing, CXM also shows a significant positive correlation with OC (*r* = 0.26, *p* = 0.0411) and negative correlation with leptin (*r* = −0.31, *p* = 0.0138, Fig 3D). Sclerostin transitioned from a positive correlation with CXM in early healers to a negative correlation in delayed healers and showed a significant correlation between increasing sclerostin levels with increasing age (*r* = 0.46, *p* = 0.0007, Fig 3F). Leptin negatively correlated with CXM in early and normal healers but not in delayed healers. Leptin levels also significantly correlated with body mass index (BMI) (*r* = 0.63, *p* < 0.0001, Fig 3G).

**Fig 4:**
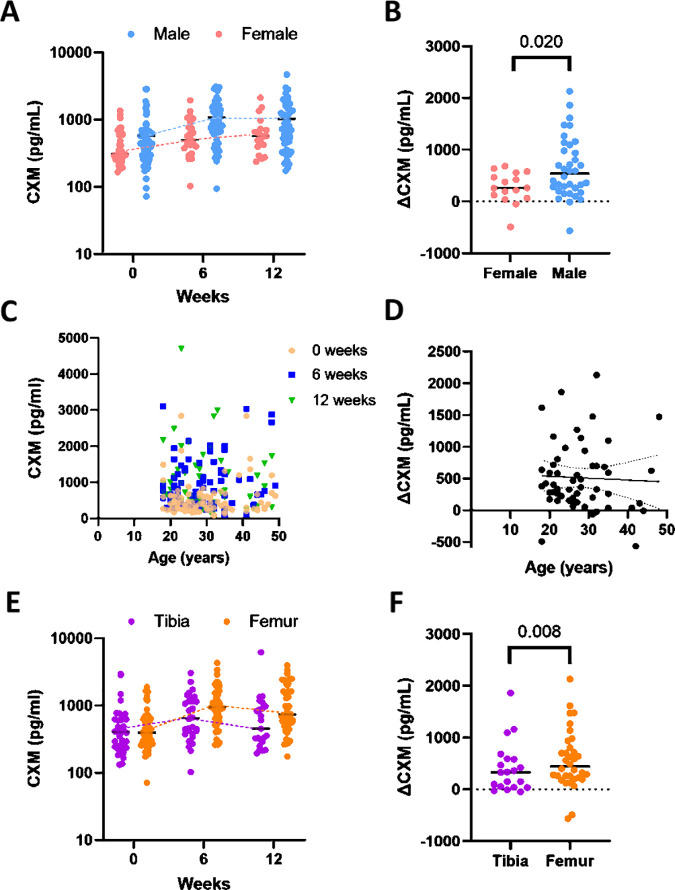
Patient-specific injury factors do not influence absolute but do impact the ∆CXM values. (A) Collagen X biomarker (CXM) concentration plotted for females (*n* = 32, red) and males (*n* = 70, blue) at 0, 6, and 12 weeks, no significant differences determined by a linear mixed model. (B) ∆CXM value (∆CXM, CXM_peak_ − CXM_baseline_) plotted for females and males, with a significant difference between sex and ∆CXM concentration (*p* = 0.020) determined by the Wilcoxon Rank Sum test. **(C)** All observed CXM values plotted against age with different weeks labeled by color and shape, no significant linear relationship between CXM and age at any time point (0 weeks: *r* = −0.04, *p* = 0.447; 6 weeks: *r* = 0.06, *p* = 0.130; 12 weeks: *r* = −0.14, *p* = 0.569). **(D)** ∆CXM values plotted against age with linear fit as a solid line and 95% confidence interval of best-fit line as a dotted line, no significant linear relationship between ∆CXM and age (*r* = −0.11, *p* = 0.759). **(E)** CXM concentration plotted for tibia (*n* = 41, purple) and femur (*n* = 61, orange) fractures at 0, 6, and 12 weeks, no significant difference determined by a linear mixed model. **(F)** ∆CXM values plotted for tibia and femur fractures, a significant difference between fracture location and ∆CXM (*p* = 0.008) determined by Wilcoxon Rank Sum test. Graphs represent individual data points; the black line is the median.

Patient sex, age, and fracture location did not have a significant direct impact on CXM levels. While male patients tended to have higher CXM values than females throughout the time course of healing, a linear mixed model analysis found this difference was not statistically significant (*p* = 0.072, Fig 4A). The change in CXM expression (∆CXM = CXM_peak_ − CXM_baseline_) was significantly higher in males (645 pg/mL (95% CI [446.6, 843.4]) than the females (271.8 pg/mL, 95% CI [111.0, 432.5]; *p* = 0.020, Fig 4B). Further, we find no age-association following fracture for either CXM (0 weeks: *r* = −0.04, *p* = 0.447; 6 weeks: *r* = 0.06, *p* = 0.130; 12 weeks: *r* = −0.14, *p* = 0.569, Fig 4C) or ∆CXM values (*r* = −0.11, *p* = 0.759, Fig 4D). The absolute CXM value in femur fractures was also not statistically different than in tibia fractures (*p* = 0.132, Fig 4E), but the ∆CXM value for femur fractures (444.9 pg/mL, 95% CI [217.6, 837.2]) was significantly higher than the ∆CXM value for tibia fractures (332 pg/mL, 95% CI [40.88, 589.6]; *p* = 0.008, Fig 4F).

### CXM can be reliably measured in serum or from dried blood spots (DBS)

**Fig 5:**
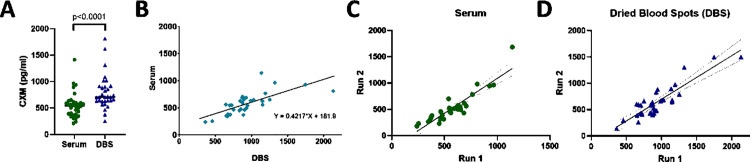
CXM can be reliably translated from venipuncture to finger prick. (A) Comparison of absolute collagen X biomarker (CXM) measurements from serum (*n* = 32, green) and dried blood spot (DBS, *n* = 32, blue) of matched patient samples using a two-tailed paired t test (*p* < 0.0001). (B) Comparison of CXM between serum and DBS of matched patient samples, a significant correlation was observed (*r* = 0.75, Serum = 0.4217 * DBS + 181.9, *p* < 0.0001). **(C)** Comparison of CXM measurements from serum at two time points (run 1 vs. run 2) approximately 1 year apart (*r* = 0.88, *p* < 0.0001). **(D)** Comparison of CXM measurements from DBS at two time points (run 1 vs. run 2) approximately 1 year apart (*r* = 0.80, *p* < 0.0001). Linear fit line in solid black and 95% confidence interval of best-fit line in dotted black line.

Next, we tested whether CXM could be measured from a DBS obtained by finger prick rather than full blood draw. In matched patient samples, CXM measurements from DBS were higher than that from serum (*p* < 0.0001, Fig 5A and S2 Code), but there was a strong correlation that can be mathematically equated (*r* = 0.75, Serum = 0.4217 * DBS + 181.9, *p* < 0.0001, Figs 5B and S4). We also examined the stability of samples over time, confirming that serum samples re-tested a year after capture demonstrated a high degree of correlation and stability in CXM values (*r* = 0.88, *p* < 0.0001, Fig 5C), with an average decrease in absolute serum CXM values of less than 10%. CXM levels measured from DBS were also highly correlated between measurements taken approximately 1 year apart (*r* = 0.80, *p* < 0.0001, Fig 5D), but there was a larger decrease in the absolute CXM level from DBS, ~25%, relative to the stability in serum.

### Longitudinal prospective study identifies CXM peaks during early endochondral phase of repair

**Fig 6:**
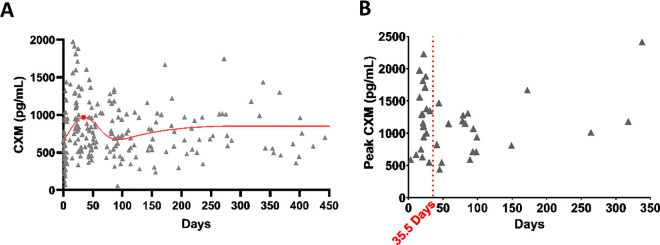
CXM peaks during the endochondral phase of healing. (A) Individual collagen X biomarker (CXM) levels over time with a cubic spline curve fit in red. Each triangle represents an individual patient value (*n* = 233). **(B)** Time-to-peak CXM value for individual patients (*n* = 42). The median time-to-peak CXM was 35.5 days, shown as the red dotted line. Outliers were identified using the Robust Regression and Outlier Removal (ROUT) with *Q* = 1% and removed in graph displays, but all outliers were used in data analysis.

With validation of DBS collection for CXM, we proceeded to prospectively enroll broadly for patients with an isolated tibia or femur fracture (Table 1 and S3 Data). Out of 12,072 patients sustaining fractures arriving through the OHSU trauma system between 2019 and 2023, 530 patients sustained fractures of the femur and tibia shafts. Here, we report on 51 consented patients sustaining eligible, isolatedfractures; 22 were biologically female, and 29 were biologically male, with an age range of 19–83 years (average age of 40.8 years, SD 19). DBS was collected at standard of care visits and patients with a minimum of three follow-up visits went on for further analysis: 42 patients achieved a minimum of three follow-up visits and 38 had baseline plus the three follow-up visits. A total of 233 samples were obtained and are plotted in a scatter plot showing that the highest CXM values fall between 20 and 40 days post-operatively with a peak inflection point in a loess curve at 30 days (Figs 6A and S5 and S3 Code). The peak CXM level for each patient is also plotted resulting in a median time-to-peak CXM at 35.5 days (Fig 6B). Elimination of outliers (*n* = 6) using ROUT (*Q* = 1%) moved the median time-to-peak CXM to 25.5 days.

### Relationship between endochondral healing and patient demographics

**Fig 7:**
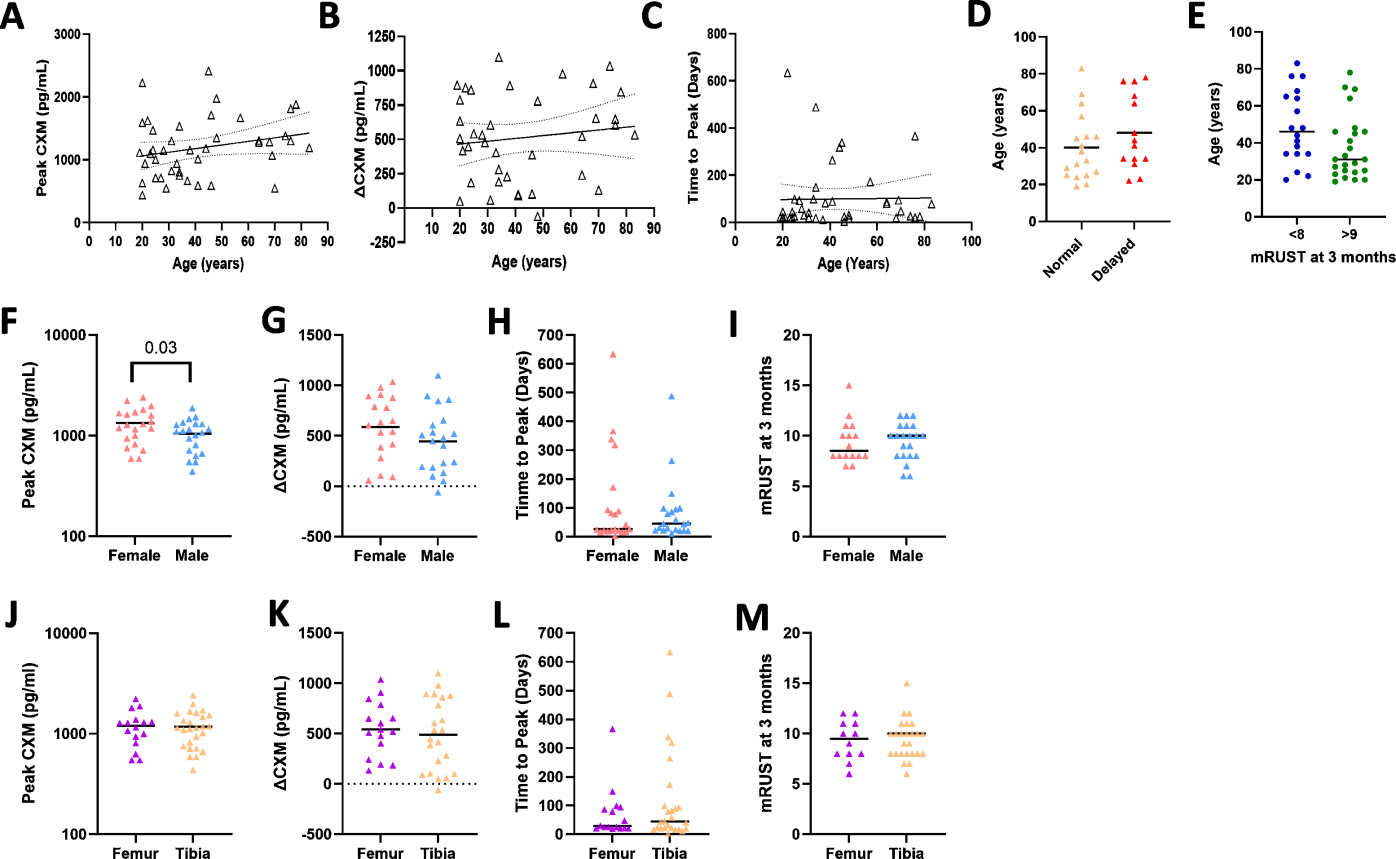
Relationship between endochondral healing and patient demographics. (A) Peak collagen X biomarker (CXM) concentration plotted against age for participants with ≥3 visits (*n* = 42). No significant linear relationship was seen (*r* = 0.24, *p* = 0.127). **(B)** ∆CXM concentration plotted against age for participants with ≥3 visits and baseline values (*n* = 38), no significant linear relationship was seen (*r* = 0.13, *p* = 0.432). **(C)** Time to peak CXM plotted against age for participants with ≥3 visits (*n* = 42), no significant linear relationship was seen (*r* = 0.17, *p* = 0.925). Linear fits as a solid line and 95% confidence interval of best-fit line as a dotted line. **(D)** Age for normal (*n* = 18, yellow) and delayed healers (*n* = 14, red), no significant difference determined by an unpaired two-tailed *t* test (*p* = 0.252). **(E)** Age for ≤8 at 3 months (*n* = 18, blue) and ≥9 at 3 months healers (*n* = 24, green), no significant difference (*p* = 0.055) determined by Wilcoxon Rank Sum test. mRUST, modified Radiographic Union Scale in Tibia. **(F)** Peak CXM concentration plotted for females (*n* = 21, red) and males (*n* = 21, blue) with ≥3 visits, significant difference (*p* = 0.03) determined by an unpaired two-tailed *t* test. **(G)** ∆CXM value (defined as: peak CXM value − baseline CXM) plotted for females (*n* = 20, red) and males (*n* = 18, blue) with ≥3 visits and baseline values, no significant difference (*p* = 0.177) determined by an unpaired two-tailed *t* test. **(H)** Time-to-peak CXM plotted for females (*n* = 21, red) and males (*n* = 21, blue) with ≥3 visits, no significant difference (*p* = 0.990) determined by Wilcoxon Rank Sum test. **(I)** mRUST scores for female vs. male patients at 3 months (*p* = 0.659). **(J)** Peak CXM concentration plotted for tibias (*n* = 26, purple) and femurs (*n* = 16, orange) with ≥3 visits, no significant difference (*p *= 0.920) determined by an unpaired two-tailed *t* test. **(K)** ∆CXM value plotted for tibias (*n* = 22, purple) and femurs (*n* = 16, orange) with ≥3 visits and baseline values, no significant difference (*p* = 0.624) determined by an unpaired two-tailed *t* test. **(L)** Time to Peak CXM plotted for tibias (*n* = 26, purple) and femurs (*n* = 16, orange) with ≥3 visits, no significant difference (*p* = 0.873) determined by Wilcoxon Rank Sum test. **(I)** mRUST scores for femur vs. tibia fractures at 3 months (*p* = 0.878).

For patients enrolled in the prospective observational study, we next tested whether peak CXM, ∆CXM, or time-to-peak CXM segregated according to age, sex, or fracture location. We found no significant direct relationship between peak CXM (*r* = 0.24, *p* = 0.127, Fig 7A), ∆CXM (*r* = 0.13, *p* = 0.432, Fig 7B), or time-to-peak CXM (*r* = 0.17, *p* = 0.925, Fig 7C) and age after long bone fracture. We further found no significant difference in the rate of bone healing with age using two different published stratifications of the mRUST score. First, in patients where a clinical determination of healing was reached (mRUST score of ≥12, *n* = 32), we used the same definition as above (Fig 3), where normal healing was defined as achieving this score between 13 and 26 weeks and delayed healing as ≥27 weeks (*p* = 0.25, Fig 7D) [29]. Secondarily, we used a newer system, which takes the mRUST score at 3 months and categorizes patients as at risk for delayed healing if their score was less than 9 at this time point (p = 0.055, Fig 7E) [39].

Unlike age which had no impact on CXM, we find that female patients in this prospective cohort demonstrated a significantly higher mean peak CXM value (1,331 pg/mL, 95% CI [1,093, 1,568]) compared to males (1,049 pg/mL, 95% CI [878.6, 1,219]; *p* = 0.03, Fig 7F). Further, using a linear mixed effects model, we establish that at any given time point, we can expect females to express ~25% higher CXM levels than males (*B* = 0.2309, SE = 0.114, *p* = 0.045). This did not lead to sex-specific differences in ∆CXM (*p* = 0.177, Fig 7G) or time-to-peak CXM (*p* = 0.704, Fig 7H), and mRUST scores at 3 months were not significantly different by sex (*p* = 0.659, Fig 7I). No significant difference in peak CXM, ∆CXM, time-to-peak CXM, or mRUST at 3 months was found between femurs and tibias was noted in this patient cohort (*p* = 0.920, *p* = 0.624, *p* = 0.873, *p* = 0.878 Fig 7J–7M).

## Discussion

The goal of this study was to investigate the concentration patterns of a biomarker to the endochondral stage of fracture repair in patients following fracture. We measured the circulating levels of the degradation product of the trimeric collagen X protein (CXM)—a provisional cartilage matrix protein that precedes fracture callus mineralization—comparing its expression patterns to established bone turnover markers and radiographic mRUST scores. Our findings demonstrate that this blood-based biomarker positively correlates with other markers of bone healing and that it may deliver earlier insights into the healing process than traditional metrics of bone healing with peak expression at approximately 26 days. Further, we validate that the CXM biomarker can be reliably transferred from a serum-based assay, requiring a phlebotomist to draw blood, to a simple finger-prick test, significantly improving the practicality of clinical implementation.

Clinical practice currently lacks quantitative standards to definitively track fracture healing progression. While the semi-quantitative mRUST radiographic scoring system has emerged as the clinical standard for monitoring long bone healing, its reliance on detecting mineralized bone makes it a late-stage indicator of healing. In practice, mRUST faces significant limitations: surgical hardware frequently obstructs clear visualization of fracture lines across all cortices, and it is only validated for tibial, femoral, and humeral shaft fractures [13,36,40]. Furthermore, the mRUST score achieves clinical meaning only when categorized by specific time points or cut-off thresholds previously correlated with functional healing outcomes. This is problematic as different studies have established different metrics of “healed” [37–39,41] and categorization of the continuous mRUST scale substantially diminishing the statistical power and nuanced insights that could be derived from utilizing its full scoring range. Blood based biomarkers have been proposed as a potential quantitative solution, but none have transferred into common clinical practice due to the relatively small size of the studies that have been done to date, non-specific expression of the proteins, and high degrees of variation with sex and age since the biomarkers have largely targeted bone turnover markers [26–28,42–44]. These constraints highlight the critical need for more versatile and earlier healing assessment methods.

This research establishes CXM as a promising biomarker for monitoring endochondral fracture healing. We demonstrate that a specific advantage of CXM as a biomarker is the low, but detectable, levels in healthy adults with no independent age or sex-specific associations that would confound analysis due to baseline variations. In patients with a fracture, our data supported the published conclusion of the VitaShock study (NCT02786498) showing that Vitamin D does not significantly accelerate fracture healing [29]. The high daily Vitamin D, followed by high Vitamin D loading, showed the highest average CXM values, but these were not statistically different than the low dose or placebo when assessed with a linear mixed model with random intercept. However, added sensitivity compared to bone turnover markers motivated the subsequent categorization of the VitaShock patients according to functional healing (early, normal, delayed) for correlation to CXM response [29]. Here we found that patients with early healing demonstrated a peak CXM value at 6 weeks post-operatively that was significantly higher than both their baseline value and the 6-week CXM value of patients with normal healing. We did not find a statistically significant difference in the CXM pattern between patients categorized as early and delayed healers according to the mRUST score, perhaps due to the limitation of the existing study time points (baseline, 6- and 12-weeks post-operatively). The VitaShock study was designed to capture the bone turnover markers prior to incorporation of CXM. As such, the sample schedule may have missed the best signal for an endochondral biomarker.

The positive association of elevated CXM values at 6 weeks in patients with early fracture healing motivated us to prospectively determine the time to peak CXM. We found that the median peak CXM concentration was at 35 days, approximately 1 week earlier than our 6-week time point in VitaShock and much earlier radiographic evidence of fracture healing (for normal healing we defined as ≥12 between 13 and 26 weeks). Peak CXM expression aligns with what could be expected as a window for soft-to-hard callus conversion, but has not been previously established. It is important to note that this median time did not discriminate patients by their healing status but rather included all prospectively enrolled tibial and femoral shaft fractures with a minimum of three follow-up visits (*n* = 42). Elimination of outliers (*n* = 6) moved the median time-to-peak CXM to 25 days.

While CXM was our primary biomarker of interest, we also show a positive correlation between CXM and the established biomarkers for bone formation (P1NP and OC) and bone resorption (CTX). We find the strongest association at 6 weeks post-operatively, concordant with the biological expression of collagen X at the inflection between cartilage turnover and bone formation [33]. CXM expression patterns support prior studies showing that elevated P1NP and CTX at 6 weeks were associated with early radiological healing at 12 weeks [29] or that patients with nonunions have lower expression of OC [45–47].

This study also examined the CXM response following fracture with respect to patient demographics. Protein analysis revealed a negative correlation between CXM and leptin levels. Additionally, leptin showed a positive association with patient BMI, aligning with established evidence that leptin serves as an obesity biomarker. These data suggest that leptin levels may be predictive of poor healing or that leptin is inhibitory of endochondral bone repair, further supporting clinical evidence that obesity is a risk factor for delayed or nonunion [42].

We found no independent variation in CXM in patients with or without fracture across the age span of 18–85. This is consistent with our previous data in a small cohort of tibial plateau patients, where we similarly found no correlation between age and CXM response [48]. Independent clinical studies also have not found a strong association between nonunion and age. A recent systematic review only reported a single clavicle fracture study with a meaningful odds ratio (1.3) between nonunion and age, with other larger meta-analyses reporting no association [49]. We previously have shown a significant correlation between aging, immunosenescence, and expression of bone inhibitors in the context of osteoarthritis [50], but did have not evaluated similar biomarkers in patients with fractures. An important limitation of our study is that it was a clinical observation study and was not designed or powered to detect the association of age with clinical nonunion, which is only estimated to occur in 2%–10% of long bone fractures [17,24]. This may be a limitation of many existing clinical trials as preclinical studies have demonstrated that decreased vascularization and a dysregulated immune system contribute to delayed fracture healing [51–54]. Our findings may not extend to older individuals with metabolic disease since the median age of the retrospective study was 30 (range: 18–50) and the prospective study was 41 (range: 19–83) years old.

Sex differences were mixed between our secondary retrospective and prospective studies. In the retrospective analysis of VitaShock patients, ∆CXM was higher in males (*n* = 70) relative to females (*n* = 32). This is consistent with preclinical and clinical studies suggesting healing is broadly more robust in males compared to females [55,56]. On the contrary, our prospective study found females (*n* = 22) to have ~25% higher CXM levels compared to males (*n* = 30) at any given time. However, this increased CXM was not correlated with an increased mRUST score at 3 months, suggesting that we are not seeing functional sex-specific changes. Interestingly, the data on whether sex is an independent risk factor for delayed or nonunion also remains inconclusive [49], but recent meta-analyses or larger prospective studies have found a negative correlations between sex and fracture healing capacity [57,58].

CXM response relative to fracture location was similarly not conclusive. The secondary analysis of the retrospective VitaShock patients found higher post-operative ∆CXM in femur fractures compared to tibia fractures, which would make sense as a larger fracture callus would be expected from this larger bone. However, significant increases in the femur-CXM response were not preserved in the smaller prospective cohort correlating with no difference in mRUST scores at 3 months. To our knowledge, no similar comparison of bone biomarkers response between tibia and femur has been done, so there is no precedent for expectation.

A limitation of our existing demographic analyses is that the sample sizes were not large enough to rigorously control for all potential covariates, such as race, age, and BMI (leptin levels) across sex and fracture location. We know these factors were not equivalent between our recruiting sites of Baltimore, Portland, and Vail. Additionally, the VitaShock study captured an uneven distribution of males (*n* = 70) to females (*n* = 32) and an uneven distribution of femurs (*n* = 61) to tibias (*n* = 41). This distribution reflects the nature of a trauma setting may have impacted the ability of the CXM data to reach significance. The prospective study was limited as our delayed healing population was not large enough to characterize when and to what magnitude a statistically meaningful break in the CXM peak or expression pattern may be to predict nonunion. Such analysis will require substantially larger cohorts with significant populations of delayed and nonunion. As such, this study was foundational in generating the preliminary data to determine study design and power *a priori* future studies that will validate the clinical utility of CXM as a fracture healing diagnostic.

Future multi-center trials will aim to provide more depth into whether CXM can be used to delineate if patient demographics or treatment-specific factors—such as open versus closed fractures, surgical versus non-surgical management, or fracture of other bones—influence bone healing. These data would be highly valuable as there are no current outcome metrics that provide definitive data on the impact of demographics on fracture healing [49]. In practice, these studies are very complicated and expensive, requiring large prospectively designed studies to adequately control for the heterogeneity of fracture repair. Further, standard fracture trial protocols fail to measure practical metrics of functional healing, including pain and mobility. Future studies incorporating these metrics with CXM may support earlier clinical decision-making.

Taken together, our study supports that the early measurement of the endochondral phase of fracture healing with CXM may have both diagnostic value and sensitivity to explore differences in the rate of fracture healing. The CXM biomarker can be measured in serum as part of a larger biomarker panel that could collect additional data on factors such as inflammation. Alternatively, we have also validated the reliability and stability of this biomarker when collected from a finger prick onto a DBS card, increasing the translational readiness of CXM and enabling this as an “at home kit” that could add diagnostic resolution/sampling between clinic visits. Integration of biomarkers into fracture healing outcomes helps to address shortcomings of the mRUST scoring system and provides as a fully quantitative metric that is independent of fracture visualization on radiographs. We believe CXM healing trajectories will ultimately be additive to clinical care by providing trajectories of the early healing process, but do not anticipate this will replace standard of care radiographs confirming bone mineralization, fracture reduction, and hardware integrity.

## Supporting information

S1 STROBE Checklist STrengthening the Reporting of OBservational studies in Epidemiology Checklist **—**Guidelines for reporting observational studies’ by the STROBE Initiative, available at https://www.strobe-statement.org, licensed under CC BY 4.0 Copyright 2025.
(DOCX)

S1 Fig CXM levels do not correlate with age or sex at baseline in a fractured cohort: **(A)** Collagen X (CXM) concentration plotted against ages ranging from 18 to 50 years with linear fit line as solid line and 95% confidence interval of best-fit line as a dotted line, no significant relationship was seen between age and CXM (*p* = 0.44). **(B)** CXM concentrations plotted for 68 males (blue) and 31 females (red), line represents median, no significant difference between Sex and CXM concentration (*p* = 0.16) determined by Wilcoxon Rank Sum test.
(GIF)

S2 Fig CXM vs. P1NP and CTX Correlations with all data points displayed **(A)** P1NP concentration plotted against collagen X (CXM) concentration (*n* = 159) with linear fit as a solid line and 95% confidence interval of best-fit line as a dotted line, significant relationship between P1NP and CXM (*r* = 0.50, *p* < 0.0001). **(B)** CTX concentration plotted against CXM concentration (*n* = 159) with linear fit as a solid line and 95% confidence interval as a dotted line, with a significant relationship between CTX and CXM (*r* = 0.40, *p* < 0.0001).
(TIFF)

S3 Fig CXM levels do not correlate with proteins at 0 weeks, 6 weeks, or overall : **(A–C)** Pearson Correlations between collagen X (CXM) and 4 proteins associated with bone formation at (A) 0 weeks (*n* = 95, 83, 97, 98 for OC, Sclerostin, leptin, GDF-15 respectively), (B) 6 weeks (*n* = 76, 66, 76, 76 for OC, sclerostin, GDF-15, and leptin respectively), and (C) overall (*n* = 235, 205, 237, and 237 for OC, leptin, sclerostin, and GDF-15, respectively). No significant relationship was seen between CXM and proteins at 0 weeks, 6 weeks, or overall. PTH and FGF23 were not shown due to too low of levels for analysis.
(GIF)

S4 Fig DBS and Serum Correlations **(A)** Comparison of collagen X (CXM) between observed serum and serum predicted by the equation in Fig 5B, a significant correlation was observed (*r* = 0.75, serum_predicted_ = 0.5149* serum_observed_ + 280.6, *p* < 0.0001). Linear fit line in solid black and 95% confidence interval of best-fit line in dotted black line. **(B)** Bland–Altman plot of dried blood spots (DBS) and serum. Ninety-five percent limits of agreement from −114.1 to 837.8.
(GIF)

S5 Fig CXM levels at all time points for categorized healers: **(A)** Collagen X (CXM) concentration plotted against days for all data collected for early/normal healers. **(B)** CXM concentration plotted against days for all data collected for delayed healers. This is a subset of patients from the prospective fracture patient cohort with three or more visits.
(GIF)

S1 Data Healthy donor master. Raw data from the healthy, uninjured volunteers that were recruited from the community through the Steadman Philippon Research Institute (SPRI) according to the Institutional Review Board (IRB) Approval obtained from Vail Health under protocol #2018-48. These participants serve as a control/baseline.
(PDF)

S2 Data VitaShock master. Raw data from a secondary analysis of serum samples from 102 patients with a fracture was collected as part of the VitaShock phase II exploratory randomized clinical trial comparing the effect of multiple vitamin D_3_ dosing strategies on fracture healing in patients with isolated lower extremity long bone fractures (NCT02786498). Approval for the study was obtained by the Hamilton Integrated Research Ethics Board (2017-1952) and the University of Maryland IRB (HP-00069705). A Material Transfer Agreement (MTA) was established for sample transfer between the University of Maryland and SPRI.
(PDF)

S3 Data Prospective fracture master. Raw data from dried blood spots collected from patients with a fracture that were recruited from the Oregon Health & Science University (OHSU) level 1 academic trauma center and The Steadman Clinic under independent, but aligned, IRB-approved protocols (OHSU #00019234: TSC #2018-48).
(PDF)

S1 Code VitaShock analysis SAS code. SAS code for the analysis of data from the VitaShock phase II exploratory randomized clinical trial for Figs 2–4 and S1–S3.
(RTF)

S2 Code DBS versus serum SAS code. SAS code for the analysis of repeatability and reliability data comparing CXM data from serum and dried blood spots. Data was displayed in Figs 5 and S4.
(RTF)

S3 Code Prospective data SAS code. SAS code for the analysis of data from the prospective fracture data in Figs 6, 7, and S5.
(RTF)

S1 Table Statistical tables for figures. Tables containing detailed statistical results from each figure.
(DOCX)

## Abbreviations

AIDs: acquired immunodeficiency syndrome
BMI: body mass index
CXM: breakdown product of collagen X protein
ELISA: enzyme-linked immunosorbent assay
FGF23: fibroblast growth factor 23
GDF15: growth differentiation factor 1
HIV: human immunodeficiency virus
IQR: interquartile range
IRB: Institutional Review Board
mRUST: modified RUST
MTA: Material Transfer Agreement
OC: osteocalcin
OHSU: Oregon Health & Science University
PTH: parathyroid hormone
ROUT: Robust Regression and Outlier Removal
RUST: Radiographic Union Score for Tibia
SD: standard deviation
SOST: sclerostin
STROBE: Strengthening the Reporting of Observational Studies in Epidemiology
