# Supplementary figures and images for: Efficacy of biomarkers in the endochondral phase of fracture repair and healing in long bones: A clinical observational studys
Source: PLoS Med. 2025 Aug 29;22(8):e1004640. doi: 10.1371/journal.pmed.1004640 (PMC12410876; doi:10.1371/journal.pmed.1004640)

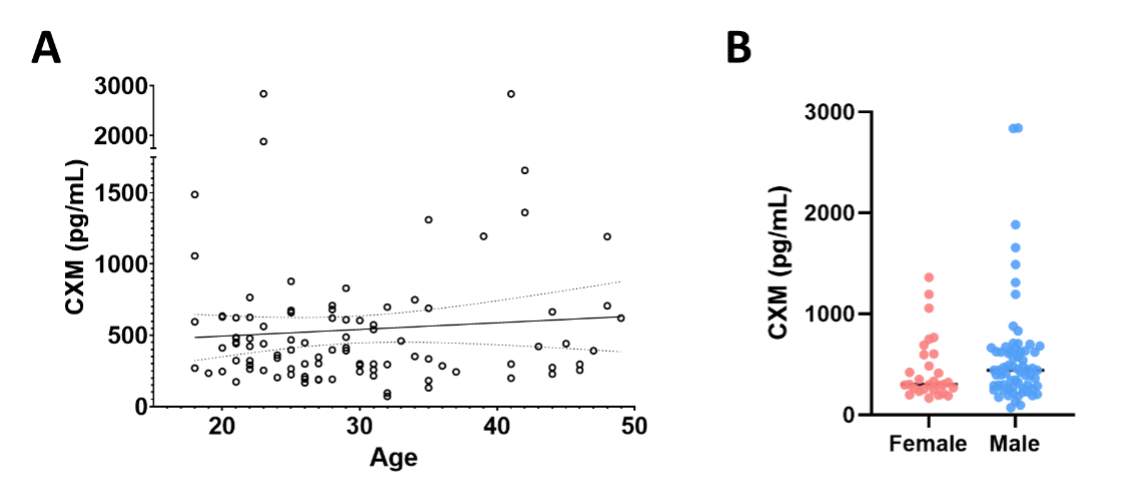

Supplement: S1 Fig — (A) Collagen X (CXM) concentration plotted against ages ranging from 18 to 50 years with linear fit line as solid line and 95% confidence interval of best-fit line as a dotted line, no significant relationship was seen between age and CXM (p = 0.44). (B) CXM concentrations plotted for 68 males (blue) and 31 females (red), line represents median, no significant difference between Sex and CXM concentration (p = 0.16) determined by Wilcoxon Rank Sum test. (GIF) [file pmed.1004640.s002.gif]

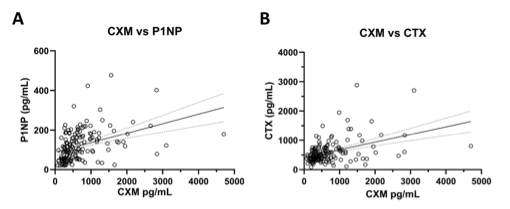

Supplement: S2 Fig — (A) P1NP concentration plotted against collagen X (CXM) concentration (n = 159) with linear fit as a solid line and 95% confidence interval of best-fit line as a dotted line, significant relationship between P1NP and CXM (r = 0.50, p < 0.0001). (B) CTX concentration plotted against CXM concentration (n = 159) with linear fit as a solid line and 95% confidence interval as a dotted line, with a significant relationship between CTX and CXM (r = 0.40, p < 0.0001). (TIFF) [file pmed.1004640.s003.tiff]

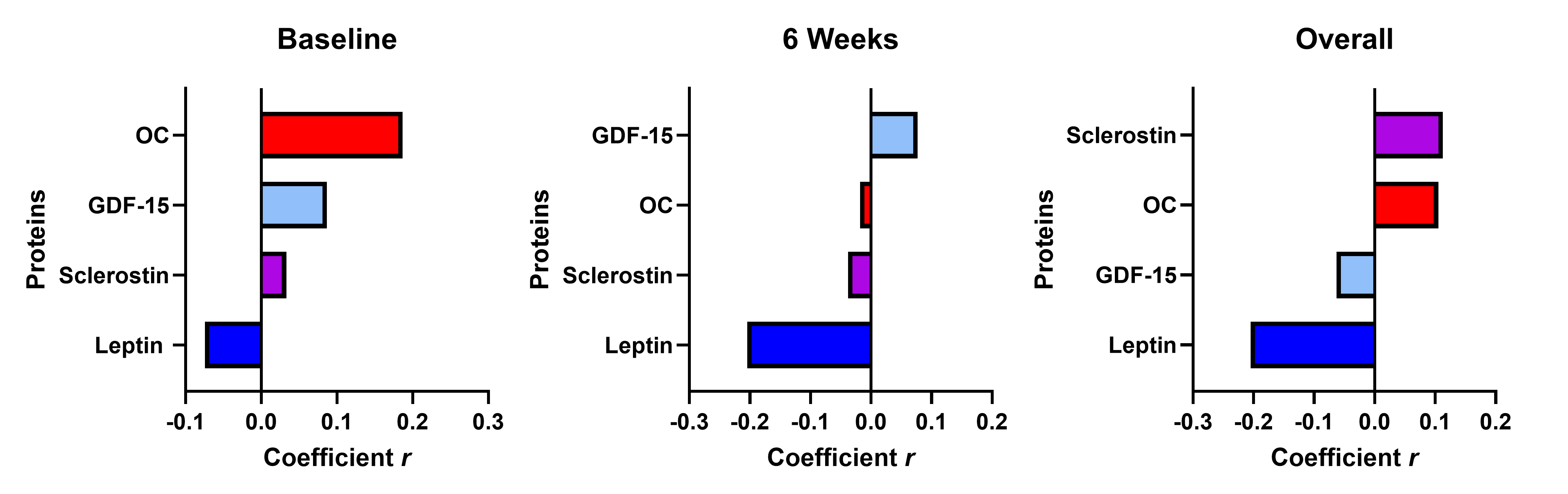

Supplement: S3 Fig — : (A–C) Pearson Correlations between collagen X (CXM) and 4 proteins associated with bone formation at (A) 0 weeks (n = 95, 83, 97, 98 for OC, Sclerostin, leptin, GDF-15 respectively), (B) 6 weeks (n = 76, 66, 76, 76 for OC, sclerostin, GDF-15, and leptin respectively), and (C) overall (n = 235, 205, 237, and 237 for OC, leptin, sclerostin, and GDF-15, respectively). No significant relationship was seen between CXM and proteins at 0 weeks, 6 weeks, or overall. PTH and FGF23 were not shown due to too low of levels for analysis. (GIF) [file pmed.1004640.s004.gif]

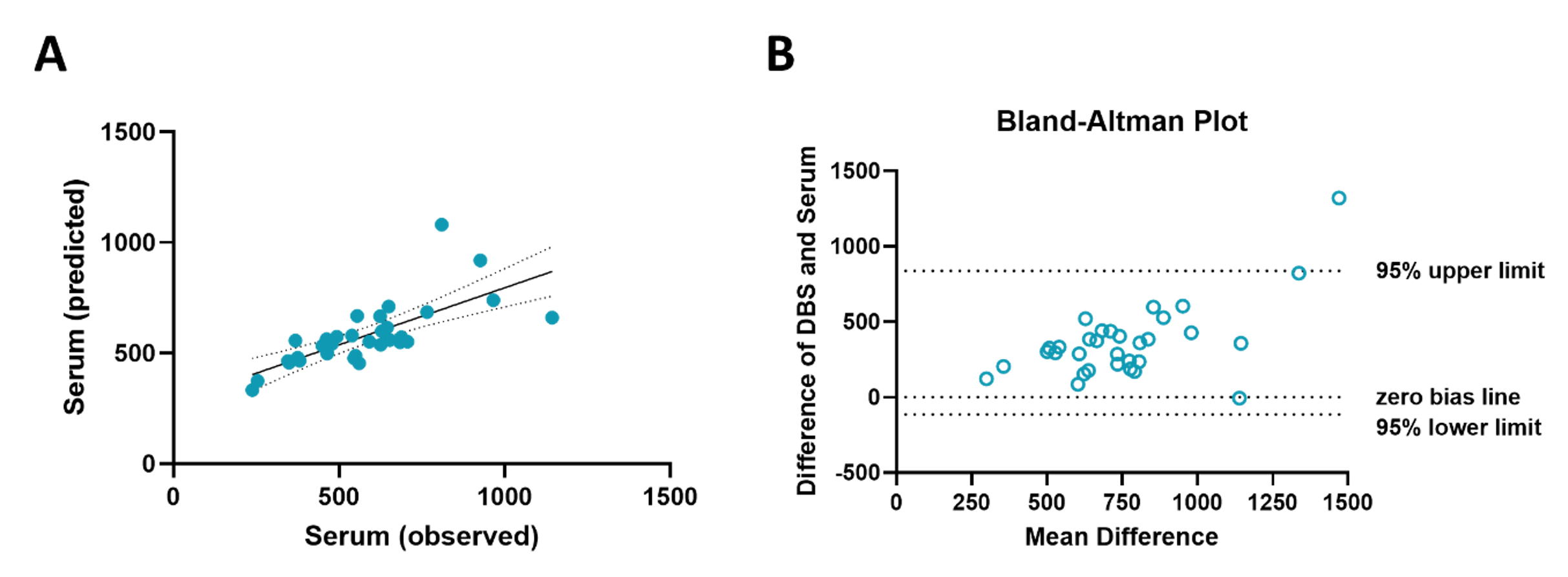

Supplement: S4 Fig — (A) Comparison of collagen X (CXM) between observed serum and serum predicted by the equation in Fig 5B, a significant correlation was observed (r = 0.75, serumpredicted = 0.5149* serumobserved + 280.6, p < 0.0001). Linear fit line in solid black and 95% confidence interval of best-fit line in dotted black line. (B) Bland–Altman plot of dried blood spots (DBS) and serum. Ninety-five percent limits of agreement from −114.1 to 837.8. (GIF) [file pmed.1004640.s005.gif]

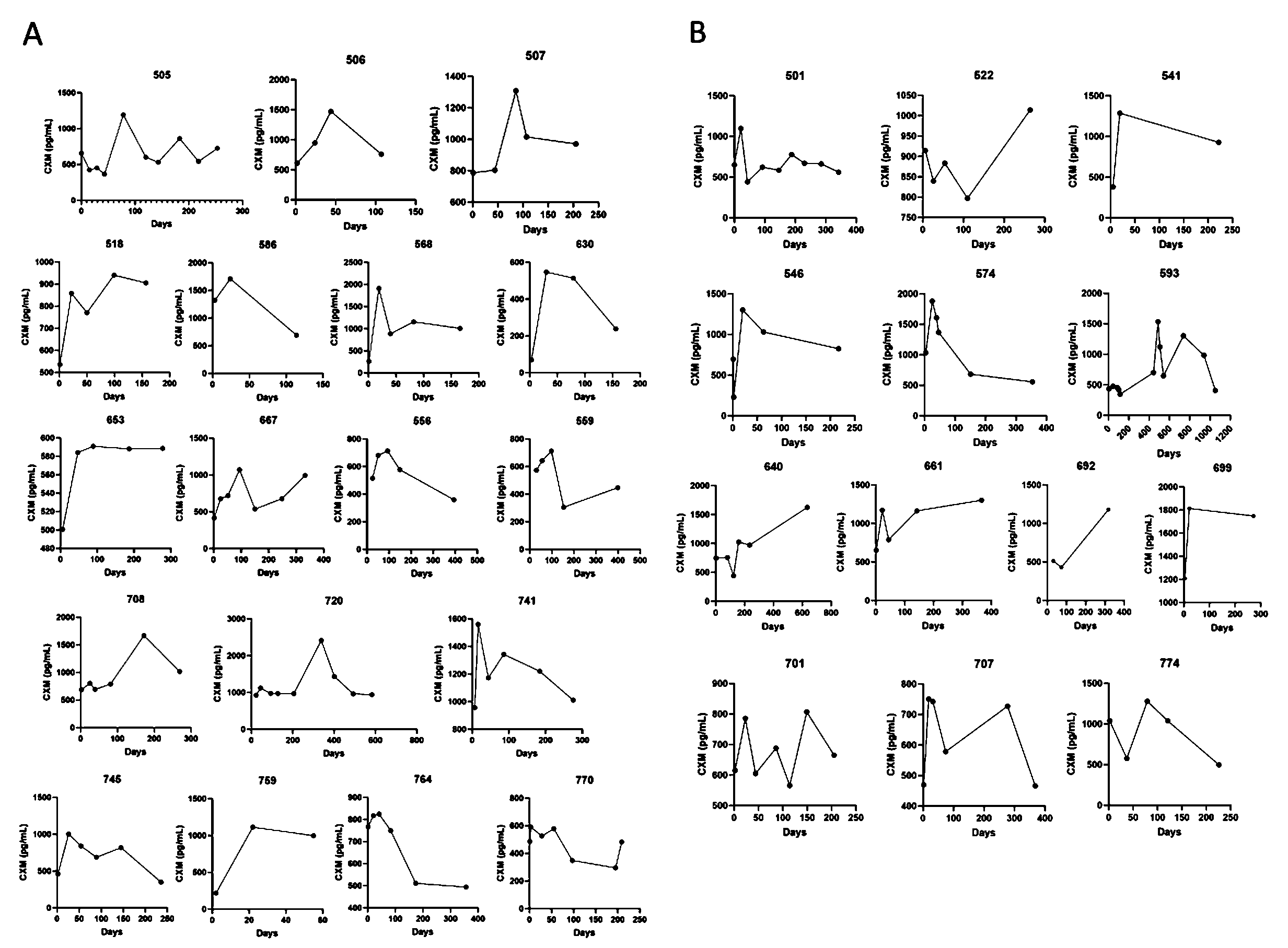

Supplement: S5 Fig — (A) Collagen X (CXM) concentration plotted against days for all data collected for early/normal healers. (B) CXM concentration plotted against days for all data collected for delayed healers. This is a subset of patients from the prospective fracture patient cohort with three or more visits. (GIF) [file pmed.1004640.s006.gif]
