# Supplementary material for: Efficacy of biomarkers in the endochondral phase of fracture repair and healing in long bones: A clinical observational studys
Source: PLoS Med. 2025 Aug 29;22(8):e1004640. doi: 10.1371/journal.pmed.1004640 (PMC12410876; doi:10.1371/journal.pmed.1004640)
Supplement: S1 Data — Raw data from the healthy, uninjured volunteers that were recruited from the community through the Steadman Philippon Research Institute (SPRI) according to the Institutional Review Board (IRB) Approval obtained from Vail Health under protocol #2018-48. These participants serve as a control/baseline. (PDF) [file pmed.1004640.s007.pdf]

Age	CXM	Sex
40	219	M
43	220.8	F
26	271	M
38	271.5	M
56	283.8	F
52	297.8	F
46	333.8	M
46	337.1	F
60	347.7	M
43	351.4	M
31	353.2	F
56	353.5	M
25	366	M
56	377.8	M
54	390.7	M
39	394.4	M
82	399.9	M
72	400	M
71	408.2	F
26	412.3	M
41	414.3	F
27	427.8	F
42	446.6	F
65	447.3	M
39	452.5	M
65	453.3	F
58	482	F
26	483.2	F
30	484.7	M
33	491.5	M
39	495.7	M
24	496.5	M
47	498.6	F
26	500.2	M
26	506.2	F
79	506.7	F
62	506.9	M
27	509.9	F
32	512.4	F
23	514.4	M
48	516.8	M
54	517	M
34	518.1	F
27	520.4	M
62	520.8	F
32	527.2	M

31	530.3	F
58	531.2	F
41	538.2	M
44	538.5	M
48	540.2	M
51	544.3	M
73	546.5	F
28	550	F
27	552.5	M
31	563.4	M
78	563.8	F
35	573.2	F
81	577.7	M
33	583.5	M
39	584.1	F
52	587	F
26	589.1	F
74	599.3	F
24	610.4	F
29	617.2	F
37	620.2	M
42	623.9	M
25	627.3	M
66	631.6	M
61	640.1	F
27	644.6	F
33	655.4	F
30	667.9	M
71	676.5	M
59	680.5	F
26	687.6	F
24	711.5	F
65	721	M
23	727.6	F
49	728.7	F
65	730.4	F
25	735.8	F
58	771.1	F
80	777.8	M
28	791.7	F
83	804.1	M
26	830.9	F
25	833.2	M
33	856	M
55	856.8	M
49	858.1	F
33	863	M

60	868.1	M
78	877.5	F
30	879.6	F
56	889.6	F
27	892.2	F
54	929	M
72	932.7	M
69	945.9	F
34	948.3	F
74	962.7	M
54	1020.9	M
61	1049.6	F
21	1055.8	M
56	1134.6	F
23	1145.7	F
60	1199.4	M
21	1568.1	F
85	1739	M
