# Supplementary material for: Efficacy of biomarkers in the endochondral phase of fracture repair and healing in long bones: A clinical observational studys
Source: PLoS Med. 2025 Aug 29;22(8):e1004640. doi: 10.1371/journal.pmed.1004640 (PMC12410876; doi:10.1371/journal.pmed.1004640)
Supplement: S2 Data — Raw data from a secondary analysis of serum samples from 102 patients with a fracture was collected as part of the VitaShock phase II exploratory randomized clinical trial comparing the effect of multiple vitamin D3 dosing strategies on fracture healing in patients with isolated lower extremity long bone fractures (NCT02786498). Approval for the study was obtained by the Hamilton Integrated Research Ethics Board (2017-1952) and the University of Maryland IRB (HP-00069705). A Material Transfer Agreement (MTA) was established for sample transfer between the University of Maryland and SPRI. (PDF) [file pmed.1004640.s008.pdf]

sample id	sex	age (years)	BMI	gustilo grade	vd3 group	tib/fem	weeks	CXM Level (Replicates, pg/mL)				CXM (pg/mL)	peak week (after baseline)	peak CXM (pg/mL)	dCXM (pg/mL)	CTX (ng/mL)	P1NP (pg/mL)	mRUST healer	mrust	mRUST class at 3 months	GDF-15 (pg/mL)	Leptin (pg/mL)	OsteoCalcin (pg/mL)	Sclerostin (pg/mL)	PTH (pg/mL)	FGF-23 (pg/mL)
							0	182.7225	184.2075	272.1375	260.64	224.926875				0.26	30.596				162.3478263			78.7		
							6	484.7625	373.59	342.6975	311.58	378.1575				0.32	88.416		6		105.4285757	4,223.8	5,006.2	28.0	-	-
							12	395.3925	337.005	309.24	284.2875	331.48125				0.34	89.234		8		99.52380608	3,526.1	6,546.8	44.5	-	-
VS1	male	25	24.5	3	A	tibia	26						6	378.1575	153.2306			normal	15	<8						
							38												16							
							52												16							
							0	546.885	607.725	754.2	750.915	664.93125				0.35	51.413				919.5652336	10285.37	7002.02	143.8	97.1	73.9
							6	580.815	655.8525	737.2125		657.96				0.64	102.73		7		389.6190516	13,430.2	13,859.4		52.98	170.08
							12	440.145	385.29	180.315		335.25				0.56	116.64		8			11,367.7	11,586.8	93.7		
VS2	male	44	36.5	3	D	tibia	26						6	657.96	-6.97125			delayed	10	<8						
							38												11							
							52												15							
							0	710.62	720.2	572.86	636.38	660.015									394.4347854	4915.46	4482.38	81.3	57.5	18.0
							6												5							
							12	2511.1	2271.48	1914.86	1768.64	2116.52							10		222.0000049	4,603.5	9,800.0	38.4	26.26	92.19
VS3	female	25	23	3	C	femur	26											delayed	9	>9						
							38												16							
							52												16							
							0	193.545	189.36	169.11	179.73	182.93625				0.34	48.789				1112.476198	696.60	15165.18	140.0	-	-
							6	186.525	158.625	23.9625	5.67	93.695625				0.51	97.34		6		427.7142818	693.5	13,858.9	168.7	-	-
							12	239.6475	238.59	246.1725	150.66	218.7675				0.67	117.15		7		564.5714281	804.7	12,102.4	126.4	-	-
VS4	male	35	20.9	3	C	tibia	26						12	218.7675	35.83125			delayed	14	<8						
							38												16							
							52												16							
							0	1198.013	1258.808	1441.328	1345.86	1311.00225				0.28	33.501				1207.809513	589.71	7061.32	94.5	25.6	-
							6	1837.328	1797.66	2238.638	1739.43	1903.264				1.65	139.53		5		517.1428769	1,291.2	25,397.5	201.4	-	-
							12	187.5825	183.9375	229.6125	174.8025	193.98375				0.49	56.67		9		746.1904705	24,206.5	96,125.9	46.3	7.65	54.65
VS5	male	35	25.9	1	C	tibia	26						6	1903.264	592.2618			normal	12	>9						
							38												16							
							52												16							
							0	320.7375	379.62	444.645	423.9675	392.2425				0.6	66.08				586.6666428	498.19	3135.30	48.1	6.2	101.6
							6	460.3275	453.69	528.6825	327.7575	442.614375				0.86	89.195		5		323.142857	2,502.5	9,131.9	71.4	-	93.77
							12	269.5275	280.17		196.2675	248.655				0.65	112.86		5		183.0476174	3,011.5	14,742.6	42.9	-	87.40
VS6	male	29	22.6	2	B	tibia	26						6	442.6144	50.37187			delayed	6	<8						
							38												13							
							52												16							
							0	784.08	703.38	537.18	514.52	634.79									775.1428591	9249.76	7644.54	55.4	106.4	92.1
							6												8							
							12												12							
VS7	male	20	28.4	3	A	femur	26											early	9	>9						
							38												16							
							52												16							
							0	830.5875	893.0475	1280.948	1223.258	1056.96025				0.69	90.039				2432.285725	1221.16	7520.22	22.9	-	-
							6	594.72		570.5325	527.6025	564.285				0.74	93.745		8		427.3333513	3,524.4	7,966.3	5.4	-	-
							12	618.075	619.785	524.745	497.6775	565.070625				0.44	73.107		12		213.9999972	2,421.5	9,776.4	-	-	-
VS8	female	18	18.7	3	B	femur	26						12	565.0706	-491.8896			early	16	>9						
							38												16							
							52												16							
							0	333.8325	347.265	491.445	521.0775	423.405				0.79	42.491				1652.761933	1174.51	14369.47	32.0	33.7	-
							6	1314.495		1333.643	1033.493	1227.21033				1.69	184.78		10		471.4285837	1,535.3	29,603.2	16.9	-	-
							12	633.4875	690.1425	815.175	624.375	690.795				0.97	194.43		13		315.9999925	2,845.9	52,383.0	2.4	-	-
VS9	male	22	20.9	3	D	femur	26						6	1227.21	803.8053			early	16	>9						
							38												16							
							52												16							
							0	155.295	170.5275	266.13	269.775	215.431875				0.54	31.668				1492.380933	1074.79	3190.06	76.5	37.2	-

							6	445.95	415.395	445.8825	358.65	416.469375				1.09	127.78		5		459.7142842	709.1	9,860.1	63.1	-	37.24
							12	240.705	291.375	323.145	333.135	297.09				0.63	188.93		8		386.8571427	684.0	15,931.9	25.4	6.43	45.24
VS10	male	31	27.4	3	D	femur	26						6	416.4694	201.0375			normal	15	<8						
							38												16							
							52												16							
							0		179.7975	33.9975	71.6175	95.1375				0.45	43.103				482.5714166	307.38	7785.61	51.2	16.2	-
							6	367.65	340.155	498.645	493.5825	425.008125				0.94	105.48		6		365.1428584	264.1	21,261.5	91.0	-	49.33
							12	318.96	354.5775	266.535	380.16	330.058125				0.5	69.61		7		241.7142838	759.3	11,118.9	37.3	-	45.38
VS11	male	32	23.5	1	D	tibia	26						6	425.0081	329.8706			delayed	9	<8						
							38												16							
							52												16							
							0	323.7975	260.2575	324.6975	298.755	301.876875				0.28	26.848				539.0476122	66276.61	1580.73	48.3		
							6	339.885	322.83	514.935	535.7925	428.360625				0.45	81.927		8		272.6666636	59,925.3	3,724.6	64.3		
							12	477.81	471.42	433.0125	304.9875	421.8075				0.345	82.215		8		214.0952369	65,296.5	5,681.7	30.3		
VS12	female	27	41	3	B	femur	26						6	428.3606	126.4838			delayed	11	<8						
							38												12							
							52												15							
							0	160.605	153.3825	215.5725	235.5975	191.289375				0.51	41.526				709.2380885	418.97	9380.81	67.1	51.0	-
							6	257.3325	270.0675	565.2	394.7625	371.840625				0.45	96.654		8		292.5714218	784.4	10,256.7	76.4	-	14.91
							12	1700.078	1784.025	1256.31	1098.36	1459.69325				0.34	131.86		12		173.2380978	1,982.8	10,745.4	34.5	-	31.68
VS13	male	27	19.3	3	A	femur	26						12	1459.693	1268.404			early	9	>9						
							38												16							
							52												16							
							0	1337.44	1267.8	1072.96	1100.42	1194.655									254.1904725	53560.47	6189.75	69.6		
							6	1048.62	1089.44	889.44	1250.44	1069.485							8		354.3809559	20,292.9	6,386.6	31.2		
							12												7							
VS14	female	39	31.6	3	B	tibia	26											delayed	9	<8						
							38												16							
							52												16							
							0	186.165	192.8025	280.755	278.415	234.534375				0.75	48.385				500.8571282	3866.34	6133.15	40.7	9.2	-
							6	407.7	331.335	214.4925	224.73	294.564375				0.72	150.7		4		297.6190554	4,771.1	15,649.5	46.1	-	30.23
							12	816.93	664.2	479.88	668.61	657.405				0.83	105.97		6		242.6666666	2,457.3	22,236.8	94.5	1.08	27.26
VS15	female	19	23.6	3	D	tibia	26						12	657.405	422.8706			delayed	9	<8						
							38												16							
							52												16							
							0	150.39	153.225	167.1075	55.2825	131.50125				0.31	20.301				1104.666627	2596.51	830.65	46.5	50.2	41.5
							6	869.13	1067.985	1439.573	1533.218	1227.4765				1.01	160.98		4		526.0952391	3,468.6	2,566.7	8.7	-	21.45
							12	872.685	604.9575		825.1425	767.595				0.58	45.155		6		257.8095247	3,945.1	3,525.6	-	-	-
VS16	male	35	24.5	3	C	tibia	26						6	1227.477	1095.975			normal	12	<8						
							38												16							
							52												16							
							0	223.875	265.6575	337.14	353.7225	295.09875				0.33	44.341				713.99998	20660.94	19616.94	110.2	71.0	82.0
							6	330.795	271.665	248.985	225.945	269.3475				0.465	111.475		8		811.4444348	17,253.0	32,605.0	109.8	41.14	129.40
							12	269.5275	200.3175		194.1975	221.3475				0.425	85.527		10		701.2222011	20,013.4	30,768.9	73.0	41.98	118.30
VS17	male	32	31.3	2	A	tibia	26						6	269.3475	-25.75125			normal	11	>9						
							38												14							
							52												16							
							0	161.0775	154.35	246.555	236.385	199.591875				0.089207	25.353				561.2222154	36392.67	6480.23	-	7.1	33.1
							6	91.0125	84.24		132.57	102.6075				0.21	46.355		4		368.0000007	42,831.8	11,605.1	60.4	-	40.01
							12	137.5875	120.5325	347.6475	343.4625	237.3075				0.225	55.025		11		513.6666496	49,974.3	9,371.7	41.7	-	41.36
VS18	female	41	30.9	3	B	tibia	26						12	237.3075	37.71563			normal	16	>9						
							38												16							
							52												16							
							0	709.54	753.5	700.24	673.14	709.105									1434.66666	21715.55	8853.45	-	3.7	-
							6												5						-	-
							12	1486.32	1457.72	1261.82	1260.38	1366.56							7		364.1111122	9,290.6	13,962.2	-	-	16.76
VS19	male	28	26.5	2	C	tibia	26											delayed	11	<8						

							38												14							
							52												16							
							0	342.88	311.02	299.46	213.84	291.8									869.7777787	57650.87	5104.42	15.0	31.0	-
							6	564.24	550.5	433.68	364.28	478.175							5		536.7777546	25,500.4	8,553.2	-	-	-
							12												10							
VS20	female	22	24.7	3	A	tibia	26											normal	14	>9						
							38												16							
							52												16							
							0	472.7925	591.93	537.7275	309.105	477.88875				0.295	60.625				390.0000088	44778.55	15605.96	-	3.5	-
							6	614.79	677.9025	969.2325	985.95	811.96875				0.85	138.565		9		204.9999899	10,052.8	17,787.8	-	-	16.65
							12	595.5525	653.3325	620.4825	546.885	604.063125				0.77	134.985		13		418.6666475	15,280.2	30,521.5	-	-	-
VS21	male	22	20.7	3	B	tibia	26						6	811.9688	334.08			early	16	>9						
							38												16							
							52												16							
							0	726.9975	562.1175	520.74	351.9675	540.455625				0.66	90.01				1248.222205	84.02	20900.34	-		
							6	1707.323	1834.133	2263.838	2265.098	2017.598				0.58	180.23		8		707.7777849	1,283.2	17,590.2	-	-	19.80
							12	2146.793	2157.008	1664.1	1839.443	1951.836				0.785	118.145		11		834.9999917	180.9	29,433.6	-	-	-
VS22	male	31	23.7	3	C	femur	26						6	2017.598	1477.142			normal	15	>9						
							38												16							
							52												16							
							0	424.2	368.16	457.56	338.08	397									2528.222283	315.61	26767.66	15.6	20.8	-
							6	2474.24	2361.58	1946.86	1811.06	2148.435							8		982.444432	213.4	17,392.2	-	-	-
							12												12							
VS23	male	25	23.4	1	A	femur	26											early	9	>9						
							38												16							
							52												16							
							0	668.48	764.18	521.62	528.32	620.65									1504.333297	29769.21	14311.46	-	-	21.3
							6												8							
							12												7							
VS24	male	28	37.3	3	B	tibia	26											delayed	9	<8						
							38												16							
							52												16							
							0	468.64	675.08	393.76	333.92	467.85									579.5555776	1559.46	47458.57	13.5	5.2	35.7
							6												7							
							12												7							
VS25	male	25	25.8	3	D	tibia	26											delayed	9	<8						
							38												16							
							52												16							
							0																			
							6												5							
							12	317.56	396.6	265.66	233.26	303.27							13		5729.000184	5,428.9	7,315.2	98.9	6.43	38.65
VS26	male	48	25.3	3	C	tibia	26											early	9	>9						
							38												16							
							52												16							
							0	424.4625	431.2125	516.015	545.6925	479.345625				0.35	24.965				1278.888928	3886.28	9882.09	-	13.4	21.3
							6	1722.488	1763.708	1520.46	1554.03	1640.1715				0.98	195.69		5		453.6666936	11,705.7	16,499.7	66.9	-	35.87
							12	343.3275	333.585	314.1	313.1775	326.0475				0.61	131.12		12		298.2222338	13,843.0	33,345.8	79.2	3.66	30.04
VS27	male	21	25.8	3	D	tibia	26						6	1640.172	1160.826			early	9	>9						
							38												14							
							52												16							
							0	221.84	266.4	167.28	158.2	203.43									1187.444475	2245.15	7574.55	8.8	43.8	18.3
							6												5							
							12	753.5	822.04	667.8	668.2	727.885							12		108.2222213	3,844.0	9,169.7	8.7		
VS28	male	24	24.4	3	A	femur	26											early	15	>9						
							38												16							
							52												16							

							0	576.28	674.44	503.48	494.42	562.155									638.7777593	9843.83	14607.09	-	-	-
							6												6							
							12	1098.9	1285.94	796.86	726.96	977.165							8		395.3333496	6,372.4	23,070.2	43.8	-	-
VS29	male	23	25.9	1	B	femur	26											delayed	11	<8						
							38												16							
							52												16							
							0	1266.233	1350.855	1588.433	1745.55	1487.76775				2.88	100.17				1389.444417	914.31	18725.74	100.9	-	28.7
							6	3454.425	2954.34	2743.425	3253.433	3101.40575				2.7	121.75		9		565.9999913	815.9	19,428.9	296.4	-	56.38
							12	2242.598	2306.048	1986.705	2159.505	2173.714				1.18	240.92		13							
VS30	male	18	18.7	3	D	femur	26						6	3101.406	1613.638			early	15	>9						
							38												16							
							52												16							
							0	795.22	815.82	535.42	551.38	674.46									1599.888881	1224.87	3828.37	26.8	-	-
							6	1552.92	1459.5	1800.94	1752.8	1641.54							12		809.2222412	3,523.6	28,890.3	43.3	-	19.80
							12												7							
VS31	male	25	26.8	3	C	femur	26											delayed	9	<8						
							38												16							
							52												16							
							0	216.8775	227.1375	317.97	317.1825	269.791875				0.345	29.4245				464.6666645	12944.20	5317.60	-	-	3.6
							6	995.5575	955.1925	812.6325	867.96	907.835625				0.7	92.959		8		201.3333439	27,481.3	13,911.9	-	-	16.41
							12	789.1875	792.5625	658.1925	689.0175	732.24				0.57	131.74		12		215.666655	27,145.0	18,184.1	-	-	11.68
VS32	female	18	27.2	3	B	femur	26						6	907.8356	638.0438			early	14	>9						
							38												16							
							52												16							
							0	1001.24	805.28	840.2	868.62	878.835									1443.111115	16858.47	12610.84	49.0	-	-
							6												5							
							12												7							
VS33	male	25	20.9	3	B	femur	26											delayed	9	<8						
							38												16							
							52												16							
							0	215.3475	154.44	188.955	216.09	193.708125				0.275	23.7745				1132.000036	39433.83	1170.80	21.2	6.3	-
							6	286.4025	301.725	371.3625	295.965	313.86375				0.7	93.741		7		459.6666627	17,030.6	7,768.3	39.5	-	-
							12	306.4275	281.7	233.9775	232.74	263.71125				0.32	94.103		9		1412.333329	53,442.5	17,391.0	114.9	-	-
VS34	female	26	27.5	1	C	femur	26						6	313.8638	120.1556			delayed	10	>9						
							38												16							
							52												13							
							0	315.7875	286.56	316.395	444.555	340.824375				0.525	73.872				1412.333329	2374.07	9932.22	-	-	-
							6	1460.003	1496.498	1029.578	1301.13	1321.80225				1.39	250.14		9		592.4444529	1,473.9	14,763.7	95.1	-	-
							12	1296.855	1409.243	1138.905	1178.573	1255.894				1.39	303.28		12		338.454553					
VS35	male	24	24.2	3	D	femur	26						6	1321.802	980.9779			early	9	>9						
							38												16							
							52												16							
							0	773.14	706.86	615.24	665.92	690.29									803.2727055	10544.16	-	-	5.7	-
							6	626.54	649.62	627.8	640.58	636.135							8		481.545473	11,952.3	6,796.3	84.8	-	43.84
							12												7							
VS36	female	35	22.1	3	C	femur	26											delayed	9	<8						
							38												16							
							52												16							
							0	291.96	280.935	357.8625	406.89	334.411875				0.4	46.3175				396.0000199	16765.76	3760.04	43.5	35.0	-
							6	280.53	244.665	233.055	287.5275	261.444375				0.5	108.85		7		362.1818074	8,031.0	3,173.2			
							12	653.04	625.1625	569.5875	524.205	592.99875				0.18	88.54		10		252.7272786	14,182.9	3,796.6	95.6	5.51	-
VS37	female	35	26.6	3	A	femur	26						12	592.9988	258.5869			normal	14	>9						
							38												16							
							52												16							
							0	123.4575	130.6575	266.6925		173.6025				0.37	64.918				1153.909112	18147.38	7529.00	75.7	15.6	-
							6	313.515	307.9125	252.4725	250.2675	281.041875				0.61	225.66		6		379.9090958	20,779.6	11,857.0	116.9	-	12.84
							12	498.195	465.0525	453.78	428.0625	461.2725				0.53	116.49		8		261.0000104	24,480.4	13,737.8	292.9	-	-

VS38	male	21	41.9	3	B	femur	26<br>38<br>52						12	461.2725	287.67			normal	12<br>16<br>16	<8						
VS39	male	36	34	3	D	femur	0<br>6<br>12<br>26<br>38<br>52	282.86<br>1309.56	249.1<br>1401.22	272.58<br>1204.32	338.2<br>1220.9	285.685<br>1284						normal	5<br>11<br>14<br>16<br>16	>9	1750.636461<br>454.545463	20560.27<br>11,280.2	2309.87<br>6,049.9	242.7<br>205.9	56.7<br>2.75	-<br>-
VS40	male	29	25.5	3	A	tibia	0<br>6<br>12<br>26<br>38<br>52	498.64<br>1178.4	456.26<br>1137.14	506.02<br>1146.42	489.58<br>1106.1	487.625<br>1142.015						delayed	8<br>7<br>9<br>16<br>16	<8	1019.363601<br>353.3636525	6314.61<br>4,864.6	4964.63<br>3,252.3	73.4<br>38.8	10.2<br>-	-<br>11.05
VS41	male	45	32.5	1	A	tibia	0<br>6<br>12<br>26<br>38<br>52	384.52<br>685.9	417.28<br>731.52	469.7<br>652.48	494.78<br>608.1	441.57<br>669.5						delayed	7<br>7<br>9<br>16<br>16	<8	974.1818177<br>375.000003	12668.83<br>7,640.7	1146.30<br>12,118.3	102.4<br>150.7	81.2<br>-	-<br>-
VS42	female	22	42.9	3	B	tibia	0<br>6<br>12<br>26<br>38<br>52	787.68<br>455.6<br>1305.5	829.64<br>528.12<br>1465.44	811.16<br>409.98<br>1369.66	632.78<br>406.94<br>1246.5	765.315<br>450.16<br>1346.775	12	1346.775	581.46			delayed	7<br>11<br>9<br>16<br>16	>9	1181.272683<br>288.9090997	18372.80<br>20,234.3	151.65<br>1,401.9	22.4<br>22.0	15.5<br>-	-<br>-
VS43	male	44	23.1	1	D	tibia	0<br>6<br>12<br>26<br>38<br>52	194.28<br>1143.6	260.44<br>1364.6	228.48<br>1069.24	229.8<br>1143.06	228.25<br>1180.125						early	7<br>12<br>9<br>16<br>16	>9	3247.999964<br>1503.090894	10487.90<br>12,244.9	2089.63<br>6,400.2	208.2<br>129.8	31.1<br>-	-<br>25.57
VS44	male	21	23.6	1	D	femur	0<br>6<br>12<br>26<br>38<br>52	649.4<br>2631.76	672.94<br>2287.34	579.32<br>2446.98	590.14<br>2564.12	622.95<br>2482.55						delayed	8<br>10<br>11<br>16<br>12	>9	215.7272754<br>69.09090856	2204.24<br>2,587.4	5885.44<br>12,060.4	39.9<br>37.3	88.5<br>90.59	179.4<br>269.71
VS45	male	33	34.8	3	A	femur	0<br>6<br>12<br>26<br>38<br>52	453.46<br>3217.38	469.6<br>3203.54	457.8<br>2800.98	461.48<br>2760.96	460.585<br>2995.715						early	8<br>12<br>9<br>16<br>16	>9	615.0000017<br>300.2727252	39889.78<br>16,602.8	3872.39<br>5,885.2	135.5<br>64.9	18.9<br>-	-<br>-
VS46	male	31	29.9	2	A	femur	0<br>6<br>12<br>26<br>38<br>52	611.02<br>1973.56	563.84<br>2175.38	564.82<br>1620.78	555.1<br>1681.6	573.695<br>1862.83						delayed	8<br>7<br>9<br>16<br>16	<8	1167.090896<br>565.6363561	4231.01<br>1,892.7	4780.37<br>8,798.9	66.8<br>34.4	39.4<br>-	-<br>-
VS47	female	29	22.4	1	B	tibia	0<br>6<br>12<br>26<br>38	509.4	300	349.92	491.1	412.605						delayed	5<br>7<br>9<br>16	<8	811.1818084	4055.66	7218.88	22.7	7.5	-

							52												16							
							0	300.44	249.98	281.76	260.98	273.29									636.5454552	1470.95	1043.35	97.4	8.6	-
							6												5							
							12												7							
VS48	male	44	25.5	3	C	femur	26											delayed	9	<8						
							38												16							
							52												16							
							0	649.18	586.88	614.8	567.2	604.515									909.7272513	30810.88	1100.15	96.3	13.2	32.1
							6												5							
							12												7							
VS49	female	30	30.1	2	B	tibia	26											delayed	9	<8						
							38												16							
							52												16							
							0	681.78	715.94	813.78	786.96	749.615									1057.181827	16494.43	5112.86	6.7	4.3	16.4
							6	1355.98	1396.22	1276.9	1308.26	1334.34							12		999.0909238	3,369.4	14,187.1	17.2	-	15.49
							12												7							
VS50	female	34	24.2	3	C	tibia	26											delayed	9	<8						
							38												16							
							52												16							
							0	434.72	512.84	533.04	474.68	488.82									191.3043329	43546.77	14999.97	2.5	-	-
							6	822.04	870.8	925.56	995.88	903.57							12		17.30435475	15,565.3	27,725.7	11.8	-	77.47
							12												7							
VS51	female	21	21.9	1	D	tibia	26											delayed	9	<8						
							38												16							
							52												16							
							0	363.82	419.22	455.2	418	414.06														
							6	1984.92	2056.5	1861.26	2137.64	2010.08							6		146.9565206	2,763.0	8,276.3	50.1	-	-
							12												7							
VS52	male	35	24.5	2	A	tibia	26											delayed	11	<8						
							38												16							
							52												16							
							0	569.22	607.36	667.88	592.52	609.245									415.565228	16158.83	3804.92	27.0	81.4	-
							6	925.7	854.9	807.88	781.72	842.55							8		534.3636142	6,310.5	12,674.0	47.5	30.18	-
							12												7							
VS53	male	29	23.5	1	C	tibia	26											delayed	9	<8						
							38												16							
							52												16							
							0	155.9025	146.025	177.3	186.2325	166.365				0.27	55.045				364.5454567	101942.88	4303.33	1.0	54.2	-
							6	684.72	652.68	595.2375	580.3875	628.25625				0.742116	214.81		8		473.9999856	21,509.8	9,159.9	22.5	-	77.47
							12	791.4375	844.425	483.615	412.9425	633.105				0.74092	200.69		11		442.4545524	28,892.0	15,899.2	41.8	-	63.86
VS54	female	26	33.7	3	D	tibia	26						12	633.105	466.74			normal	16	>9						
							38												16							
							52												16							
							0	594.22	641.84	627.58	620.22	620.965									688.8181869	2557.90	10783.25	31.6	37.8	-
							6	824.86	875.64	955.28	962.98	904.69							6		152.6956575	5,555.2	20,207.7	5.1	-	93.32
							12												6							
VS55	male	49	30.5	3	B	tibia	26											delayed	9	<8						
							38												16							
							52												16							
							0	808.515	869.6925	985.5225	659.835	830.89125				0.14	35.972				1168.608729	689.35	6983.91	48.3	21.2	-
							6	959.13	975.5325	1073.498	1136.385	1036.13638				0.334759	94.526		6		437.3043637	280.8	14,536.5	29.0	-	-
							12	1734.998	1866.24	1869.098	1602.068	1768.101				0.369792	136.72		10		537.7272669	375.4	24,273.7	25.3	-	97.16
VS56	male	29	25.2	1	C	femur	26						12	1768.101	937.2098			delayed	9	>9						
							38												16							
							52												16							
							0	449.82	382.36	369.56	366.12	391.965									2880.272684	10555.60	7705.51	69.7	137.2	-

							6												5							
							12												7							
VS57	male	47	24.4	3	D	femur	26											delayed	9	<8						
							38												16							
							52												16							
							0	783.2475	742.725	645.165	551.745	680.720625				0.537167	52.473				896.8181315	537.08	12978.29	23.0	59.7	-
							6	876.3525	944.91	1024.628	1046.948	973.209625				0.764279	183.18		6		346.1739152	622.1	30,700.9	40.8	-	50.01
							12	1000.8	924.165	1453.883	1289.948	1167.199				0.60963	150.586		7		496.9565107	817.1	35,569.9	45.1	-	-
VS58	male	28	23.8	2	D	femur	26						12	1167.199	486.4784			delayed	11	<8						
							38												16							
							52												16							
							0	410.6475	401.94	490.995	464.4225	442.00125				0.353105	54.158				421.217381	36601.69	9041.97	57.0	-	37.7
							6	950.175	734.175	797.7825	898.9425	845.26875				0.451034	219.72		11		139.8260817	16,860.7	23,134.3	42.3	44.27	-
							12	271.7775	298.0125	322.5375	369.2025	315.3825				0.757691	187.52		14		104.7826193	4,936.0	28,233.2	19.9	-	69.16
VS59	male	21	31.6	3	A	femur	26						6	845.2688	403.2675			early	9	>9						
							38												16							
							52												16							
							0	564.0975	583.965	677.0925	679.005	626.04				0.535443	54.929				420.173911	2901.11	23875.90	36.5	-	-
							6	993.6675	1036.508	562.3425	520.3125	778.207625				1.142701	170.77		5		250.0869519	2,619.4	35,183.9	19.7	-	-
							12	904.5675	914.1975	469.305	501.1425	697.303125				0.599573	228.23		12		180.2608621	1,838.1	34,288.7	30.8	-	-
VS60	male	22	19.1	2	C	tibia	26						6	778.2076	152.1676			early	15	>9						
							38												15							
							52												16							
							0	237.96	230.895	276.4125	306.72	262.996875				0.530838	58.42				1216.190478	10108.38	10102.93	7.6	215.9	193.0
							6	622.0125	621.675	487.755	367.9425	524.84625				1.493698	320.02		8		966.7618997	9,059.6	25,975.2	12.7	184.88	243.30
							12	450.4725	476.7525	309.7575	354.78	397.940625				0.454514	186.262		12		169.0434752	10,896.7	32,948.0	28.1	172.37	354.64
VS61	female	22	20.6	2	B	femur	26						6	524.8463	261.8494			early	16	>9						
							38												16							
							52												16							
							0	1617.098	1606.883	1745.19		1656.39033				0.376349	24.417				706.0000291	74083.52	6380.05	132.8	85.9	60.0
							6	1143.135	1147.073	1003.32	1072.553	1091.52025				0.563017	190.14		8		123.739138	42,507.0	17,950.6	57.5	0.45	81.75
							12	602.7525	512.3025	635.4225	665.7075	604.04625				0.467843	186.98		12		795.2631405	56,027.8	25,740.9	76.1	-	-
VS62	male	42	31.1	3	C	femur	26						6	1091.52	-564.8701			early	9	>9						
							38												16							
							52												16							
							0	397.215	377.325	415.53	456.7725	411.710625				0.735529	123.35				567.5652095	5580.38	10724.21	13.1	-	-
							6	541.305	582.4125	774.225	722.0475	654.9975				0.821192	173.494		5		247.217377	14,487.8	29,976.2	50.9	-	-
							12	813.4875	905.6475	1129.905	1155.443	1001.12075				0.665	179.828		7		505.1578917	8,021.5	18,894.3	10.6	-	-
VS63	male	20	27.4	3	B	femur	26						12	1001.121	589.4101			delayed	9	<8						
							38												16							
							52												16							
							0	299.56	377.44	381.38	386.28	361.165									885.1304382	1506.90	16662.89	-	-	-
							6	1404.84	1300.06	1409.96	1439.8	1388.665							11		211.0434994	4,336.2	23,020.5	7.3	-	-
							12												7							
VS64	male	24	22.1	3	C	femur	26											delayed	9	<8						
							38												16							
							52												16							
							0	2168.1	1822.94	1872.68	1668.36	1883.02									1441.826107	2921.53	29303.09	6.4	30.6	220.3
							6												8							
							12	1994.72	2329.14	1912.2	1764.3	2000.09							11		2523.789494	185.4	49,367.1	133.9	16.47	152.08
VS65	male	23	26	3	D	femur	26											delayed	9	>9						
							38												16							
							52												16							
							0	611.2125	592.245	638.7975	676.2375	629.623125				0.267801	56.416				243.4782739	29379.77	13996.83	8.2	21.2	100.3
							6	738.81	627.7275	564.5925	666.36	649.3725				0.812659	206.28		11		0	26,718.3	23,521.8	70.4	29.85	176.43
							12	1022.58	827.7075	656.5725	709.56	804.105				0.23	219.2		15		160.105262	17,974.7	20,745.0	105.8	5.30	32.24
VS66	male	20	24.2	3	A	femur	26						12	804.105	174.4819			early	9	>9						

							38												16							
							52												16							
							0									0.252391	34.154				455.1304284	28236.84	10529.13	-	13.9	-
							6												8							
							12												12							
VS67	female	25	32.7	3	B	femur	26											early	14	>9						
							38												15							
							52												16							
							0	311.625	247.6125	263.0925	370.1475	298.119375				0.385762	51.66				711.0434632	18667.93	16664.05	16.0	28.1	25.5
							6	977.4675	1012.928	944.1	1050.12	996.153875				0.70737	156.28		8		249.9999809	20,830.8	31,380.0	115.5	45.48	243.56
							12	162.09	147.42	193.77	192.5325	173.953125				0.395	160.18		10			23,465.3	19,391.5			
VS68	male	31	28.1	3	C	femur	26						6	996.1539	698.0345			delayed	10	>9						
							38												16							
							52												16							
							0	1199.14	1324.1	1702.7	1222.14	1362.02									736.0869507	34835.88	2030.64	61.8	17.3	-
							6												4							
							12												6							
VS69	female	42	27.8	3	D	femur	26											delayed	7	<8						
							38												9							
							52												16							
							0	470.88	363.96	273.105	277.11	346.26375				0.321789	58.877				441.6521856	26093.22	1648.81	32.2	-	-
							6	487.17	506.565	708.255	735.885	609.46875				0.615	164.036		9		693.1579057	21,028.4	5,454.9	14.4	-	-
							12	589.905	643.77	593.955	563.31	597.735				0.72	160.19		11		545.9999982	37,165.1	20,515.3	109.6	-	14.21
VS70	male	27	27.8	3	A	femur	26						6	609.4688	263.205			normal	14	>9						
							38												16							
							52												16							
							0	3010.2	2708.2	2978.1	2664.2	2840.175				1.173928	78.742				1583.217343	1539.70	9571.09	27.4	-	-
							6			959.2	1017.6	988.4				1.947953	221.99		5		737.2631468	3,885.9	29,465.6	19.5	-	10.71
							12			4777.9	4628.9	4703.4				0.8	179.08		9		299.4736816	7,134.4	35,108.2	71.6	-	29.20
VS71	male	23	25	3	C	tibia	26						12	4703.4	1863.225			normal	14	>9						
							38												16							
							52												16							
							0	258.4575	255.7575	221.3325	248.715	246.065625				0.430116	89.674				614.0869458	16201.43	4713.41	75.9	53.5	-
							6			506.97		506.97				0.47	160.32		8		478.4210362	21,571.7	20,898.9	18.6	33.01	37.34
							12			530.865	520.245	525.555				0.28	142.758		11		507.8947488	20,992.5	31,651.7	35.2	55.04	55.98
VS72	male	20	26.5	3	B	femur	26						12	525.555	279.4894			normal	15	>9						
							38												16							
							52												16							
							0																			
							6	1140.86	1511.4	1247.46	1155.76	1263.87							8		695.6842328	12,535.7	13,612.8	59.4	49.65	303.54
							12	1492.88	1788.14	1600.7	1510.94	1598.165							10		460.4210295	9,841.5	10,731.4	40.2	55.92	289.27
VS73	male	34	22.5	1	B	femur	26											delayed	9	>9						
							38												16							
							52												16							
							0	378.7875	394.3575	408.8475	405.9225	396.97875				0.320019	34.169				1447.913037	68872.42	1532.36	3.2	140.6	118.0
							6	1458.855	1440.518	1624.365	1617.39	1535.282				0.53	223.12		11		299.4736816	24,310.4	9,519.9	57.9	68.37	121.23
							12	424.215	332.1	566.595	566.3025	472.303125				0.24	143.95		16		503.7894632	48,805.3	11,754.6	39.2	80.17	104.20
VS74	male	28	25.8	3	B	femur	26						6	1535.282	1138.303			early	9	>9						
							38												16							
							52												16							
							0	233.28	163.08	330.32	256.1	245.695														
							6												5							
							12												7							
VS75	male	30	24.5	3	A	tibia	26											delayed	9	<8						
							38												16							
							52												16							

							0	198.3375	207.0225	173.8125	184.6575	190.9575				0.34	40.543				1086.00001	2002.59	8536.73	31.6	13.3	-
							6			295.9425	333.315	314.62875				0.48	136.32		5		570.5263056	5,353.7	15,441.0	122.3	-	3.56
							12	149.9175	353.5425	887.5575	818.5725	552.3975				0.56	179.72		5		0	3,541.0	17,813.9	58.8	-	7.17
VS76	male	28	27.1	2	A	tibia	26						12	552.3975	361.44			normal	12	<8						
							38												13							
							52												16							
							0	303.1	303.88	287.58	296.76	297.83									1376.421038	22551.56	8007.41	21.7	8.3	-
							6												7							
							12												7							
VS77	male	41	22.1	3	D	femur	26											normal	13	<8						
							38												15							
							52												16							
							0	692.32	722.76	722.98	688.86	706.73									3608.736829	21599.29	1057.43	47.3	-	-
							6	2942.1	2678.8	3025.72	2855.34	2875.49							12		1029.652162	21,606.9	11,715.4		-	8.42
							12												7							
VS78	male	48	39.2	3	A	femur	26											delayed	9	<8						
							38												16							
							52												16							
							0	773.6625	699.3675	653.2425	665.28	697.888125				0.265	92.676				1273.789432	30.99	9066.12	44.6	9.4	7.2
							6			1643.355	1483.133	1563.244				1.175	476.62		5		1049.36847	412.5	16,090.6	138.1	-	11.91
							12			2797.2	2858.895	2828.0475				0.605	401.52		12		993.052684	58.5	8,265.4	104.9	-	-
VS79	male	32	24.8	3	C	femur	26						12	2828.048	2130.159			early	15	>9						
							38												16							
							52												16							
							0	510.2	513.04	374.98	392.88	447.775									782.7368479	199.16	10806.22	204.3	33.1	-
							6												5							
							12												7							
VS80	male	26	23.4	2	C	femur	26											delayed	9	<8						
							38												16							
							52												16							
							0	396.3375	413.6625	408.06	468.99	421.7625				0.335	44.813				1808.421073	35060.82	9368.68	503.8	73.9	-
							6	264.7575	272.205	433.8	388.9125	339.91875				0.445	114.71		5		431.8947485	24,495.9	14,526.8	51.1	-	-
							12	356.805		356.6925	356.4	356.6325				0.515	96.559		10		552.8421095	17,265.9	16,396.2	153.4	2.69	11.91
VS81	male	31	37.7	2	B	tibia	26						12	356.6325	-65.13			normal	12	>9						
							38												16							
							52												16							
							0	353.24	302.1	369.32	259.22	320.97									1264.842147	22.11	13119.26	34.0	18.4	-
							6	652.52	658.1	627.22	647.9	646.435							7		549.3684449	342.6	25,661.5	170.7	-	9.61
							12												7							
VS82	male	22	21.4	3	C	femur	26											delayed	9	<8						
							38												16							
							52												16							
							0	315.24	252.98	319.52	271.66	289.85									3564.947367	506.01	2445.76	39.9	1.7	-
							6	258.44	277.84	178.16	251.42	241.465							7		551.3684317	1,254.2	12,892.2	79.4	-	3.56
							12												7							
VS83	male	30	19.2	3	C	tibia	26											normal	12	<8						
							38												16							
							52												16							
							0	267.615	295.0425	236.115	235.035	258.451875				0.41	25.157				373.2631652	2451.38	3140.87	35.7	33.1	-
							6	215.325	230.3325	447.66	473.625	341.735625				1.145	132.036		7		704.9473593	5,000.9	22,180.2	51.4	-	28.11
							12		623.34	593.46	543.555	586.785				0.65	141.48		10		437.9999895	5,731.8	17,016.6	75.4	5.28	21.83
VS84	male	25	25.9	3	B	femur	26						12	586.785	328.3331			normal	12	>9						
							38												14							
							52												16							
							0	363.54	302.56	254.62	264.86	296.395									1096.947407	92292.93	-	166.8	18.9	-
							6												8							
							12	1595.58	1508.56	1412.28	1575.52	1522.985							12		519.7894517	50,462.1	802.5	143.0	-	-

VS85	female	46	42.8	3	D	femur	26											early	13	>9						
							38												16							
							52												16							
							0	271.7	296.02	194.72	212.82	243.815									1139.789532	13813.21	2312.58	63.2	26.3	-
							6	546.9	408.82	380.72	347.42	420.965							6		354.9473637	33,234.6	12,241.0	97.2	-	33.30
							12												7							
VS86	female	37	26.5	3	A	tibia	26											normal	16	<8						
							38												16							
							52												16							
							0	364.46	319.72	393.3	684.8	440.57									712.857154	33386.30	20700.03	22.5	1.3	-
							6	1253.68	1303.46	*5966.9	1837.98	1465.04							8		416.2857006	33,870.8	31,652.8	85.3	-	-
							12												12							
VS87	male	23	24.6	3	A	femur	26											early	9	>9						
							38												16							
							52												16							
							0	267.12	277.4925	242.28	272.835	264.931875				0.195	55.513				1003.047618	31673.27	1752.58	103.9	-	-
							6	349.7175	302.985	424.6425	421.9875	374.833125				0.4	200.36		8		733.3333363	27,801.1	18,170.6	163.1	-	-
							12	205.11	248.0175	338.04	369.6975	290.21625				0.4	185.13		12		612.6666564	31,828.8	15,669.0	175.7	-	-
VS88	female	43	28.7	3	B	tibia	26						6	374.8331	109.9013			early	16	>9						
							38												16							
							52												16							
							0	267.12	307.62	221.0625	219.9825	253.94625				0.31	44.842				363.6842125	41906.36	2163.92	6.8	15.3	-
							6	277.02	300.0375	536.7825	483.075	399.22875				0.47	182.16		7		340.0000089	13,853.9	10,721.2	172.4	-	24.62
							12	367.425	392.85	580.77	567.18	477.05625				0.51	157.51		12		278.5263193	14,567.1	15,021.1	82.7	-	37.43
VS89	female	23	18.4	3	C	femur	26						12	477.0563	223.11			early	9	>9						
							38												16							
							52												16							
							0	448.065	452.5425	440.5275	463.95	451.27125				0.5	45.675				939.7142769	3090.14	15711.16	-	-	-
							6	875.8125	812.925	928.9125	1023.075	910.18125				0.94	423.9		9		245.8095194	723.9	33,631.1	18.5	-	-
							12	1096.178	961.245		1442.565	1166.66267				0.56	141.36		12		305.3333289	1,022.4	38,692.8	8.6	-	5.57
VS90	male	21	23.4	3	A	femur	26						12	1166.663	715.3914			early	9	>9						
							38												16							
							52												16							
							0	166.635	113.22		618.885	299.58				0.34	23.185				776.9523843	13252.45	7629.27	4.9	55.8	-
							6	412.425	412.6725	435.735	429.345	422.544375				0.43	129.29		5		665.9047588	9,184.5	21,962.2	62.4	11.31	16.63
							12	465.5025	428.085	522.0225	564.2325	494.960625				0.38	111.77		10		894.571422	11,251.4	23,011.9	48.3	-	-
VS91	female	26	20.3	3	D	femur	26						12	494.9606	195.3806			delayed	10	>9						
							38												16							
							52												16							
							0	345.42	374.8275	332.9325	355.32	352.125				0.41	41.924				1184.476209	27318.42	7674.24	66.5	36.6	-
							6	1048.365	866.565		1186.043	1033.65767				0.62	160.91		9		895.1428318	51,722.3	21,285.5	275.8	13.68	-
							12	700.5825	601.335	742.1175	765.585	702.405				0.33	163.08		12		618.4762076	24,209.0	23,809.6	227.2	-	-
VS92	female	34	32.1	3	D	tibia	26						6	1033.658	681.5327			early	16	>9						
							38												16							
							52												16							
							0			77.9175	65.3175	71.6175				0.37	21.663				1329.523772	24091.10	2968.60	64.6	42.9	-
							6	721.485	820.44	740.835	792.6975	768.864375				1.1	211.79		8		537.8095236	15,021.1	2,369.4	112.4	-	-
							12	529.3575	460.26	440.9325	338.1975	442.186875				0.62	203.52		11		291.714292	35,758.4	6,086.9	135.1	48.09	-
VS93	male	32	30.3	3	B	femur	26						6	768.8644	697.2469			delayed	9	>9						
							38												16							
							52												16							
							0	190.4175	217.9125	167.85	174.8925	187.768125				0.34	40.716				829.9047534	74525.78	13918.66	30.2	-	-
							6	833.535	850.2075	607.185	690.5925	745.38				0.4	87.009		6		376.9523968	33,241.1	21,878.2	114.5	-	56.06
							12	738.315	629.1	436.23	475.7625	569.851875				0.38	65.831		5		1112.476183	42,074.2	17,832.7	91.8	-	94.71
VS94	female	27	33.3	2	A	femur	26						6	745.38	557.6119			delayed	7	<8						
							38												15							

							52												16							
							0	590.7375	610.965	579.285	600.615	595.400625				0.44	53.954				835.6190223	70759.66	9451.21	87.8	52.9	-
							6	680.625	753.7725	670.0275	975.2175	769.910625				0.58	102.54		8		243.1428502	66,763.1	17,959.5	196.3	-	-
							12	1033.853	1032.908	940.05	884.0925	972.725875				0.75	152.956		11		241.2380844	72,928.2	22,382.4	194.9	27.67	-
VS95	female	18	39.3	3	B	femur	26						12	972.7259	377.3253			normal	15	>9						
							38												16							
							52												16							
							0	1071.203	1197	1205.82	1296.36	1192.59575				0.11	37.395				1835.428639	10064.57	9053.28	183.1	-	-
							6	2292.998	2405.115	2893.658	3066.75	2664.63025				0.47	221.7		6		1358.380992	5,955.6	8,255.3	335.3	-	27.92
							12	1913.828	1805.175	1529.393	1659.263	1726.91475				0.17	141.31		8		458.7619005	7,407.9	6,545.7	204.4	-	38.36
VS96	male	48	20	3	C	femur	26						6	2664.63	1472.035			delayed	11	<8						
							38												16							
							52												16							
							0	212.8725	190.1025	276.57	342.405	255.4875				0.25	20.403				2243.904776	4884.72	1327.46	168.1	54.5	-
							6	778.6575	799.83	842.9625	615.195	759.16125				0.4	164.662		10		690.666657	6,757.7	10,372.7	98.4	-	-
							12	790.83	855.5625	954.4275	916.3125	879.283125				0.39	246.78		12		889.142858	11,187.8	13,702.6	138.3	-	17.92
VS97	male	46	29.1	3	D	femur	26						12	879.2831	623.7956			early	13	>9						
							38												16							
							52												16							
							0	168.345	147.645	246.285	279.2025	210.369375				0.39	56.97				1412.095278	81707.26	15626.51	192.0	58.7	-
							6	225.54	271.4175	291.69		262.8825				0.32	210.9		7		630.952368	45,677.3	26,451.1	39.9	30.83	-
							12	203.0175	250.155	375.9075	271.8675	275.236875				0.69	103.22		9		180.347833	56,044.3	5,981.6		44.06	-
VS98	female	26	34.3	3	A	femur	26						12	275.2369	64.8675			normal	15	>9						
							38												16							
							52												16							
							0	2186.5	2156.9	3515.52	3490.08	2837.25									1617.23806	3501.05	6177.69	89.0	47.5	-
							6	2963.54	2887.9	3081.16	3228.74	3040.335							8		267.2380988	2,959.8	2,807.0	108.2	-	-
							12												7							
VS99	male	41	23.8	3	C	tibia	26											delayed	9	<8						
							38												16							
							52												16							
							0	323.56	326.32	291.66	348.04	322.395									1817.714196	22208.73	1538.33	40.5	-	-
							6	1726.84	1876.84	2091.5	2075.88	1942.765							8		523.0476103	2,959.8	2,807.0		-	-
							12												7							
VS100	female	21	24.4	3	B	femur	26											delayed	9	<8						
							38												16							
							52												16							
							0	444.14	464.44	529.28	500.6	484.615									382.9523707	5152.92	2927.21		10.2	-
							6	1044.66	1086.32	1241.62	1100.8	1118.35							8		282.9523809	9,517.6	6,690.9		6.45	-
							12												7							
VS101	female	21	28.3	3	C	femur	26											normal	16	<8						
							38												16							
							52												16							
							0	293.68	263.5	343.8	301.02	300.5									1390.190458	801.81	1315.33		15.2	-
							6												5							
							12												8							
VS102	female	30	21.2	1	D	tibia	26											delayed	9	<8						
							38												11							
							52												16							
