# Supplementary material for: Efficacy of biomarkers in the endochondral phase of fracture repair and healing in long bones: A clinical observational studys
Source: PLoS Med. 2025 Aug 29;22(8):e1004640. doi: 10.1371/journal.pmed.1004640 (PMC12410876; doi:10.1371/journal.pmed.1004640)
Supplement: S1 Code — SAS code for the analysis of data from the VitaShock phase II exploratory randomized clinical trial for Figs 2–4 and S1–S3. (RTF) [file pmed.1004640.s010.rtf]

 ****************************************************** /* CXM Biomarker has Strong Association with Canonical  Bone Turn Over Markers During Fracture Repair */ ****************************************************** ****************************************************** ******************************************************  *********** Paragraph 1 *********** *association between CXM and age; proc corr data = cxm_final pearson spearman; var age CXM log_cxm; where weeks = 0; run;  *check the residuals; proc reg data = cxm_final; model age = log_cxm; where weeks = 0; run;  proc sgplot data = cxm_final; scatter x = age y = log_cxm; /* scatter x = age y = cxm; */ where weeks = 0; run;  proc ttest data = cxm_final; var cxm log_cxm; class sex; where weeks = 0; run;  proc npar1way data = cxm_final wilcoxon; var cxm log_cxm; class sex; where weeks = 0; run;  *********** Paragraph 2 *********** *this analysis was verified by replicating the shock paper; proc mixed data = cxm_final; *figure 2B; class ID  vd3_group (ref = 'C'); *ref C = placebo; model log_CXM = vd3_group time_spline time /s; random intercept/ subject = id; lsmeans vd3_group/ e cl diff; run;  *********** Paragraph 3 ***********;  proc univariate data = cxm_final; *check distributions; var log_CXM log_PINP cxm pinp gdf log_gdf; histogram ; class weeks; run;  proc mixed data = cxm_final; *does P1NP change with CXM accounting for non linear change in time; class  id t ; model log_CXM = log_PINP time_spline time/ solution cl; random intercept/ subject = id; run;  proc mixed data = cxm_final; *Does CTX chage with CXM?; class  id t ; model log_CXM = log_CTX time_spline time/ solution cl; random intercept/ subject = id; run;  proc sort data = cxm_final;  by weeks; run;  proc corr data = cxm_final;  var log_cxm cxm log_pinp pinp log_ctx ctx  ; run;  proc corr data = cxm_final; *correlations by week; var log_cxm cxm log_pinp pinp log_ctx ctx  ; by weeks;*Fig 2E; run;  proc sgpanel data = cxm_final; *visualization on log scale; panelby weeks; *Fig 2 C and D; scatter x = log_ctx y = log_cxm; reg x = Log_ctx y = log_cxm; /* yaxis TYPE=LOG LOGSTYLE=LOGEXPAND LOGBASE=10; */ /* xaxis TYPE=LOG LOGSTYLE=LOGEXPAND LOGBASE=10; */ run;  proc sgplot data = cxm_final; *visualization of correlation at baseline; /* scatter x = log_ctx y = log_cxm; */ reg x = log_ctx y = log_cxm; where weeks = 0; run;  proc sgpanel data = cxm_final; *visualization on log scale; panelby weeks; *Fig 2 C and D; scatter x = log_PINP y = log_cxm; reg x = log_PINP y = log_cxm; /* yaxis TYPE=LOG LOGSTYLE=LOGEXPAND LOGBASE=10; */ /* xaxis TYPE=LOG LOGSTYLE=LOGEXPAND LOGBASE=10; */ run;  proc corr data = cxm_final pearson spearman plots = matrix; *correlations with other markers; var  Log_GDF log_SOST log_leptin log_OC    ; with log_CXM ; *Fig 2F; by weeks; /* where log_GDF>3; */ run;   proc sgpanel data = cxm_final; *visualization of leptin and cxm; panelby weeks; scatter x = log_leptin y = log_cxm; reg x = log_leptin y = log_cxm; /* yaxis TYPE=LOG LOGSTYLE=LOGEXPAND LOGBASE=10; */ /* xaxis TYPE=LOG LOGSTYLE=LOGEXPAND LOGBASE=10; */ run;  proc sort data = cxm_final; by weeks; run;  proc corr data = cxm_final plots = matrix (nvar=all); var  Log_GDF log_SOST log_leptin log_OC gdf SOST leptin osteocalcin; with log_CXM ; by weeks; run;   *********** /* ICXM Biomarker Demonstrates Significant Correlations  with Protein Expressions Related to Fracture Healing  and Distinguishes Patients with Accelerated Healing*/ ***********;  proc freq data = cxm_final; table mRust_healer; *normal, early, delayed; where weeks = 0; run;  proc freq data = cxm_final; *healer status differ by sex at baseine?; tables sex*mRUST_healer/fisher; where weeks = 0; run;  proc means data = cxm_final; *descriptive numbers for CXM by week and healer status; var log_cxm cxm; class weeks mRUST_healer; run;  proc mixed data = cxm_final; *is mRUST status associated with CXM, controlling for weeeks; class  id weeks mrust_healer; *Fig 3B; model log_CXM =  mrust_healer  weeks / solution cl; lsmeans mrust_healer/e cl diff; random int/subject =  id; run;  proc mixed data = cxm_final covtest; *model for determining if mRUST category is associate with cxm values across time; class mrust_heal id weeks; model log_cxm = mRust_heal|weeks/s; lsmeans mRust_heal*weeks/pdiff; lsmeans mRust_heal/pdiff; lsmeans weeks/pdiff; repeated / subject = id type = un; run;   proc mixed data = cxm_final; *describe non-linear relationship with time; class ID mRUST_healer ; model log_CXM = mRUST_healer time_spline time /s; /* random intercept/ subject = id; */ repeated / subject = id ; /* lsmeans mRUST_healer/ e cl diff; */ run;   /*Did not use this model, convergence issues determining if CXM could predict mRUST healer category */  proc glimmix data = cxm_final method = laplace  noclprint;  class id  mRUST_healer ; model mRUST_healer (event = 'early') =  log_cxm|weeks/s  dist= multinomial link = glogit  ddfm = bw; random int weeks/ subject = id type = un group = mrust_healer; NLOPTIONS MAXITER= 100000 maxfunc=10000; parms/ noiter lowerb=1e-4,.,1e-4; estimate 'log cxm' log_cxm 1/ cl exp; run;   proc sort data = cxm_final; by mRUST_healer; run;  proc corr data = cxm_final plots = matrix (nvar=all);*correlations by mRUST cat; var  Log_GDF log_SOST log_leptin log_OC ; with log_CXM ; *Figure 3CDE; by mRUST_healer; run;   proc corr data = cxm_final plots = matrix (nvar=all); *correlations at baseline only; var  Log_GDF log_SOST log_leptin log_OC ; with log_CXM ; by mRUST_healer; where weeks = 0; run;  proc corr data = cxm_final plots = matrix (nvar=all); *6 weeks; var  Log_GDF log_SOST log_leptin log_OC ; with log_CXM ; by mRUST_healer; where weeks = 6; run;  proc corr data = cxm_final plots = matrix (nvar=all); *12 weeks; var  Log_GDF log_SOST log_leptin log_OC ; with log_CXM ; by mRUST_healer; where weeks = 12; run;   proc mixed data = cxm_final plots; *does CXM differ between males and females, controlling for week?; class weeks sex id; model log_CXM = sex weeks/s; *figure 4a; lsmeans weeks sex/pdiff adjust = tukey; random intercept/ subject= id; run;  proc mixed data = cxm_final plots; *does CXM differ by fracture pattern, controlling for weeks?; class weeks tib_fem id; *figure 4b; model log_CXM = tib_fem|weeks/s; lsmeans weeks*tib_fem/pdiff adjust = tukey; random intercept/ subject= id; run;   
