# Supplementary material for: Efficacy of biomarkers in the endochondral phase of fracture repair and healing in long bones: A clinical observational studys
Source: PLoS Med. 2025 Aug 29;22(8):e1004640. doi: 10.1371/journal.pmed.1004640 (PMC12410876; doi:10.1371/journal.pmed.1004640)
Supplement: S2 Code — SAS code for the analysis of repeatability and reliability data comparing CXM data from serum and dried blood spots. Data was displayed in Figs 5 and S4. (RTF) [file pmed.1004640.s011.rtf]

  /*This code assesses the relationship between dried blood spots compared to gold standard serum samples  */    data dbs_serum_wide; set dbs_serum_wide; *compute new variables; 	percent_change_dbs = (DBS_ave_2 - DBS_ave)/DBS_ave; 	percent_change_serum = (Serum_ave_2 - Serum_ave)/Serum_ave; 	abs_change_dbs = DBS_ave_2 - DBS_ave; 	abs_change_serum = Serum_ave_2 - Serum_ave; 	diff_dbs_serum = DBS_ave-Serum_ave; 	mean_diff = (DBS_ave+Serum_ave)/2; run;  /*check to see if correct num of datapoints  */ proc freq data = dbs_serum; tables time plate rep type; run;  /*check and compare distributions */  proc means data = dbs_serum maxdec=2; var CXM; class time type plate rep ; run;  /*time 1 */ proc univariate data = dbs_serum_wide; var DBS_p1r1	DBS_p1r2	DBS_p2r1	DBS_p2r2	Serum_p1r1	Serum_p1r2	Serum_p2r1	Serum_p2r2; histogram DBS_p1r1	DBS_p1r2	DBS_p2r1	DBS_p2r2	Serum_p1r1	Serum_p1r2	Serum_p2r1	Serum_p2r2/normal; run;  /*time 2 */ proc univariate data = dbs_serum_wide; var DBS_p1r1_2	DBS_p1r2_2	DBS_p2r1_2	DBS_p2r2_2	Serum_p1r1_2	Serum_p1r2_2	Serum_p2r1_2	Serum_p2r2_2; histogram DBS_p1r1_2	DBS_p1r2_2	DBS_p2r1_2	DBS_p2r2_2	Serum_p1r1_2	Serum_p1r2_2	Serum_p2r1_2	Serum_p2r2_2/normal; run;   proc sgplot data = dbs_serum; histogram CXM/ group = time  transparency=0.5; where type = "DBS"; run;  proc sgplot data = dbs_serum; histogram CXM/ group = time  transparency=0.5; where type = "Serum"; run; /*identified an outlier in serum - do we believe this is a real datapoint?  */  proc sgpanel data=dbs_serum;   panelby time plate;   histogram CXM / group = rep transparency = .5 ;   density CXM/ type = normal group =  rep;   where type = "DBS"; run;  proc sgpanel data=dbs_serum;   panelby time plate;   histogram CXM / group = rep transparency = .5 ;   density CXM/ type = normal  group =  rep;   where type = "Serum" and CXM < 3000; run; ***************************;  ***************************;  ***************************;  ***************************;  ***************************;  ***************************; /*are values different across replicates?*/ /*No, values are not different across replicates at either time  */ /*dbs - time 1 plate 1*/ proc ttest data = dbs_serum_wide; paired DBS_p1r1*DBS_p1r2; run;  /*dbs - time 1 plate 2*/ proc ttest data = dbs_serum_wide; paired DBS_p2r1*DBS_p2r2; run;  /*serum - time 1 plate 1*/ proc ttest data = dbs_serum_wide; paired serum_p1r1*serum_p1r2; run;  /*serum - time 1 plate 2*/ proc ttest data = dbs_serum_wide; paired serum_p2r1*serum_p2r2; run;  /*are values different across replicates? time 2*/ /*dbs - time 2 plate 1*/ proc ttest data = dbs_serum_wide; paired DBS_p1r1_2*DBS_p1r2_2; run;  /*dbs - time 2 plate 2*/ proc ttest data = dbs_serum_wide; paired DBS_p2r1_2*DBS_p2r2_2; run;  /*serum - time 2 plate 1*/ proc ttest data = dbs_serum_wide; paired Serum_p1r1_2*Serum_p1r2_2; run;  /*serum - time 2 plate 2*/ proc ttest data = dbs_serum_wide; paired Serum_p2r1_2*Serum_p2r2_2; run;  ***************************;  ***************************;  ***************************;  ***************************;  ***************************;  /*are values different across plates  */ /*time 1  */ proc ttest data = dbs_serum_wide; paired DBS_p1r1*DBS_p2r1; run;  proc ttest data = dbs_serum_wide; paired DBS_p1r2*DBS_p2r2; run;  proc ttest data = dbs_serum_wide; paired Serum_p1r1*Serum_p2r1; run;  proc ttest data = dbs_serum_wide; paired Serum_p1r2*Serum_p2r2; run;  /*time 2  */ proc ttest data = dbs_serum_wide; paired DBS_p1r1_2*DBS_p2r1_2; run;  proc ttest data = dbs_serum_wide; paired DBS_p1r2_2*DBS_p2r2_2; run;  proc ttest data = dbs_serum_wide; paired Serum_p1r1_2*Serum_p2r1_2; run;  proc ttest data = dbs_serum_wide; paired Serum_p1r2_2*Serum_p2r2_2; where Serum_p2r2_2 < 3000; run;  /*does the sample change across time? */ /*dbs */ proc ttest data = dbs_serum_wide; paired DBS_p1r1*DBS_p1r1_2; run;  proc ttest data = dbs_serum_wide; paired DBS_p1r2*DBS_p1r2_2; run;  proc ttest data = dbs_serum_wide; paired DBS_p2r1*DBS_p2r1_2; run;  proc ttest data = dbs_serum_wide; paired DBS_p2r2*DBS_p2r2_2; run; /*serum */ proc ttest data = dbs_serum_wide; paired Serum_p1r1*Serum_p1r1_2; run;  proc ttest data = dbs_serum_wide; paired Serum_p1r2*Serum_p1r2_2; run;  proc ttest data = dbs_serum_wide; paired Serum_p2r1*Serum_p2r1_2; run;  proc ttest data = dbs_serum_wide; paired Serum_p2r2*Serum_p2r2_2; where Serum_p2r2_2 < 3000; run;  /* Looks like serum is slightly more stable as of now but let's see what happens when we  average within plates and compare t1 to t2. */  /*dbs  */ proc ttest data = dbs_serum_wide; title "change over time dbs"; paired P1*P1_2; run;  proc ttest data = dbs_serum_wide; paired P2*P2_2; run;  /*serum  */  proc ttest data = dbs_serum_wide; title "change over time serum"; paired P1_1*P1_2_1; run;  proc ttest data = dbs_serum_wide; paired P2_1*P2_2_1; run; title;  proc ttest data = dbs_serum_wide; paired dbs_ave*dbs_ave_2; run;  proc ttest data = dbs_serum_wide; paired serum_ave*serum_ave_2; where Serum_p2r2_2 < 3000; run;  proc ttest data = dbs_serum_wide; paired serum_ave*dbs_ave; /* where Serum_p2r2_2 < 3000; */ run;  proc means data = dbs_serum_wide mean std maxdec = 1; var  dbs_ave  dbs_ave_2 serum_ave  serum_ave_2; run;  proc ttest data = dbs_serum_wide; paired percent_change_dbs*percent_change_serum; run;  /*Yes, serum is more stable */ /*we can visualize with proc corr */ proc corr data = dbs_serum_wide; var p1 p2 p1_1 p2_1 p1_2 p2_2 p1_2_1 p2_2_1; run;   proc sgscatter data = dbs_serum_wide; plot ( p1_2 p2_2)*(p1  p2)  / reg = (nogroup clm); run;  proc sgplot data = dbs_serum; vbox CXM/ group = type; where time =1; run;  proc sgplot data = dbs_serum; vbox CXM/ group = type; where time =2; run;  /*now we need to use a regression to see if serum can predict dbs  */  proc reg data = dbs_serum_wide plots = (CooksD(label)); model serum_ave = dbs_ave/ r influence ; output out = regout pred = Pred; /* where id ~= "V.C135" and id ~= "V.C136"; */ run;  /*bland altman plot  */  proc sql  ; create table means as select mean(diff_dbs_serum)-2*std(diff_dbs_serum) as lower, mean(diff_dbs_serum)+2*std(diff_dbs_serum) as upper from dbs_serum_wide ; quit;   proc sgplot data = dbs_serum_wide ; scatter x = mean_diff y = diff_dbs_serum; refline 0 847 -123 /LABEL = ("zero bias line" "95% upper limit" "95% lower limit"); TITLE 'Bland-Altman Plot'; run ; quit ;  proc sort data = dbs_serum; by type id plate rep; run;  /*What is the avg difference between dbs and serum */ proc mixed data = dbs_serum; class type id rep plate; model CXM = type/ s outpred= r; random int/ subject = id; /* random int / subject= id (rep*plate); */ /* where time = 1; */ /* where time = 2; */ run; quit;  proc ttest data = dbs_serum_wide; paired dbs_ave*serum_ave; run;            
