# Supplementary material for: Efficacy of biomarkers in the endochondral phase of fracture repair and healing in long bones: A clinical observational studys
Source: PLoS Med. 2025 Aug 29;22(8):e1004640. doi: 10.1371/journal.pmed.1004640 (PMC12410876; doi:10.1371/journal.pmed.1004640)
Supplement: S3 Code — SAS code for the analysis of data from the prospective fracture data in Figs 6, 7, and S5. (RTF) [file pmed.1004640.s012.rtf]

*********** *********** /*Longitudinal Prospective Study  Identifies CXM Peaks During Early  Endochondral Phase of Repair  */ *********** ***********; *LP only did part of the analysis for this section;  data ohsu_long_final; set cxm.ohsu_long_final; run;  *basic descriptive, sex age and fracture type;  proc freq data = ohsu_long_final; table sex fracture sex*fracture/chisq; where visit = 1; run;  proc univariate data = ohsu_long_final; var age; histogram; where visit = 1; run;  proc means data = ohsu mean std median Q1 Q3 maxdec = 1; var age; class sex; where id ~=738; run;  proc means data = ohsu_long_final mean std median q1 q3 maxdec = 1; var age; class fracture; where visit = 1; run;  proc npar1way data = ohsu wilcoxon; var age; class fracture; where id ~= 738; run;  *CXM and subject characteristics;  proc univariate data = ohsu_long_final normaltest plots; var cxm log_cxm; histogram; /* where visit = 1; */ run;  proc means data = ohsu_long_final; var log_cxm cxm;  class sex; where visit = 1; run;   *Spaghetti plot for visualizing individual data; proc sgplot data = ohsu_long_final; series x = days y = cxm/ group = id; xaxis TYPE=LOG LOGSTYLE=LOGEXPAND LOGBASE=10; yaxis TYPE=LOG LOGSTYLE=LOGEXPAND LOGBASE=10; run;  proc sgplot data = ohsu_long_final; *A look at how CXM changes over time overall; loess x = days y = CXM/ interpolation=cubic group = sex; /* xaxis TYPE=LOG LOGSTYLE=LOGEXPAND LOGBASE=10; */ run; *figure 6A;  proc sort data = ohsu_long_final; by days; run;  ********* ********* /* Relationship Between Endochondral  Healing and Patient Demographics */ ********* *********;  /*where are the peak days - days where CXM is the highest?  */ proc means data = ohsu_long_final maxdec = 1 mean std median q1 q3; var peak_day peak_CXM; where peak_day ~=.; run;  proc sgplot data = ohsu_long_final; loess x = peak_day y = cxm;  xaxis TYPE=LOG LOGSTYLE=LOGEXPAND LOGBASE=10;  run;  *does peak day differ by sex or fracture type?; proc ttest data = ohsu_long_final; var peak_CXM peak_day; class sex; where peak_day ~=.; run;  proc means data = ohsu_long_final maxdec = 1 mean std median q1 q3; var peak_day peak_CXM; class sex; where peak_day ~=.; run;  proc ttest data = ohsu_long_final; var peak_CXM peak_day; class fracture; where peak_day ~=.; run;  proc means data = ohsu_long_final maxdec = 1 mean std median q1 q3; var peak_day peak_CXM; class fracture; where peak_day ~=.; run;  proc mixed data = ohsu_long_final; *not associated with age controlling for time; class sex id; model log_CXM = age days/s ; random int / subject= id; run;  proc mixed data = ohsu_long_final; *sex is related with Females having higher values but only just; *time as a random effect, but we are still modeling it as linear here. See glimmix models below for splines; class sex id; model log_CXM = sex days /s ; lsmeans sex/diff; random int / subject= id; /* random days; */ run;   *This code investigates sex and cxm using cubic splines; proc glimmix data = ohsu_long_final outdesign(x)=Xmatrix ; class sex id ; effect spl = spline(days/naturalcubic BASIS=TPF(NOINT) details knotmethod = percentiles(4)) ; model log_CXM = spl sex/s ; lsmeans sex/diff; random intercept / subject= id;    output out=SplineOut predicted=Fit;            /* output predicted values for graphing */ quit;     
