# Supplementary material for: Efficacy of biomarkers in the endochondral phase of fracture repair and healing in long bones: A clinical observational studys
Source: PLoS Med. 2025 Aug 29;22(8):e1004640. doi: 10.1371/journal.pmed.1004640 (PMC12410876; doi:10.1371/journal.pmed.1004640)
Supplement: S1 Table — Tables containing detailed statistical results from each figure. (DOCX) [file pmed.1004640.s013.docx]

**SUPPORTING INFORMATION – Tables of statistics for each figure.**

| **Figure 1 Statistics** | | | | | | | | | | | | | | | | | | | | | | | | | | | | | | | | | | | | | | | | | | | | | | | | | | | |  |  |  |  |  |  |  |  |  |  |  |  |  |  |  |  |  |  |  |  |  |  |  |  |  |  |  |  |  |  |  |  |  |
| --- | --- | --- | --- | --- | --- | --- | --- | --- | --- | --- | --- | --- | --- | --- | --- | --- | --- | --- | --- | --- | --- | --- | --- | --- | --- | --- | --- | --- | --- | --- | --- | --- | --- | --- | --- | --- | --- | --- | --- | --- | --- | --- | --- | --- | --- | --- | --- | --- | --- | --- | --- | --- | --- | --- | --- | --- | --- | --- | --- | --- | --- | --- | --- | --- | --- | --- | --- | --- | --- | --- | --- | --- | --- | --- | --- | --- | --- | --- | --- | --- | --- | --- | --- | --- |
|  | | **1A** | | | **regression line equation** | | | | | Y = 1.507x + 558.7 | | | | | | | | | | | | | | | | | | |  | | | | | | | | | | | | | | | | | | | | | | |  |  |  |  |  |  |  |  |  |  |  |  |  |  |  |  |  |  |  |  |  |  |  |  |  |  |  |  |  |  |  |  |  |
|  | |  | | | **slope 95% CI** | | | | | -1.146 to 4.160 | | | | | | | | | | | | | | | | | | |  | | | | | | | | | | | | | | | | | | | | | | |  |  |  |  |  |  |  |  |  |  |  |  |  |  |  |  |  |  |  |  |  |  |  |  |  |  |  |  |  |  |  |  |  |
|  | |  | | | **Spearman r** | | | | | 0.03776 | | | | | | | | | | | | | | | | | | |  | | | | | | | | | | | | | | | | | | | | | | |  |  |  |  |  |  |  |  |  |  |  |  |  |  |  |  |  |  |  |  |  |  |  |  |  |  |  |  |  |  |  |  |  |
|  | |  | | | **95% CI** | | | | | -0.1551 to 0.2279 | | | | | | | | | | | | | | | | | | |  | | | | | | | | | | | | | | | | | | | | | | |  |  |  |  |  |  |  |  |  |  |  |  |  |  |  |  |  |  |  |  |  |  |  |  |  |  |  |  |  |  |  |  |  |
|  | | **1B** | | |  | | | | | **Male** | | | | | | | | | | | | | | | | | | | **Female** | | | | | | | | | | | | | | | | | | | | | | |  |  |  |  |  |  |  |  |  |  |  |  |  |  |  |  |  |  |  |  |  |  |  |  |  |  |  |  |  |  |  |  |  |
|  | |  | | | **Median** | | | | | 538.4 | | | | | | | | | | | | | | | | | | | 589.1 | | | | | | | | | | | | | | | | | | | | | | |  |  |  |  |  |  |  |  |  |  |  |  |  |  |  |  |  |  |  |  |  |  |  |  |  |  |  |  |  |  |  |  |  |
|  | |  | | | **IQR** | | | | | 421.1 to 763.6 | | | | | | | | | | | | | | | | | | | 506.2 to 771.1 | | | | | | | | | | | | | | | | | | | | | | |  |  |  |  |  |  |  |  |  |  |  |  |  |  |  |  |  |  |  |  |  |  |  |  |  |  |  |  |  |  |  |  |  |
| **Figure S1 Statistics** | | | | | | | | | | | | | | | | | | | | | | | | | | | | | | | | | | | | | | | | | | | | | | | | |  |  |  |  |  |  |  |  |  |  |  |  |  |  |  |  |  |  |  |  |  |  |  |  |  |  |  |  |  |  |  |  |  |  |  |  |
|  | | **S1A** | | | **regression line equation** | | | | | Y = 4.692x + 400.4 | | | | | | | | | | | | | | | | | | |  | | | | | | | | | | | | | | | | | | | | |  |  |  |  |  |  |  |  |  |  |  |  |  |  |  |  |  |  |  |  |  |  |  |  |  |  |  |  |  |  |  |  |  |  |  |
|  | |  | | | **slope 95% CI** | | | | | -6.871 to 16.25 | | | | | | | | | | | | | | | | | | |  | | | | | | | | | | | | | | | | | | | | |  |  |  |  |  |  |  |  |  |  |  |  |  |  |  |  |  |  |  |  |  |  |  |  |  |  |  |  |  |  |  |  |  |  |  |
|  | |  | | | **Spearman r** | | | | | -0.03068 | | | | | | | | | | | | | | | | | | |  | | | | | | | | | | | | | | | | | | | | |  |  |  |  |  |  |  |  |  |  |  |  |  |  |  |  |  |  |  |  |  |  |  |  |  |  |  |  |  |  |  |  |  |  |  |
|  | |  | | | **95% CI** | | | | | -0.2333 to 0.1745 | | | | | | | | | | | | | | | | | | |  | | | | | | | | | | | | | | | | | | | | |  |  |  |  |  |  |  |  |  |  |  |  |  |  |  |  |  |  |  |  |  |  |  |  |  |  |  |  |  |  |  |  |  |  |  |
|  | | **S1B** | | |  | | | | | **Male** | | | | | | | | | | | | | | | | | | | **Female** | | | | | | | | | | | | | | | | | | | | | | | |  |  |  |  |  |  |  |  |  |  |  |  |  |  |  |  |  |  |  |  |  |  |  |  |  |  |  |  |  |  |  |  |
|  | |  | | | **Median** | | | | | 441.8 | | | | | | | | | | | | | | | | | | | 301.9 | | | | | | | | | | | | | | | | | | | | | | | |  |  |  |  |  |  |  |  |  |  |  |  |  |  |  |  |  |  |  |  |  |  |  |  |  |  |  |  |  |  |  |  |
|  | |  | | | **IQR** | | | | | 276.4 to 633.5 | | | | | | | | | | | | | | | | | | | 248.9 to 600.0 | | | | | | | | | | | | | | | | | | | | | | | |  |  |  |  |  |  |  |  |  |  |  |  |  |  |  |  |  |  |  |  |  |  |  |  |  |  |  |  |  |  |  |  |
| **Figure 2 Statistics** | | | | | | | | | | | | | | | | | | | | | | | | | | | | | | | | | | | | | | | | | | | | | | |  | | | | | | | | | | | | | |  | | | | | | | | | |  |  |  |  |  |  |  |  |  |  |  |  |  |  |
|  | | **2C** | | | **regression line equation** | | | | | Y = 0.04816x + 87.42 | | | | | | | | | | | | | | | | | | |  | | | | | | | | | | | | | |  | | | | | | | |  | | | | | | | | | | | | |  | | | | | | | | |  |  |  |  |  |  |  |  |  |  |  |  |
|  | |  | | | **slope 95% CI** | | | | | 0.03054 to 0.06579 | | | | | | | | | | | | | | | | | | |  | | | | | | | | | | | | | |  | | | | | | | |  | | | | | | | | | | | | |  | | | | | | | | |  |  |  |  |  |  |  |  |  |  |  |  |
|  | |  | | | **Spearman r** | | | | | 0.5021 | | | | | | | | | | | | | | | | | | |  | | | | | | | | | | | | | |  | | | | | | | |  | | | | | | | | | | | | |  | | | | | | | | |  |  |  |  |  |  |  |  |  |  |  |  |
|  | |  | | | **95% CI** | | | | | 0.3718 to 0.6129 | | | | | | | | | | | | | | | | | | |  | | | | | | | | | | | | | |  | | | | | | | |  | | | | | | | | | | | | |  | | | | | | | | |  |  |  |  |  |  |  |  |  |  |  |  |
|  | | **2D** | | | **regression line equation** | | | | | Y = 0.2611*X + 414.9 | | | | | | | | | | | | | | | | | | |  | | | | | | | | | | | | | |  | | | | | | | |  | | | | | | | | | | | | |  | | | | | | | | |  |  |  |  |  |  |  |  |  |  |  |  |
|  | |  | | | **slope 95% CI** | | | | | 0.1732 to 0.3490 | | | | | | | | | | | | | | | | | | |  | | | | | | | | | | | | | |  | | | | | | | |  | | | | | | | | | | | | |  | | | | | | | | |  |  |  |  |  |  |  |  |  |  |  |  |
|  | |  | | | **Spearman r** | | | | | 0.404 | | | | | | | | | | | | | | | | | | |  | | | | | | | | | | | | | |  | | | | | | | |  | | | | | | | | | | | | |  | | | | | | | | |  |  |  |  |  |  |  |  |  |  |  |  |
|  | |  | | | **95% CI** | | | | | 0.2607 to 0.5299 | | | | | | | | | | | | | | | | | | |  | | | | | | | | | | | | | |  | | | | | | | |  | | | | | | | | | | | | |  | | | | | | | | |  |  |  |  |  |  |  |  |  |  |  |  |
|  | | **2E** | | |  | | | | |  | | | | | | | | | | | | | **0 weeks** | | | | | | | | | | | | | | | | **6 weeks** | | | | | | | | | | | | | | | | | | | | | **12 weeks** | | | | | | | | | | | | | |  | | | | | | | | |  | |
|  | |  | | | **correlation coefficient r** | | | | | **CXM - PINP** | | | | | | | | | | | | | 0.3443 | | | | | | | | | | | | | | | | 0.4574 | | | | | | | | | | | | | | | | | | | | | 0.4424 | | | | | | | | | | | | | |  |  |  |  |  |  |  |  |  |  |  |
|  | |  | | | **correlation coefficient r** | | | | | **CXM - CTX** | | | | | | | | | | | | | 0.2464 | | | | | | | | | | | | | | | | 0.5023 | | | | | | | | | | | | | | | | | | | | | 0.1984 | | | | | | | | | | | | | |  |  |  |  |  |  |  |  |  |  |  |
|  | |  | | | **correlation coefficient r** | | | | | **CTX - PINP** | | | | | | | | | | | | | 0.5037 | | | | | | | | | | | | | | | | 0.4024 | | | | | | | | | | | | | | | | | | | | | 0.3361 | | | | | | | | | | | | | |  |  |  |  |  |  |  |  |  |  |  |
|  | |  | | | **95% CI** | | | | | **CXM - PINP** | | | | | | | | | | | | | 0.0783 to 0.5600 | | | | | | | | | | | | | | | | 0.2093 to 0.6451 | | | | | | | | | | | | | | | | | | | | | 0.1914 to 0.6341 | | | | | | | | | | | | | |  |  |  |  |  |  |  |  |  |  |  |
|  | |  | | | **95% CI** | | | | | **CXM - CTX** | | | | | | | | | | | | | -0.0280 to 0.4826 | | | | | | | | | | | | | | | | 0.2640 to 0.6776 | | | | | | | | | | | | | | | | | | | | | -0.0779 to 0.4433 | | | | | | | | | | | | | |  |  |  |  |  |  |  |  |  |  |  |
|  | |  | | | **95% CI** | | | | | **CTX - PINP** | | | | | | | | | | | | | 0.2683 to 0.6772 | | | | | | | | | | | | | | | | 0.1444 to 0.6042 | | | | | | | | | | | | | | | | | | | | | 0.0692 to 0.5537 | | | | | | | | | | | | | |  |  |  |  |  |  |  |  |  |  |  |
|  | | **2F** | | |  | | | | | **GDF-15** | | | | | | | | | | | | | **Leptin** | | | | | | | | | | | | | | | | **OC** | | | | | | | | | | | | | | | | | | | | | **Sclerostin** | | | | | | | | | | | | | |  |  |  |  |  |  |  |  |  |  |  |
|  | |  | | | **correlation coefficient r** | | | | | -0.0215 | | | | | | | | | | | | | -0.3468 | | | | | | | | | | | | | | | | -0.1423 | | | | | | | | | | | | | | | | | | | | | -0.0222 | | | | | | | | | | | | | |  |  |  |  |  |  |  |  |  |  |  |
|  | |  | | | **95% CI** | | | | | -0.2696 to 0.2297 | | | | | | | | | | | | | -0.5441 to  -0.1077 | | | | | | | | | | | | | | | | -0.3743 to 0.1081 | | | | | | | | | | | | | | | | | | | | | -0.2832 to 0.2423 | | | | | | | | | | | | | |  |  |  |  |  |  |  |  |  |  |  |
| **Figure 3 Statistics** | | | | | | |  |  | | | | | |  | | | | |  | | | | | | | | |  | | | | | | | | | |  | | | | | | |  | | | | | | | | |  | | | | | | | | | | |  | | | | | | | |  |  |  |  |  |  |  |  |  |  |  |  |
|  | **3B** | | |  | | | **Early**  **0** | | **Early**  **6** | | | | | | | | **Early**  **12** | | | | | | | | **Normal**  **0** | | | | | | | | | | | | **Normal**  **6** | | | | | | | | **Normal**  **12** | | | | | | | | | | | | | **Delayed**  **0** | | | | | | | | | **Delayed**  **6** | | | | | | | | | **Delayed**  **12** | | | | | | | |  |
|  |  | | | **Mean** | | | 506.6 | | 1092 | | | | | | | | 966.9 | | | | | | | | 441.5 | | | | | | | | | | | | 630.8 | | | | | | | | 818.0 | | | | | | | | | | | | | 592.6 | | | | | | | | | 1053 | | | | | | | | | 953.4 | | | | | | | |  |
|  |  | | | **95% CI** | | | 363.9 to 649.3 | | 804.8 to 1379 | | | | | | | | 662.1 t0 1272 | | | | | | | | 203.5 to 679.4 | | | | | | | | | | | | 399.9 to 861.8 | | | | | | | | 320.3 to 1316 | | | | | | | | | | | | | 458.0 to 727.1 | | | | | | | | | 792.2 to 1314 | | | | | | | | | 644.2 to 1263 | | | | | | | |  |
|  | **3C** | | |  | | | **GDF-15** | | | | | | | | **Leptin** | | | | | | | | | | | **OC** | | | | | | | | | | | | | | **Sclerostin** | | | | | | | | | | | | | | | | | | | | | |  | | | | | | |  | | | | | |  |  |  |  |  |  |  |  |  |  |
|  |  | | | **correlation coefficient r** | | | -0.2231 | | | | | | | | -0.2199 | | | | | | | | | | | 0.2173 | | | | | | | | | | | | | | 0.1807 | | | | | | | | | | | | | | | | | | | | | |  |  |  |  |  |  |  |  |  |  |  |  |  |  |  |  |  |  |  |  |  |  |  |
|  |  | | | **95% CI** | | | -0.4312 to 0.0106 | | | | | | | | -0.4284 to 0.0140 | | | | | | | | | | | -0.0185 to 0.4276 | | | | | | | | | | | | | | -0.0760 to 0.4124 | | | | | | | | | | | | | | | | | | | | | |  |  |  |  |  |  |  |  |  |  |  |  |  |  |  |  |  |  |  |  |  |  |  |
|  | **3D** | | |  | | | **GDF-15** | | | | | | | | **Leptin** | | | | | | | | | | | **OC** | | | | | | | | | | | | | | **Sclerostin** | | | | | | | | | | | | | | | | | | | | | |  | | | | | | |  | | | | | |  |  |  |  |  |  |  |  |  |  |
|  |  | | | **correlation coefficient r** | | | -0.0839 | | | | | | | | -0.3063 | | | | | | | | | | | 0.2560 | | | | | | | | | | | | | | 0.1093 | | | | | | | | | | | | | | | | | | | | | |  | | | | | | |  | | | | | |  |  |  |  |  |  |  |  |  |  |
|  |  | | | **95% CI** | | | -0.3224 to 0.1660 | | | | | | | | -0.5117 to 0.0630 | | | | | | | | | | | 0.0089 to 0.4706 | | | | | | | | | | | | | | -0.1617 to 0.3631 | | | | | | | | | | | | | | | | | | | | | |  | | | | | | |  | | | | | |  |  |  |  |  |  |  |  |  |  |
|  | **3E** | | |  | | | **GDF-15** | | | | | | | | **Leptin** | | | | | | | | | | | **OC** | | | | | | | | | | | | | | **Sclerostin** | | | | | | | | | | | | | | | | | | | | | |  | | | | | | |  | | | | | |  |  |  |  |  |  |  |  |  |  |
|  |  | | | **correlation coefficient r** | | | -0.1315 | | | | | | | | -0.0132 | | | | | | | | | | | 0.1155 | | | | | | | | | | | | | | -0.0391 | | | | | | | | | | | | | | | | | | | | | |  | | | | | | |  | | | | | |  |  |  |  |  |  |  |  |  |  |
|  |  | | | **95% CI** | | | -0.3209 to 0.0694 | | | | | | | | -0.2090 to 0.1838 | | | | | | | | | | | -0.0844 to 0.3054 | | | | | | | | | | | | | | -0.2452 to 0.1707 | | | | | | | | | | | | | | | | | | | | | |  | | | | | | |  | | | | | |  |  |  |  |  |  |  |  |  |  |
|  | **3F** | | | **regression line equation** | | | Y = 3.095*X - 24.82 | | | | | | | | | | |  | | | | | | | | |  | | | | | | | | | |  | | | | |  | | | | | |  | | | | | | | | | | | | | | |  | | | | | | | | | | | | | | | |  |  |  |  |  |  |
|  |  | | | **slope 95% CI** | | | 1.351 to 4.839 | | | | | | | | | | |  | | | | | | | | |  | | | | | | | | | |  | | | | |  | | | | | |  | | | | | | | | | | | | | | |  | | | | | | | | | | | | | | | |  |  |  |  |  |  |
|  |  | | | **Spearman r** | | | 0.4609 | | | | | | | | | | |  | | | | | | | | |  | | | | | | | | | |  | | | | |  | | | | | |  | | | | | | | | | | | | | | |  | | | | | | | | | | | | | | | |  |  |  |  |  |  |
|  |  | | | **95% CI** | | | 0.2663 to 0.6194 | | | | | | | | | | |  | | | | | | | | |  | | | | | | | | | |  | | | | |  | | | | | |  | | | | | | | | | | | | | | |  | | | | | | | | | | | | | | | |  |  |  |  |  |  |
|  | **3G** | | | **regression line equation** | | | Y = 1831*X - 33046 | | | | | | | | | | |  | | | | | | | | |  | | | | | | | | | |  | | | | |  | | | | | |  | | | | | | | | | | | | | | |  | | | | | | | | | | | | | | | |  |  |  |  |  |  |
|  |  | | | **slope 95% CI** | | | 1227 to 2435 | | | | | | | | | | |  | | | | | | | | |  | | | | | | | | | |  | | | | |  | | | | | |  | | | | | | | | | | | | | | |  | | | | | | | | | | | | | | | |  |  |  |  |  |  |
|  |  | | | **Spearman r** | | | 0.6343 | | | | | | | | | | |  | | | | | | | | |  | | | | | | | | | |  | | | | |  | | | | | |  | | | | | | | | | | | | | | |  | | | | | | | | | | | | | | | |  |  |  |  |  |  |
|  |  | | | **95% CI** | | | 0.4544 to 0.7645 | | | | | | | | | | |  | | | | | | | | |  | | | | | | | | | |  | | | | |  | | | | | |  | | | | | | | | | | | | | | |  | | | | | | | | | | | | | | | |  |  |  |  |  |  |
| **Figure 4 Statistics** | | | | | | | | | | | | | | | |  | | | | | |  | | | | | | | | | | | | |  | | | | | | | | | | |  | | | | | | | | | | | | | | |  | | | | | | | | | | |  |  |  |  |  |  |  |  |  |  |  |  |  |
|  | | **4A** | | |  | | | | | **Male 0** | | | | | | | | | | **Male 6** | | | | | | | | | | | | **Male 12** | | | | | | | | | | **Female 0** | | | | | | | | | | | | | | | **Female 6** | | | | | | | | | | | | **Female 12** | | | | | | | | | | | |  |  |  |  |
|  | |  | | | **Median** | | | | | 441.6 | | | | | | | | | | 904.7 | | | | | | | | | | | | 697.3 | | | | | | | | | | 312.1 | | | | | | | | | | | | | | | 501.5 | | | | | | | | | | | | 569.9 | | | | | | | | | | | |  |  |  |  |
|  | |  | | | **IQR** | | | | | 273.3 to 629.6 | | | | | | | | | | 507.0 to 1465 | | | | | | | | | | | | 335.3 to 1367 | | | | | | | | | | 251.4 to 618.4 | | | | | | | | | | | | | | | 380.9 to 873.4 | | | | | | | | | | | | 397.9 to 732.3 | | | | | | | | | | | |  |  |  |  |
|  | | **4B** | | |  | | | | | **Male** | | | | | | | | | | | | | | | | | | | **Female** | | | | | | | | | | | | | | | | | | | | | | | |  |  |  |  |  |  |  |  |  |  |  |  |  |  |  |  |  |  |  |  |  |  |  |  |  |  |  |  |  |  |  |  |
|  | |  | | | **Mean** | | | | | 645 | | | | | | | | | | | | | | | | | | | 271.8 | | | | | | | | | | | | | | | | | | | | | | | |  |  |  |  |  |  |  |  |  |  |  |  |  |  |  |  |  |  |  |  |  |  |  |  |  |  |  |  |  |  |  |  |
|  | |  | | | **95% CI** | | | | | 446.6 to 843.4 | | | | | | | | | | | | | | | | | | | 111.0 to 432.5 | | | | | | | | | | | | | | | | | | | | | | | |  |  |  |  |  |  |  |  |  |  |  |  |  |  |  |  |  |  |  |  |  |  |  |  |  |  |  |  |  |  |  |  |
|  | | **4C** | | |  | | | | | | **0 weeks** | | | | | | | | | | | | | | | | | | | **6 weeks** | | | | | | | | | | | | | | | | | | | | | | | **12 weeks** | | | | | | | | | | | | | | | | | | | | | | | |  |  |  |  |  |  |  |  |
|  | |  | | | **regression line equation** | | | | | | Y = 4.397*X + 406.0 | | | | | | | | | | | | | | | | | | | Y = 14.77*X + 526.4 | | | | | | | | | | | | | | | | | | | | | | | Y = -7.196*X + 1137 | | | | | | | | | | | | | | | | | | | | | | | |  |  |  |  |  |  |  |  |
|  | |  | | | **slope 95% CI** | | | | | | -7.038 to 15.83 | | | | | | | | | | | | | | | | | | | -4.431 to 33.96 | | | | | | | | | | | | | | | | | | | | | | | -32.33 to 17.94 | | | | | | | | | | | | | | | | | | | | | | | |  |  |  |  |  |  |  |  |
|  | |  | | | **Spearman r** | | | | | | -0.0395 | | | | | | | | | | | | | | | | | | | 0.0556 | | | | | | | | | | | | | | | | | | | | | | | -0.1431 | | | | | | | | | | | | | | | | | | | | | | | |  |  |  |  |  |  |  |  |
|  | |  | | | **95% CI** | | | | | | -0.2407 to 0.1649 | | | | | | | | | | | | | | | | | | | -0.1771 to 0.2823 | | | | | | | | | | | | | | | | | | | | | | | -0.3785 to 0.1097 | | | | | | | | | | | | | | | | | | | | | | | |  |  |  |  |  |  |  |  |
|  | | | **4D** | | | **regression line equation** | | | | | | | Y = -3.031*X + 597.1 | | | | | | | | | | | | | | | | | |  | | | | | | | | | |  | | | | | | | | | | | | | | |  | | | | | | | | | | | |  | | | | | | | | | |  |  |  |  |  |  |  |
|  | | |  | | | **slope 95% CI** | | | | | | | -22.77 to 16.71 | | | | | | | | | | | | | | | | | |  | | | | | | | | | |  | | | | | | | | | | | | | | |  | | | | | | | | | | | |  | | | | | | | | | |  |  |  |  |  |  |  |
|  | | |  | | | **Spearman r** | | | | | | | -0.1067 | | | | | | | | | | | | | | | | | |  | | | | | | | | | |  | | | | | | | | | | | | | | |  | | | | | | | | | | | |  | | | | | | | | | |  |  |  |  |  |  |  |
|  | | |  | | | **95% CI** | | | | | | | -0.3711 to 0.1737 | | | | | | | | | | | | | | | | | |  | | | | | | | | | |  | | | | | | | | | | | | | | |  | | | | | | | | | | | |  | | | | | | | | | |  |  |  |  |  |  |  |
|  | | | **4E** | | |  | | | | | | | **Tibia 0** | | | | | | | | **Tibia 6** | | | | | | | | | | | | **Tibia 12** | | | | | | | | | | | **Femur 0** | | | | | | | | | | | | | | | **Femur 6** | | | | | | | | | | | **Femur 12** | | | | | | | | | | | |  |  |  |
|  | | |  | | | **Median** | | | | | | | 403.6 | | | | | | | | 643.1 | | | | | | | | | | | | 454.4 | | | | | | | | | | | 397 | | | | | | | | | | | | | | | 944.8 | | | | | | | | | | | 738.2 | | | | | | | | | | | |  |  |  |
|  | | |  | | | **IQR** | | | | | | | 241.5 to 622.2 | | | | | | | | 377.3 to 1204 | | | | | | | | | | | | 290.2 to 972.5 | | | | | | | | | | | 273.3 to 634.8 | | | | | | | | | | | | | | | 602.7 to 1588 | | | | | | | | | | | 543.8 to 1631 | | | | | | | | | | | |  |  |  |
|  | | | **4F** | | |  | | | | | | | **Tibia** | | | | | | | | | | | | | | | | | | **Femur** | | | | | | | | | | | | | | | | | | | | | | | |  | | | | | | | | | | |  | | | | | | | | | | | | | |  |  |  |  |  |
|  | | |  | | | **Median** | | | | | | | 332 | | | | | | | | | | | | | | | | | | 444.9 | | | | | | | | | | | | | | | | | | | | | | | |  | | | | | | | | | | |  | | | | | | | | | | | | | |  |  |  |  |  |
|  | | |  | | | **IQR** | | | | | | | 40.88 to 589.6 | | | | | | | | | | | | | | | | | | 217.6 to 837.2 | | | | | | | | | | | | | | | | | | | | | | | |  | | | | | | | | | | |  | | | | | | | | | | | | | |  |  |  |  |  |
| **Figure 5 Statistics** | | | | | | | | | | | | | | | | | | | | | | | |  | | | | | | | | | | | |  |  |  |  |  |  |  |  |  |  |  |  |  |  |  |  |  |  |  |  |  |  |  |  |  |  |  |  |  |  |  |  |  |  |  |  |  |  |  |  |  |  |  |  |  |  |  |  |  |
|  | | | **5A** | | |  | | | | | | **DBS** | | | | | | | | | | | | | | | | | | | **Serum** | | | | | | | | | | | | | | | | | | | | | | | |  |  |  |  |  |  |  |  |  |  |  |  |  |  |  |  |  |  |  |  |  |  |  |  |  |  |  |  |  |  |
|  | | |  | | | **Median** | | | | | | 707.3 | | | | | | | | | | | | | | | | | | | 543.9 | | | | | | | | | | | | | | | | | | | | | | | |  |  |  |  |  |  |  |  |  |  |  |  |  |  |  |  |  |  |  |  |  |  |  |  |  |  |  |  |  |  |
|  | | |  | | | **IQR** | | | | | | 630.6 to 909.1 | | | | | | | | | | | | | | | | | | | 383.6 to 615.0 | | | | | | | | | | | | | | | | | | | | | | | |  |  |  |  |  |  |  |  |  |  |  |  |  |  |  |  |  |  |  |  |  |  |  |  |  |  |  |  |  |  |
|  | | | **5B** | | | **regression line equation** | | | | | | Y = 0.4217*X + 181.9 | | | | | | | | | | | | | | | | | | |  |  |  |  |  |  |  |  |  |  |  |  |  |  |  |  |  |  |  |  |  |  |  |  |  |  |  |  |  |  |  |  |  |  |  |  |  |  |  |  |  |  |  |  |  |  |  |  |  |  |  |  |  |  |
|  | | |  | | | **slope 95% CI** | | | | | | 0.2690 to 0.5743 | | | | | | | | | | | | | | | | | | |  |  |  |  |  |  |  |  |  |  |  |  |  |  |  |  |  |  |  |  |  |  |  |  |  |  |  |  |  |  |  |  |  |  |  |  |  |  |  |  |  |  |  |  |  |  |  |  |  |  |  |  |  |  |
|  | | |  | | | **Pearson r** | | | | | | 0.7175 | | | | | | | | | | | | | | | | | | |  |  |  |  |  |  |  |  |  |  |  |  |  |  |  |  |  |  |  |  |  |  |  |  |  |  |  |  |  |  |  |  |  |  |  |  |  |  |  |  |  |  |  |  |  |  |  |  |  |  |  |  |  |  |
|  | | |  | | | **95% CI** | | | | | | 0.4919 to 0.8528 | | | | | | | | | | | | | | | | | | |  |  |  |  |  |  |  |  |  |  |  |  |  |  |  |  |  |  |  |  |  |  |  |  |  |  |  |  |  |  |  |  |  |  |  |  |  |  |  |  |  |  |  |  |  |  |  |  |  |  |  |  |  |  |
|  | | | **5C** | | | **regression line equation** | | | | | | Y = 1.323*X - 234.4 | | | | | | | | | | | | | | | | | | |  |  |  |  |  |  |  |  |  |  |  |  |  |  |  |  |  |  |  |  |  |  |  |  |  |  |  |  |  |  |  |  |  |  |  |  |  |  |  |  |  |  |  |  |  |  |  |  |  |  |  |  |  |  |
|  | | |  | | | **slope 95% CI** | | | | | | 1.094 to 1.552 | | | | | | | | | | | | | | | | | | |  |  |  |  |  |  |  |  |  |  |  |  |  |  |  |  |  |  |  |  |  |  |  |  |  |  |  |  |  |  |  |  |  |  |  |  |  |  |  |  |  |  |  |  |  |  |  |  |  |  |  |  |  |  |
|  | | |  | | | **Spearman r** | | | | | | 0.8765 | | | | | | | | | | | | | | | | | | |  |  |  |  |  |  |  |  |  |  |  |  |  |  |  |  |  |  |  |  |  |  |  |  |  |  |  |  |  |  |  |  |  |  |  |  |  |  |  |  |  |  |  |  |  |  |  |  |  |  |  |  |  |  |
|  | | |  | | | **95% CI** | | | | | | 0.7555 to 0.9396 | | | | | | | | | | | | | | | | | | |  |  |  |  |  |  |  |  |  |  |  |  |  |  |  |  |  |  |  |  |  |  |  |  |  |  |  |  |  |  |  |  |  |  |  |  |  |  |  |  |  |  |  |  |  |  |  |  |  |  |  |  |  |  |
|  | | | **5D** | | | **regression line equation** | | | | | | Y = 0.8265*X - 129.0 | | | | | | | | | | | | | | | | | | |  |  |  |  |  |  |  |  |  |  |  |  |  |  |  |  |  |  |  |  |  |  |  |  |  |  |  |  |  |  |  |  |  |  |  |  |  |  |  |  |  |  |  |  |  |  |  |  |  |  |  |  |  |  |
|  | | |  | | | **slope 95% CI** | | | | | | 0.6697 to 0.9832 | | | | | | | | | | | | | | | | | | |  |  |  |  |  |  |  |  |  |  |  |  |  |  |  |  |  |  |  |  |  |  |  |  |  |  |  |  |  |  |  |  |  |  |  |  |  |  |  |  |  |  |  |  |  |  |  |  |  |  |  |  |  |  |
|  | | |  | | | **Spearman r** | | | | | | 0.8032 | | | | | | | | | | | | | | | | | | |  |  |  |  |  |  |  |  |  |  |  |  |  |  |  |  |  |  |  |  |  |  |  |  |  |  |  |  |  |  |  |  |  |  |  |  |  |  |  |  |  |  |  |  |  |  |  |  |  |  |  |  |  |  |
|  | | |  | | | **95% CI** | | | | | | 0.6247 to 0.9019 | | | | | | | | | | | | | | | | | | |  |  |  |  |  |  |  |  |  |  |  |  |  |  |  |  |  |  |  |  |  |  |  |  |  |  |  |  |  |  |  |  |  |  |  |  |  |  |  |  |  |  |  |  |  |  |  |  |  |  |  |  |  |  |
| **Figure S3 Statistics** | | | | | | | | | | | |  | | | | | | | | | | | | | | | | | | | | | |  |  |  |  |  |  |  |  |  |  |  |  |  |  |  |  |  |  |  |  |  |  |  |  |  |  |  |  |  |  |  |  |  |  |  |  |  |  |  |  |  |  |  |  |  |  |  |  |  |  |  |
|  | | | **S3A** | | | **regression line equation** | | | | | | Y = 0.5149*X + 280.6 | | | | | | | | | | | | | | | | | | |  |  |  |  |  |  |  |  |  |  |  |  |  |  |  |  |  |  |  |  |  |  |  |  |  |  |  |  |  |  |  |  |  |  |  |  |  |  |  |  |  |  |  |  |  |  |  |  |  |  |  |  |  |  |
|  | | |  | | | **slope 95% CI** | | | | | | 0.3285 to 0.7013 | | | | | | | | | | | | | | | | | | |  |  |  |  |  |  |  |  |  |  |  |  |  |  |  |  |  |  |  |  |  |  |  |  |  |  |  |  |  |  |  |  |  |  |  |  |  |  |  |  |  |  |  |  |  |  |  |  |  |  |  |  |  |  |
|  | | |  | | | **Spearman r** | | | | | | 0.7456 | | | | | | | | | | | | | | | | | | |  |  |  |  |  |  |  |  |  |  |  |  |  |  |  |  |  |  |  |  |  |  |  |  |  |  |  |  |  |  |  |  |  |  |  |  |  |  |  |  |  |  |  |  |  |  |  |  |  |  |  |  |  |  |
|  | | |  | | | **95% CI** | | | | | | 0.5286 to 0.8711 | | | | | | | | | | | | | | | | | | |  |  |  |  |  |  |  |  |  |  |  |  |  |  |  |  |  |  |  |  |  |  |  |  |  |  |  |  |  |  |  |  |  |  |  |  |  |  |  |  |  |  |  |  |  |  |  |  |  |  |  |  |  |  |
|  | | | **S3B** | | | **Bias** | | | | | | 361.9 | | | | | | | | | | | | | | | | | | |  |  |  |  |  |  |  |  |  |  |  |  |  |  |  |  |  |  |  |  |  |  |  |  |  |  |  |  |  |  |  |  |  |  |  |  |  |  |  |  |  |  |  |  |  |  |  |  |  |  |  |  |  |  |
|  | | |  | | | **SD of bias** | | | | | | 242.8 | | | | | | | | | | | | | | | | | | |  |  |  |  |  |  |  |  |  |  |  |  |  |  |  |  |  |  |  |  |  |  |  |  |  |  |  |  |  |  |  |  |  |  |  |  |  |  |  |  |  |  |  |  |  |  |  |  |  |  |  |  |  |  |
|  | | |  | | | **95% Limits of Agreement** | | | | | | -114.1 to 837.8 | | | | | | | | | | | | | | | | | | |  |  |  |  |  |  |  |  |  |  |  |  |  |  |  |  |  |  |  |  |  |  |  |  |  |  |  |  |  |  |  |  |  |  |  |  |  |  |  |  |  |  |  |  |  |  |  |  |  |  |  |  |  |  |
| **Figure 6 Statistics** | | | | | | | | | | | |  | | | | | | | | | | | | | | | | | | |  | | | | | | | | | | | | | | | | | | | | | | | | | | | | | | | | | | | | | | | | | | | | | | | | | | | | | |
|  | | | **6A** | | |  | | | | | | **CXM (pg/mL)** | | | | | | | | | | | | | | | | | | | **Days Post Injury** | | | | | | | | | | | | | | | | | | | | | | | |  |  |  |  |  |  |  |  |  |  |  |  |  |  |  |  |  |  |  |  |  |  |  |  |  |  |  |  |  |  |
|  | | |  | | | **Median** | | | | | | 720.5 | | | | | | | | | | | | | | | | | | | 54 | | | | | | | | | | | | | | | | | | | | | | | |  |  |  |  |  |  |  |  |  |  |  |  |  |  |  |  |  |  |  |  |  |  |  |  |  |  |  |  |  |  |
|  | | |  | | | **IQR** | | | | | | 532.2 to 1009 | | | | | | | | | | | | | | | | | | | 20.00 to 150.0 | | | | | | | | | | | | | | | | | | | | | | | |  |  |  |  |  |  |  |  |  |  |  |  |  |  |  |  |  |  |  |  |  |  |  |  |  |  |  |  |  |  |
|  | | | **6B** | | |  | | | | | | **Peak CXM** | | | | | | | | | | | | | | | | | | | **Days to Peak** | | | | | | | | | | | | | | | | | | | | | | | |  |  |  |  |  |  |  |  |  |  |  |  |  |  |  |  |  |  |  |  |  |  |  |  |  |  |  |  |  |  |
|  | | |  | | | **Median** | | | | | | 1158 | | | | | | | | | | | | | | | | | | | 35.5 | | | | | | | | | | | | | | | | | | | | | | | |  |  |  |  |  |  |  |  |  |  |  |  |  |  |  |  |  |  |  |  |  |  |  |  |  |  |  |  |  |  |
|  | | |  | | | **IQR** | | | | | | 792.9 to 1488 | | | | | | | | | | | | | | | | | | | 21 to 94 | | | | | | | | | | | | | | | | | | | | | | | |  |  |  |  |  |  |  |  |  |  |  |  |  |  |  |  |  |  |  |  |  |  |  |  |  |  |  |  |  |  |
| **Figure 7 Statistics** | | | | | | | | | | | |  | | | | | | | | | | | | | | | | | | |  | | | | | | | | | | | | | | | | | | | | | | | | | | | | | | | | | | | | | | | | | | | | | | | | | | | | | |
|  | | | **7A** | | | **regression line equation** | | | | | | Y = 5.720*X + 950.3 | | | | | | | | | | | | | | | | | | |  |  |  |  |  |  |  |  |  |  |  |  |  |  |  |  |  |  |  |  |  |  |  |  |  |  |  |  |  |  |  |  |  |  |  |  |  |  |  |  |  |  |  |  |  |  |  |  |  |  |  |  |  |  |
|  | | |  | | | **slope 95% CI** | | | | | | -1.701 to 13.14 | | | | | | | | | | | | | | | | | | |  |  |  |  |  |  |  |  |  |  |  |  |  |  |  |  |  |  |  |  |  |  |  |  |  |  |  |  |  |  |  |  |  |  |  |  |  |  |  |  |  |  |  |  |  |  |  |  |  |  |  |  |  |  |
|  | | |  | | | **Pearson r** | | | | | | 0.2392 | | | | | | | | | | | | | | | | | | |  |  |  |  |  |  |  |  |  |  |  |  |  |  |  |  |  |  |  |  |  |  |  |  |  |  |  |  |  |  |  |  |  |  |  |  |  |  |  |  |  |  |  |  |  |  |  |  |  |  |  |  |  |  |
|  | | |  | | | **95% CI** | | | | | | -0.06984 to 0.5063 | | | | | | | | | | | | | | | | | | |  |  |  |  |  |  |  |  |  |  |  |  |  |  |  |  |  |  |  |  |  |  |  |  |  |  |  |  |  |  |  |  |  |  |  |  |  |  |  |  |  |  |  |  |  |  |  |  |  |  |  |  |  |  |
|  | | | **7B** | | | **regression line equation** | | | | | | Y = 2.052*X + 425.7 | | | | | | | | | | | | | | | | | | |  |  |  |  |  |  |  |  |  |  |  |  |  |  |  |  |  |  |  |  |  |  |  |  |  |  |  |  |  |  |  |  |  |  |  |  |  |  |  |  |  |  |  |  |  |  |  |  |  |  |  |  |  |  |
|  | | |  | | | **slope 95% CI** | | | | | | -3.184 to 7.288 | | | | | | | | | | | | | | | | | | |  |  |  |  |  |  |  |  |  |  |  |  |  |  |  |  |  |  |  |  |  |  |  |  |  |  |  |  |  |  |  |  |  |  |  |  |  |  |  |  |  |  |  |  |  |  |  |  |  |  |  |  |  |  |
|  | | |  | | | **Pearson r** | | | | | | 0.1313 | | | | | | | | | | | | | | | | | | |  |  |  |  |  |  |  |  |  |  |  |  |  |  |  |  |  |  |  |  |  |  |  |  |  |  |  |  |  |  |  |  |  |  |  |  |  |  |  |  |  |  |  |  |  |  |  |  |  |  |  |  |  |  |
|  | | |  | | | **95% CI** | | | | | | -0.1966 to 0.4328 | | | | | | | | | | | | | | | | | | |  |  |  |  |  |  |  |  |  |  |  |  |  |  |  |  |  |  |  |  |  |  |  |  |  |  |  |  |  |  |  |  |  |  |  |  |  |  |  |  |  |  |  |  |  |  |  |  |  |  |  |  |  |  |
|  | | | **7C** | | | **regression line equation** | | | | | | Y = 0.1062*X + 95.01 | | | | | | | | | | | | | | | | | | |  |  |  |  |  |  |  |  |  |  |  |  |  |  |  |  |  |  |  |  |  |  |  |  |  |  |  |  |  |  |  |  |  |  |  |  |  |  |  |  |  |  |  |  |  |  |  |  |  |  |  |  |  |  |
|  | | |  | | | **slope 95% CI** | | | | | | -2.146 to 2.358 | | | | | | | | | | | | | | | | | | |  |  |  |  |  |  |  |  |  |  |  |  |  |  |  |  |  |  |  |  |  |  |  |  |  |  |  |  |  |  |  |  |  |  |  |  |  |  |  |  |  |  |  |  |  |  |  |  |  |  |  |  |  |  |
|  | | |  | | | **Spearman r** | | | | | | 0.1703 | | | | | | | | | | | | | | | | | | |  |  |  |  |  |  |  |  |  |  |  |  |  |  |  |  |  |  |  |  |  |  |  |  |  |  |  |  |  |  |  |  |  |  |  |  |  |  |  |  |  |  |  |  |  |  |  |  |  |  |  |  |  |  |
|  | | |  | | | **95% CI** | | | | | | -0.1500 to 0.4582 | | | | | | | | | | | | | | | | | | |  |  |  |  |  |  |  |  |  |  |  |  |  |  |  |  |  |  |  |  |  |  |  |  |  |  |  |  |  |  |  |  |  |  |  |  |  |  |  |  |  |  |  |  |  |  |  |  |  |  |  |  |  |  |
|  | | | **7D** | | |  | | | | | | **Normal** | | | | | | | | | | | | | | | | | | | **Delayed** | | | | | | | | | | | | | | | | | | | | | | | |  |  |  |  |  |  |  |  |  |  |  |  |  |  |  |  |  |  |  |  |  |  |  |  |  |  |  |  |  |  |
|  | | |  | | | **Mean** | | | | | | 40.11 | | | | | | | | | | | | | | | | | | | 48.07 | | | | | | | | | | | | | | | | | | | | | | | |  |  |  |  |  |  |  |  |  |  |  |  |  |  |  |  |  |  |  |  |  |  |  |  |  |  |  |  |  |  |
|  | | |  | | | **95% CI** | | | | | | 31.07 to 49.16 | | | | | | | | | | | | | | | | | | | 36.34 to 59.8 | | | | | | | | | | | | | | | | | | | | | | | |  |  |  |  |  |  |  |  |  |  |  |  |  |  |  |  |  |  |  |  |  |  |  |  |  |  |  |  |  |  |
|  | | | **7E** | | |  | | | | | | **<8** | | | | | | | | | | | | | | | | | | | **>9** | | | | | | | | | | | | | | | | | | | | | | | |  |  |  |  |  |  |  |  |  |  |  |  |  |  |  |  |  |  |  |  |  |  |  |  |  |  |  |  |  |  |
|  | | |  | | | **Median** | | | | | | 46.00 | | | | | | | | | | | | | | | | | | | 31.00 | | | | | | | | | | | | | | | | | | | | | | | |  |  |  |  |  |  |  |  |  |  |  |  |  |  |  |  |  |  |  |  |  |  |  |  |  |  |  |  |  |  |
|  | | |  | | | **IQR** | | | | | | 34.00 to 65.75 | | | | | | | | | | | | | | | | | | | 24.25 to 46.00 | | | | | | | | | | | | | | | | | | | | | | | |  |  |  |  |  |  |  |  |  |  |  |  |  |  |  |  |  |  |  |  |  |  |  |  |  |  |  |  |  |  |
|  | | | **7F** | | |  | | | | | | **Female** | | | | | | | | | | | | | | | | | | | **Male** | | | | | | | | | | | | | | | | | | | | | | | |  |  |  |  |  |  |  |  |  |  |  |  |  |  |  |  |  |  |  |  |  |  |  |  |  |  |  |  |  |  |
|  | | |  | | | **Mean** | | | | | | 1331 | | | | | | | | | | | | | | | | | | | 1049 | | | | | | | | | | | | | | | | | | | | | | | |  |  |  |  |  |  |  |  |  |  |  |  |  |  |  |  |  |  |  |  |  |  |  |  |  |  |  |  |  |  |
|  | | |  | | | **95% CI** | | | | | | 1093 to 1568 | | | | | | | | | | | | | | | | | | | 878.6 to 1219 | | | | | | | | | | | | | | | | | | | | | | | |  |  |  |  |  |  |  |  |  |  |  |  |  |  |  |  |  |  |  |  |  |  |  |  |  |  |  |  |  |  |
|  | | | **7G** | | |  | | | | | | **Female** | | | | | | | | | | | | | | | | | | | **Male** | | | | | | | | | | | | | | | | | | | | | | | |  |  |  |  |  |  |  |  |  |  |  |  |  |  |  |  |  |  |  |  |  |  |  |  |  |  |  |  |  |  |
|  | | |  | | | **Mean** | | | | | | 587 | | | | | | | | | | | | | | | | | | | 446.4 | | | | | | | | | | | | | | | | | | | | | | | |  |  |  |  |  |  |  |  |  |  |  |  |  |  |  |  |  |  |  |  |  |  |  |  |  |  |  |  |  |  |
|  | | |  | | | **95% CI** | | | | | | 432.2 to 741.8 | | | | | | | | | | | | | | | | | | | 298.2 to 594.5 | | | | | | | | | | | | | | | | | | | | | | | |  |  |  |  |  |  |  |  |  |  |  |  |  |  |  |  |  |  |  |  |  |  |  |  |  |  |  |  |  |  |
|  | | | **7H** | | |  | | | | | | **Female** | | | | | | | | | | | | | | | | | | | **Male** | | | | | | | | | | | | | | | | | | | | | | | |  |  |  |  |  |  |  |  |  |  |  |  |  |  |  |  |  |  |  |  |  |  |  |  |  |  |  |  |  |  |
|  | | |  | | | **Median** | | | | | | 26.00 | | | | | | | | | | | | | | | | | | | 45 | | | | | | | | | | | | | | | | | | | | | | | |  |  |  |  |  |  |  |  |  |  |  |  |  |  |  |  |  |  |  |  |  |  |  |  |  |  |  |  |  |  |
|  | | |  | | | **IQR** | | | | | | 20.00 to 132.5 | | | | | | | | | | | | | | | | | | | 21.50 to 96.00 | | | | | | | | | | | | | | | | | | | | | | | |  |  |  |  |  |  |  |  |  |  |  |  |  |  |  |  |  |  |  |  |  |  |  |  |  |  |  |  |  |  |
|  | | | **7I** | | |  | | | | | | **Femur** | | | | | | | | | | | | | | | | | | | **Tibia** | | | | | | | | | | | | | | | | | | | | | | | |  |  |  |  |  |  |  |  |  |  |  |  |  |  |  |  |  |  |  |  |  |  |  |  |  |  |  |  |  |  |
|  | | |  | | | **Mean** | | | | | | 1199 | | | | | | | | | | | | | | | | | | | 1184 | | | | | | | | | | | | | | | | | | | | | | | |  |  |  |  |  |  |  |  |  |  |  |  |  |  |  |  |  |  |  |  |  |  |  |  |  |  |  |  |  |  |
|  | | |  | | | **95% CI** | | | | | | 944.7 to 1453 | | | | | | | | | | | | | | | | | | | 991.8 to 1376 | | | | | | | | | | | | | | | | | | | | | | | |  |  |  |  |  |  |  |  |  |  |  |  |  |  |  |  |  |  |  |  |  |  |  |  |  |  |  |  |  |  |
|  | | | **7J** | | |  | | | | | | **Femur** | | | | | | | | | | | | | | | | | | | **Tibia** | | | | | | | | | | | | | | | | | | | | | | | |  |  |  |  |  |  |  |  |  |  |  |  |  |  |  |  |  |  |  |  |  |  |  |  |  |  |  |  |  |  |
|  | | |  | | | **Mean** | | | | | | 543.2 | | | | | | | | | | | | | | | | | | | 491 | | | | | | | | | | | | | | | | | | | | | | | |  |  |  |  |  |  |  |  |  |  |  |  |  |  |  |  |  |  |  |  |  |  |  |  |  |  |  |  |  |  |
|  | | |  | | | **95% CI** | | | | | | 399.4 to 686.9 | | | | | | | | | | | | | | | | | | | 334.3 to 647.7 | | | | | | | | | | | | | | | | | | | | | | | |  |  |  |  |  |  |  |  |  |  |  |  |  |  |  |  |  |  |  |  |  |  |  |  |  |  |  |  |  |  |
|  | | | **7K** | | |  | | | | | | **Femur** | | | | | | | | | | | | | | | | | | | **Tibia** | | | | | | | | | | | | | | | | | | | | | | | |  |  |  |  |  |  |  |  |  |  |  |  |  |  |  |  |  |  |  |  |  |  |  |  |  |  |  |  |  |  |
|  | | |  | | | **Median** | | | | | | 28 | | | | | | | | | | | | | | | | | | | 44.5 | | | | | | | | | | | | | | | | | | | | | | | |  |  |  |  |  |  |  |  |  |  |  |  |  |  |  |  |  |  |  |  |  |  |  |  |  |  |  |  |  |  |
|  | | |  | | | **IQR** | | | | | | 21.25 to 92.00 | | | | | | | | | | | | | | | | | | | 20.75 to 116.5 | | | | | | | | | | | | | | | | | | | | | | | |  |  |  |  |  |  |  |  |  |  |  |  |  |  |  |  |  |  |  |  |  |  |  |  |  |  |  |  |  |  |
