# Supplementary material for: Efficacy of biomarkers in the endochondral phase of fracture repair and healing in long bones: A clinical observational studys
Source: PLoS Med. 2025 Aug 29;22(8):e1004640. doi: 10.1371/journal.pmed.1004640 (PMC12410876; doi:10.1371/journal.pmed.1004640)
Supplement: S3 Data — Raw data from dried blood spots collected from patients with a fracture that were recruited from the Oregon Health & Science University (OHSU) level 1 academic trauma center and The Steadman Clinic under independent, but aligned, IRB-approved protocols (OHSU #00019234: TSC #2018-48). (PDF) [file pmed.1004640.s009.pdf]

ID	Age	Sex	Fracture	Fx Location	Operation	Open Fx	Date Weight-Bearing	classify (mRUST)	classify (3month <8 or >9)	days to peak	peak CXM (pg/mL)	dCXM (pg/mL)
OHSU_1	23	M	Tibia	Shaft	YES	NO	8/13/2019	delayed	>9	21	1098.18	448.155
OHSU_2	83	F	Tibia	Shaft	NO	NO	12/10/2019	normal	<8	78	1192.19	534.330
OHSU_3	24	M	Tibia	Shaft	YES	NO	4/24/2019	normal	>9	44	1472.31	861.525
OHSU_4	64	M	Femur	Subtroch	YES	NO	4/1/2019	early	>9	86	1308.38	521.505
OHSU_5	21	F	Tibia	Shaft	YES	NO	4/8/2019		>9	22	941.58	418.185
OHSU_6	33	M	Femur	shaft	YES	NO	4/2/2019	normal	>9	99	940.815	404.820
OHSU_7	65	F	Femur	Distal	YES	NO	6/4/2019		<8			
OHSU_8	41	M	Tibia	Shaft	YES	NO	6/25/2019	delayed	<8	264	1014.1	99.431
OHSU_9	20	M	Femur	Shaft	YES	NO	9/5/2019		<8	20	630.225	507.555
OHSU_10	25	M	Femur	Shaft	YES	NO	7/30/2019					
OHSU_11	68	F	Femur	Shaft	YES	NO	7/6/2019	delayed	<8	19	1287.36	908.190
OHSU_12	31	M	Tibia	Shaft	YES	YES	8/16/2019	delayed	>9	20	1301.63	605.475
OHSU_13	24	M	Femur	Shaft	YES	NO	8/14/2019		<8	21	1159.4	186.200
OHSU_14	54	M	Femur	Shaft	YES	NO	7/15/2019					
OHSU_15	27	F	Tibia	Shaft	YES	NO	10/1/2019	normal	>9	93	714.353	
OHSU_16	25	M	Tibia	Shaft	YES	NO	1/7/2020	normal	>9	98	712.823	
OHSU_17	38	F	Tibia	Plateau/Shaft	YES	NO	11/19/2019	normal	<8	82	1155.6	893.273
OHSU_18	78	M	Femur	interprosthetic shaft	YES	NO	10/8/2019	delayed	>9	25	1880.98	846.541
OHSU_19	48	F	Tibia	Shaft	YES	NO	12/3/2019		<8	16	1976.65	780.796
OHSU_20	46	F	Tibia	Tibia/ankle	YES	NO	11/15/2019	early	>9	24	1711.78	387.586
OHSU_21	20	F	Femur	Shaft	YES	NO	10/17/2019			22	2225.93	787.545
OHSU_22	48	M	Tibia	Shaft	YES	NO	1/13/2020		>9	31	1349.51	-60.840
OHSU_23	37	M	Tibia	Shaft	YES	YES	4/21/2020		>9	11	665.712	229.713
OHSU_24	34	M	Tibia	Shaft	YES	NO	1/6/2020	delayed	<8	488	1533.82	1099.609
OHSU_25	48	M	Tibia	Shaft	YES	NO	4/14/2020	delayed	<8			
OHSU_26	20	M	Tibia	Shaft	YES	NO	9/1/2020		>9	45	439.336	50.472
OHSU_27	29	M	Femur	Subtroch	YES	NO	7/7/2020	normal	>9	30	547.145	477.240
OHSU_28	22	F	Tibia	Shaft	YES	NO	9/1/2020	delayed	<8	634	1623.78	878.068
OHSU_29	41	F	Tibia	Shaft	YES	NO	2/9/2021	normal	>9	89	590.913	90.303
OHSU_30	76	F	Femur	Shaft	YES	NO	1/5/2021	delayed	<8	366	1301.59	649.157
OHSU_31	69	M	Femur	Subtroch	YES	NO	1/12/2021	normal	>9	94	1071.74	654.292
OHSU_32	70	M	Femur	Shaft	YES	NO	1/19/2021		>9	48	547.428	132.443
OHSU_33	29	M	Tibia	shaft	YES	YES	2/10/2021					
OHSU_34	29	M	Tibia	shaft	YES	NO	1/12/2021					
OHSU_35	44	F	Tibia	shaft	YES	NO	3/23/2021	delayed	<8	318	1181.2	
OHSU_36	76	F	Femur	distal	YES	NO	3/17/2021	delayed	<8	21	1813.41	604.994
OHSU_37	24	M	Femur	Subtroch	YES	NO	2/23/2021					
OHSU_38	34	M	Femur	distal	YES	NO	6/1/2021	delayed	<8	149	806.802	191.697
OHSU_39	34	F	Tibia	shaft with ankle extension	YES	NO	5/4/2021	delayed	<8	18	751.165	281.370
OHSU_40	57	F	Tibia	shaft with ankle extension	YES	NO	5/11/2021	normal	<8	172	1669.09	978.555
OHSU_41	74	F	Femur	distal femur / femoral shaft combo	YES	NO	3/16/2021			26	1381.29	1036.118
OHSU_42	45	F	Tibia	shaft	YES	NO	5/4/2021	normal	>9	338	2413.36	
OHSU_43	25	M	Tibia, Ankle	shaft	YES	YES	8/17/2021					
OHSU_44	20	F	Tibia, ankle	shaft	YES	NO	8/17/2021	normal	>9	16	1591.07	634.718
OHSU_45	25	F	femur	shaft	YES	NO	8/12/2021	normal	>9	26	1002.66	541.697
OHSU_46	27	M	tibia	shaft	YES	YES	8/12/2021					
OHSU_47	28	M	tibia	shaft	YES	YES	8/30/2021		>9	58	1149.2	534.301
OHSU_48	19	M	tibia	shaft	YES	NO	12/6/2021	normal	>9	22	1114.18	896.109
OHSU_49	31	F	tibia	shaft	YES	NO	12/7/2021	normal	>9	41	824.792	58.102
OHSU_50	46	F	tibia, ankle	shaft	YES	NO	2/8/2021	normal	>9	3	590.803	103.475
OHSU_51	64	M	segmental femur	subtrochanteric femur, femoral shaft in same femur	YES	NO	12/6/2021	delayed	<8	79	1279.59	241.058

ID	Cxm.1	Cxm.2	Cxm.3	Cxm.4	Cxm.5	Cxm.6	Cxm.7	Cxm.8	Cxm.9	Cxm.10	Cxm.11	Cxm.12
OHSU_1	650.025	1098.180	443.745	623.430	585.360	778.590	671.738	662.824	561.492			
OHSU_2	657.855	425.520	452.295	365.130	1192.185	600.030	530.685	861.660	542.138	726.030		
OHSU_3	610.785	945.315	1472.310	760.500								
OHSU_4	786.870		803.790	1308.375	1014.885	970.600						
OHSU_5	523.395	941.580	630.045									
OHSU_6	535.995	857.925	770.805	940.815	905.535							
OHSU_7			460.170	549.360								
OHSU_8	914.670	839.295	883.710	797.220	1014.101							
OHSU_9	122.670	630.225	397.215	55.140								
OHSU_10	583.470	1263.870										
OHSU_11	379.170	1287.360		927.974								
OHSU_12	696.150	229.860	1301.625	1030.500	826.148							
OHSU_13	973.200	186.300	1159.400	924.600								
OHSU_14	323.910	363.480										
OHSU_15			515.22	680.94	714.353	577.643	360.124					
OHSU_16		573.690	642.173	712.823	305.518	447.843						
OHSU_17	262.328	1913.873	885.105	1155.600	1007.743	759.300	690.912	922.642				
OHSU_18	1034.438	1880.978	1607.108	1368.360		683.584	555.284					
OHSU_19	1195.853	1976.648		1186.853								
OHSU_20	1324.193	1711.778		692.493								
OHSU_21	1438.380	2225.925	1101.462									
OHSU_22	1410.345	1349.505	1026.878	283.174								
OHSU_23	435.999	665.712		535.146	452.444							
OHSU_24	434.208	475.031	452.568	419.051	345.886	700.334	1533.817	1120.659	645.696	1304.500	983.300	407.700
OHSU_25												
OHSU_26	388.865	439.336	313.199									
OHSU_27	69.905	547.145	513.566	238.415								
OHSU_28	745.708	755.302	440.812	1026.352	970.043	1623.776						
OHSU_29	500.610	584.127	590.913	588.199	588.664							
OHSU_30	652.433	1171.985	788.922	1163.687	1301.590							
OHSU_31	417.448	678.579	720.522	1071.740	537.920	678.407	994.951	584.938	550.792	563.834		
OHSU_32	414.985	547.428	457.651	533.768								
OHSU_33	272.586	331.840										
OHSU_34												
OHSU_35		513.426	429.471	1181.204								
OHSU_36	1208.420	1813.414	1749.027									
OHSU_37												
OHSU_38	615.105	785.667	605.097	688.397	565.721	806.802	665.193					
OHSU_39	469.795	751.165	742.708	578.467	727.629	466.281						
OHSU_40	690.533	803.340	693.341	790.559	1669.088	1017.174	616.345					
OHSU_41	345.172	1381.290	840.856									
OHSU_42		925.637	1120.142	973.660	971.800	968.498	2413.360	1435.174	963.917	942.234		
OHSU_43		1600.838										
OHSU_44	956.354	1561.072	1172.765	1342.735	1220.615	1010.997						
OHSU_45	460.960	1002.657	840.082	686.452	818.312	347.706						
OHSU_46		953.499	962.890									
OHSU_47	614.900	873.450	1149.201	734.578								
OHSU_48	218.073	1114.182	998.168									
OHSU_49	766.690	817.258	824.792	749.925	511.200	494.300						
OHSU_50	487.328	590.803	526.517	578.932	349.220	295.997	483.202					
OHSU_51	1038.530	577.158	1279.588	1038.300	498.400							

ID	DOI	D1	D2	D3	D4	D5	D6	D7	D8	D9	D10	D11	D12
OHSU_1	3/20/2019	3/20/2019	4/10/2019	5/2/2019	6/20/2019	8/13/2019	9/24/2019	11/5/2019	12/30/2019	2/25/2020			
OHSU_2	4/1/2019	4/1/2019	4/16/2019	4/30/2019	5/14/2019	6/18/2019	7/30/2019	8/22/2019	10/1/2019	11/5/2019	12/10/2019		
OHSU_3	3/31/2019	4/2/2019	4/24/2019	5/14/2019	7/16/2019								
OHSU_4	3/31/2019	4/1/2019	4/22/2019	5/14/2019	6/25/2019	7/16/2019	10/22/2019						
OHSU_5	4/7/2019	4/8/2019	4/29/2019	5/28/2019									
OHSU_6	4/1/2019	4/2/2019	4/23/2019	5/21/2019	7/9/2019	9/5/2019							
OHSU_7	4/22/2019	5/8/2019	5/14/2019	6/4/2019	7/16/2019								
OHSU_8	5/2/2019	5/8/2019	5/28/2019	6/25/2019	8/20/2019	1/21/2020							
OHSU_9	6/5/2019	6/7/2019	6/25/2019	7/23/2019	9/5/2019								
OHSU_10	6/5/2019	6/6/2019	7/2/2019	7/30/2019									
OHSU_11	7/4/2019	7/9/2019	7/23/2019	8/13/2019	2/11/2020								
OHSU_12	7/11/2019	7/12/2019	7/13/2019	7/31/2019	9/12/2019	2/13/2020							
OHSU_13	7/10/2019	7/12/2019	7/13/2019	7/31/2019	10/1/2019								
OHSU_14	7/14/2019	7/15/2019	7/16/2019										
OHSU_15	8/11/2019	8/14/2019	8/15/2019	9/5/2019	10/1/2019	11/12/2019	1/7/2020	9/8/2020					
OHSU_16	8/6/2019	8/8/2019	9/5/2019	10/1/2019	11/12/2019	1/7/2020	9/8/2020						
OHSU_17	8/29/2019	8/30/2019	9/17/2019	10/8/2019	11/19/2019	2/11/2020	10/6/2020	4/27/2021	11/9/2021				
OHSU_18	9/13/2019	9/17/2019	10/8/2019	10/22/2019	10/29/2019	12/17/2019	2/11/2020	9/1/2020					
OHSU_19	9/8/2019	9/13/2019	9/24/2019	10/22/2019	12/3/2019								
OHSU_20	10/22/2019	10/25/2019	11/15/2019	12/12/2019	2/13/2020								
OHSU_21	10/16/2019	10/21/2019	11/7/2019	12/3/2019									
OHSU_22	10/26/2019	11/7/2019	11/26/2019	12/31/2019	3/3/2020								
OHSU_23	10/31/2019	11/5/2019	11/11/2019	11/25/2019	12/17/2019	2/4/2020							
OHSU_24	11/3/2019	11/6/2019	12/17/2019	1/28/2020	2/11/2020	2/25/2020	1/19/2021	3/5/2021	3/23/2021	4/27/2021	11/9/2021	5/31/2022	9/20/2022
OHSU_25	2/28/2020	3/3/2020	3/18/2020	4/14/2020	6/16/2020	9/16/2020							
OHSU_26	7/4/2020	7/7/2020	8/18/2020	9/29/2020									
OHSU_27	7/5/2020	7/8/2020	8/4/2020	9/22/2020	12/8/2020								
OHSU_28	7/24/2020	7/26/2020	10/13/2020	11/24/2020	12/30/2020	3/16/2021	4/19/2022						
OHSU_29	8/6/2020	8/12/2020	9/22/2020	11/3/2020	2/9/2021	5/11/2021							
OHSU_30	9/27/2020	9/28/2020	10/19/2020	11/10/2020	2/16/2021	9/28/2021							
OHSU_31	10/10/2020	10/12/2020	11/4/2020	12/1/2020	1/12/2021	3/9/2021	6/15/2021	9/7/2021	12/7/2021	4/26/2022	5/13/2022		
OHSU_32	12/2/2020	12/3/2020	1/19/2021	1/19/2021	3/23/2021								
OHSU_33	12/26/2020	12/30/2020	1/20/2021										
OHSU_34	12/31/2020	1/2/2021											
OHSU_35	1/9/2021	1/11/2021	2/9/2021	3/23/2021	11/23/2021								
OHSU_36	1/28/2021	2/1/2021	2/18/2021	10/27/2021									
OHSU_37	1/27/2021	2/1/2021	2/23/2021										
OHSU_38	2/7/2021	2/9/2021	3/2/2021	3/23/2021	5/4/2021	6/1/2021	7/6/2021	8/31/2021					
OHSU_39	2/19/2021	2/21/2021	3/9/2021	3/23/2021	5/4/2021	11/23/2021	2/22/2022						
OHSU_40	2/19/2021	2/21/2021	3/16/2021	3/30/2021	5/11/2021	8/10/2021	11/15/2021	3/15/2022					
OHSU_41	2/18/2021	2/24/2021	3/16/2021	4/6/2021									
OHSU_42	3/21/2021	3/22/2021	4/13/2021	5/4/2021	6/22/2021	7/27/2021	10/12/2021	2/22/2022	4/26/2022	7/26/2022	10/25/2022		
OHSU_43	6/28/2021	6/30/2021	7/20/2021										
OHSU_44	7/4/2021	7/11/2021	7/20/2021	8/17/2021	9/28/2021	1/4/2022	4/5/2022						
OHSU_45	8/12/2021	8/14/2021	9/7/2021	10/5/2021	11/9/2021	1/4/2022	4/5/2022	8/16/2022					
OHSU_46	8/11/2021	8/15/2021	8/31/2021	9/28/2021									
OHSU_47	8/29/2021	8/31/2021	9/21/2021	10/26/2021	12/7/2021								
OHSU_48	10/12/2021	10/14/2021	11/3/2021	12/6/2021	3/11/2022								
OHSU_49	11/10/2021	11/11/2021	11/30/2021	12/21/2021	2/1/2022	5/3/2022	11/1/2022						
OHSU_50	12/15/2021	12/16/2021	12/18/2021	1/12/2022	2/8/2022	3/22/2022	6/28/2022	7/12/2022					
OHSU_51	12/5/2021	12/7/2021	1/11/2022	2/22/2022	4/5/2022	7/19/2022							

ID	Days since Injury	V1	V2	V3	V4	V5	V6	V7	V8	V9	V10	V11	V12	mRUST	mRUST.1	mRUST.2	mRUST.3	mRUST.4	mRUST.5	mRUST.6	mRUST.7	mRUST.8	mRUST.9	mRUST.10
OHSU_1		0.00	21.00	43.00	92.00	146.00	188.00	230.00	285.00	342.00					4	5	6	10	11	11	11	12	13	
OHSU_2		0.00	15.00	29.00	43.00	78.00	120.00	143.00	183.00	218.00	253.00				4	5	6	7	8	10	12	13	13	14
OHSU_3		2.00	24.00	44.00	107.00										4	5	7	12						
OHSU_4		1.00	22.00	44.00	86.00	107.00	205.00								4	8	10	12	15	15				
OHSU_5		1.00	22.00	51.00											4	6	8							
OHSU_6		1.00	22.00	50.00	99.00	157.00									4	7	10	12	12					
OHSU_7		16.00	22.00	43.00	85.00										4	5	6	8						
OHSU_8		6.00	26.00	54.00	110.00	264.00									4	4	5	6	10					
OHSU_9		2.00	20.00	48.00	92.00										4		6	6						
OHSU_10		1.00	27.00	55.00											4		7	8						
OHSU_11		5.00	19.00	40.00	222.00										4	4		7						
OHSU_12		1.00	2.00	20.00	63.00	217.00									4	4	6	9	11					
OHSU_13		2.00	3.00	21.00	83.00										4	4	5	8						
OHSU_14		1.00	2.00												4	4								
OHSU_15		3.00	4.00	25.00	51.00	93.00	149.00	394.00							4	4	5	7	10	12	16			
OHSU_16		2.00	30.00	56.00	98.00	154.00	399.00								4	4	8	9	12	15				
OHSU_17		1.00	19.00	40.00	82.00	166.00	404.00	607.00	803.00						4	4	4	7	13	16	16	16		
OHSU_18		4.00	25.00	39.00	46.00	95.00	151.00	354.00							4	4	6	6	9	10	12			
OHSU_19		5.00	16.00	44.00	86.00										4	4	6	8						
OHSU_20		3.00	24.00	51.00	114.00										4	11	14	15						
OHSU_21		5.00	22.00	48.00											4		7							
OHSU_22		12.00	31.00	66.00	129.00										4	7	9	11						
OHSU_23		5.00	11.00	25.00	47.00	96.00									4	4	6	8	10					
OHSU_24		3.00	44.00	86.00	100.00	114.00	443.00	488.00	506.00	541.00	737.00	940.00	1052.00		4	7	8	8	9	12	13		13	
OHSU_25		4.00	19.00	46.00	109.00	201.00									4	4	6	8	11					
OHSU_26		3.00	45.00	87.00											4	7	10							
OHSU_27		3.00	30.00	79.00	156.00										4	8	10	14						
OHSU_28		2.00	81.00	123.00	159.00	235.00	634.00								4	7	8	11	12	14	16			
OHSU_29		6.00	47.00	89.00	187.00	278.00									4	6	9	12	14					
OHSU_30		1.00	22.00	44.00	142.00	366.00									4	4	5	10	15					
OHSU_31		2.00	25.00	52.00	94.00	150.00	248.00	332.00	423.00	563.00	580.00				4	6	7	10	13	14	15	16		
OHSU_32		1.00	48.00	48.00	111.00										4	7	8	11						
OHSU_33		4.00	25.00												4	7	7	10						
OHSU_34		2.00													4									
OHSU_35		2.00	31.00	73.00	318.00										4	7	8	8	12	16				
OHSU_36		4.00	21.00	272.00											4	4	4	6	8	9	9	11		
OHSU_37		5.00	27.00												4	6								
OHSU_38		2.00	23.00	44.00	86.00	114.00	149.00	205.00							4	4	4	7	8	10	12			
OHSU_39		2.00	18.00	32.00	74.00	277.00	368.00								4	4	7	8	8	9	13	13	14	
OHSU_40		2.00	25.00	39.00	81.00	172.00	269.00	389.00							4	5	5	8	12	14	15			
OHSU_41		6.00	26.00	47.00											4		5							
OHSU_42		1.00	23.00	44.00	93.00	128.00	205.00	338.00	401.00	492.00	583.00				4	8	10	11	13	15	16			
OHSU_43		2.00	22.00												4	6								
OHSU_44		7.00	16.00	44.00	86.00	184.00	275.00								4	4	6	10	13	14				
OHSU_45		2.00	26.00	54.00	89.00	145.00	236.00	369.00							4	7	10	11	12	14	16			
OHSU_46		4.00	20.00	48.00											4	4	6							
OHSU_47		2.00	23.00	58.00	100.00										4	5	8	11						
OHSU_48		2.00	22.00	55.00	150.00										4		9	13						
OHSU_49		1.00	20.00	41.00	83.00	174.00	356.00								4	6	8	10	16	16				
OHSU_50		1.00	3.00	28.00	55.00	97.00	195.00	209.00							4	4	6	9	12	13	14			
OHSU_51		2.00	37.00	79.00	121.00	226.00									4	7	8	9	11					
